# The Resistome and Mobilome of Multidrug-Resistant Staphylococcus sciuri C2865 Unveil a Transferable Trimethoprim Resistance Gene, Designated *dfrE*, Spread Unnoticed

**DOI:** 10.1128/mSystems.00511-21

**Published:** 2021-08-10

**Authors:** Elena Gómez-Sanz, Jose Manuel Haro-Moreno, Slade O. Jensen, Juan J. Roda-García, Mario López-Pérez

**Affiliations:** a Institute of Food Nutrition and Health, ETHZ, Zurich, Switzerland; b Área de Microbiología Molecular, Centro de Investigación Biomédica de La Rioja (CIBIR), Logroño, Spain; c Evolutionary Genomics Group, División de Microbiología, Universidad Miguel Hernández, San Juan, Alicante, Spain; d Infectious Diseases and Microbiology, School of Medicine, Western Sydney University, Sydney, New South Wales, Australia; e Antimicrobial Resistance and Mobile Elements Group, Ingham Institute for Applied Medical Research, Sydney, New South Wales, Australia; UCSF

**Keywords:** *Staphylococcus sciuri*, adaptation, comparative genomics, *dfrE*, dihydrofolate reductase, methicillin-resistant coagulase-negative staphylococci, mobile genetic elements, multidrug resistance, plasmid, trimethoprim, SCC*mec*, prophage, PICI, *S. sciuri* subspecies, intraspecies diversity, evolution, reservoir

## Abstract

Methicillin-resistant Staphylococcus sciuri (MRSS) strain C2865 from a stranded dog in Nigeria was trimethoprim (TMP) resistant but lacked formerly described staphylococcal TMP-resistant dihydrofolate reductase genes (*dfr*). Whole-genome sequencing, comparative genomics, and pan-genome analyses were pursued to unveil the molecular bases for TMP resistance via resistome and mobilome profiling. MRSS C2865 comprised a species subcluster and positioned just above the intraspecies boundary. Lack of species host tropism was observed. S. sciuri exhibited an open pan-genome, while MRSS C2865 harbored the highest number of unique genes (75% associated with mobilome). Within this fraction, we discovered a transferable TMP resistance gene, named *dfrE*, which confers high-level TMP resistance in Staphylococcus aureus and Escherichia coli. *dfrE* was located in a novel multidrug resistance mosaic plasmid (pUR2865-34) encompassing adaptive, mobilization, and segregational stability traits. *dfrE* was formerly denoted as *dfr_like* in *Exiguobacterium* spp. from fish farm sediment in China but escaped identification in one macrococcal and diverse staphylococcal genomes in different Asian countries. *dfrE* shares the highest identity with *dfr* of soil-related Paenibacillus anaericanus (68%). Data analysis discloses that *dfrE* has emerged from a single ancestor and places S. sciuri as a plausible donor. C2865 unique fraction additionally enclosed novel chromosomal mobile islands, including a multidrug-resistant pseudo-SCC*mec* cassette, three apparently functional prophages (*Siphoviridae*), and an SaPI4-related staphylococcal pathogenicity island. Since *dfrE* seems not yet common in staphylococcal clinical specimens, our data promote early surveillance and enable molecular diagnosis. We evidence the genome plasticity of S. sciuri and highlight its role as a resourceful reservoir for adaptive traits.

**IMPORTANCE** The discovery and surveillance of antimicrobial resistance genes (AMRG) and their mobilization platforms are critical to understand the evolution of bacterial resistance and to restrain further expansion. Limited genomic data are available on Staphylococcus sciuri; regardless, it is considered a reservoir for critical AMRG and mobile elements. We uncover a transferable staphylococcal TMP resistance gene, named *dfrE*, in a novel mosaic plasmid harboring additional resistance, adaptive, and self-stabilization features. *dfrE* is present but evaded detection in diverse species from varied sources geographically distant. Our analyses evidence that the *dfrE*-carrying element has emerged from a single ancestor and position S. sciuri as the donor species for *dfrE* spread. We also identify novel mobilizable chromosomal islands encompassing AMRG and three unrelated prophages. We prove high intraspecies heterogenicity and genome plasticity for S. sciuri. This work highlights the importance of genome-wide ecological studies to facilitate identification, characterization, and evolution routes of bacteria adaptive features.

## INTRODUCTION

Staphylococcus spp. are ubiquitous bacteria present in diverse ecological niches. They are opportunistic pathogens responsible for mild to life-threatening infections (1). Coagulase-negative staphylococci (CoNS), the major group within the genus, now represent one of the major nosocomial pathogens (2). Within this cluster, the Staphylococcus sciuri species group includes five species that are most often present as commensal animal-associated bacteria (3). The ubiquitous presence of S. sciuri represents a continuous source for contamination, colonization, and infection in animals and humans from different niches, including dust and hospital surfaces (4–12). S. sciuri is a natural reservoir of the ancestral β-lactam resistance *mecA* gene, and it is considered a source for dissemination of *mecA* via horizontal gene transfer (HGT) by the staphylococcal cassette chromosome (SCC*mec*) element to other staphylococcal species (13–15). S. sciuri is frequently multidrug resistant (MDR), and novel clinically relevant antimicrobial resistance (AMR) genes have been first detected in this species. This includes the multidrug resistance (PhLOPS phenotype) *cfr* gene (16), the macrolide/lincosamide/streptogramin B (MLS_B_) resistance *erm*(33) (17), the lincosamide/streptogramin A resistance *sal*(A) (18), the oxazolidinone/phenicol resistance *optrA* (19), or the coexistence of plasmid-located *cfr-optrA* (19) and the β-lactam resistance *mecA-mecC* in hybrid SCC*mec* elements (20, 21). Subsequently, early discovery of novel AMR genes in this species appears critical to constrain their further expansion into pathogens.

Mobile genetic elements (MGEs) play a key role in intra- and interspecies HGT of AMR and virulence determinants. In S. sciuri, their “mobilome” (pool of genes within MGEs) and diversity remain largely unknown. Particularly, staphylococcal phages are considered ubiquitous in this genus and constitute major contributors to genome modulation and plasticity (22). Yet, phages of S. sciuri have been reported only twice (23, 24). Former reports indicate that staphylococcal phages also contribute to the spread of AMR elements (25–29), including mobilization of the SCC*mec* (24, 27, 30, 31).

In staphylococci, resistance to trimethoprim (TMP) is mediated by any of the following acquired dihydrofolate reductases (Dfrs): DfrA (DfrS1), DfrD, DfrG, DfrK, and DfrF (32–37). These enzymes are TMP-insensitive variants of the intrinsic dihydrofolate reductase(s) (Dhfr), which converts dihydrofolate into tetrahydrofolate, essential in the synthesis of nucleic acids precursors. In this study, we identified and characterized the TMP resistance *dfrE* gene in canine MDR S. sciuri C2865 and determined its location in a novel MDR-mobilizable plasmid. We noticed that *dfrE* is already present in several species from varied sources geographically distant. Our analyses evidence that the *dfrE*-carrying element has emerged from a single ancestor and position S. sciuri as the donor species. We further identified C2865 complete “resistome” (pool of AMR determinants) and mobilome, which included novel chromosomal and extrachromosomal elements. Finally, comparative genomics of S. sciuri species revealed high intraspecies heterogenicity and high genome plasticity for methicillin-resistant S. sciuri (MRSS) C2865.

## RESULTS

### Sequencing approach reasoning and general characteristics of S. sciuri C2865 genome.

MRSS C2865 was chosen for whole-genome sequencing (WGS) as it harbored the highest number of detected AMR genes. A summary of MRSS C2865 sequencing and assembly data is shown in Table 1. Illumina assembly retrieved 341 contigs with a contig sum of 2,937,715 bp. Plasmids pUR2865-1 (2,559 bp) and pUR2865-2 (3,830 bp) were identified as single circularized contigs based on contig boundary redundancy. Several fragmented mobile elements were identified; however, multiple attempts to determine their entire structure failed. PacBio sequencing retrieved one single circular chromosomal contig of 2,913,767 bp plus one circularized 40,108-bp plasmid (pUR2865-34), making a sum of 2,953,875 bp. Dot plot analysis of Illumina versus PacBio assemblies is included in the supplemental material and Fig. S1. PacBio data were used for all annotations and downstream analyses, except for both small Illumina-sequenced plasmids.

**TABLE 1:** Sequencing and assembly data comparison of the S. sciuri C2865 genome processed with Illumina Miseq and with PacBio RSII

Data type	Parameter	Illumina MiSeq	PacBio RSII
Sequencing	No. of bp	662,993,478	2,162,733,205
	No. of reads	2,774,428	103,918
	Mean read length	239	20,811
	Avg genome coverage	225×	730×
Assembly	Total assembled sequence (bp)	2,937,715	2,953,875
	Total assembled contigs	341	2
	Mean contig size	8,615	1,488,849
	Maximum contig length	125,700	2,913,767
	Length of chromosome sequence (bp)	2,893,295	2,913,767
	G+C content (%)	32.7	32.5
	*N*_50_ contig length	37,104	2,913,767
	ORFs	3,270	3,097
	Gene density (no genes/kb)	1.13	1.06
	Coding (%)	91%	89%
	Median intergenic spacer (bp)	51	51
	Protein-coding genes	3,193	3,020
	Ribosomal RNAs: 16S, 23S, 5S	19	19
	Transfer RNAs	58	58
Mobile elements	Preidentified insertion sequences	22 (15 different)	78 (18 different)
	Prophage	∼2–3	3
	SCC*mec*	1	1
	Staphylococcal pathogenicity island	1	1
	Plasmid^*a*^	∼4	1

a Consensus (Illumina + PacBio data) = 3 plasmids. All other definitive values in text were taken from PacBio data.

10.1128/mSystems.00511-21.2 FIG S1 Dot-plot graphs of MRSS C2865 genome assembled of PacBio and of Illumina reads using D-Genies. The program searches all the query sequences aligned on the diagonal and calculates the target sequence coverage per identity bin (79). Left graph shows the genome-genome alignments using PacBio assembly as reference (chromosomal contig and plasmid contig) (*x* axes) with Illumina query (*y* axes), while right graph depicts the genome-genome alignments using Illumina-assembled contigs as reference (*x* axes) with PacBio query (*y* axes). The summary identity calculation (>75% identity, <75%, <50%, and <25% and no matches) is made on the target sequence (reference) and represents the percentage of the target genome in base pairs. Note: node 96 (5,513 bp) denotes plausible contamination after contig manual checking. Download FIG S1, EPS file, 0.5 MB. Copyright © 2021 Gómez-Sanz et al. 2021 Gómez-Sanz et al. https://creativecommons.org/licenses/by/4.0/ This content is distributed under the terms of the Creative Commons Attribution 4.0 International license.

### S. sciuri exhibits an open pan-genome while MRSS C2865 shows the highest unique genome content and constitutes a subgroup within the species.

**FIG 1:**
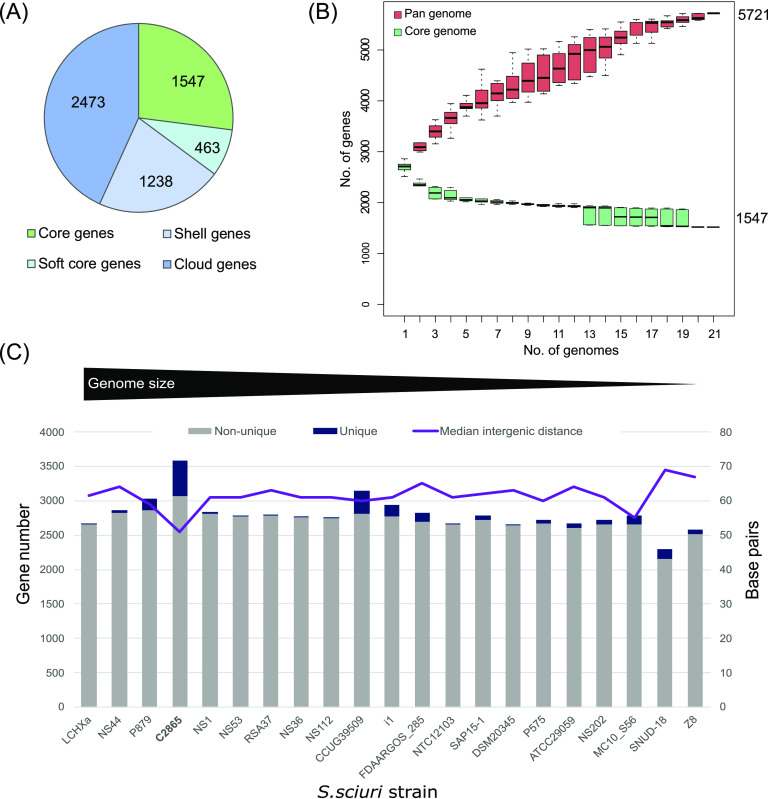
Pan-genome analyses for S. sciuri genomes at the species level (*n* = 21, 95% identity). (A) Pie chart showing the proportions of coding DNA sequence (CDS) in the core, soft core, shell, and cloud genomes. The parameters were defined as follows. Core genes: ≥99% of analyzed genomes, accessory genes: 1 to 99% (soft core 95 to 99%; shell 15 to 95%; cloud ≤15%). (B) Number of core genes (green) and total number of genes (pan genome) (red) curve for 21 S. sciuri strains. The upper and lower edges of the boxes indicate the first quartile (25th percentile of the data) and third quartile (75th percentile), respectively, of 1,000 random different input orders of the genomes. The central horizontal line indicates the sample median (50th percentile). (C) Bar chart of the total number of genes per genome indicating the number of non-unique and unique genes per genome (gray and dark blue, respectively) ordered by the genome size. Secondary axis displays the median intergenic distance per genome in base pairs.

Pan-genome analysis of 21 S. sciuri species genomes, which originated from human, animal, food, or environmental samples, including clinical isolates, revealed a pan-genome of 5,721 genes, comprising a core genome of 1.3 Mb with 1,547 shared proteins at 95% of identity (27% of genes) and a flexible genome of 4,174 (73%). The pan-genome curve did not level off, as the addition of each new genome increased the total gene pool. Instead, the core genome appeared to have reached a plateau (<1,600 genes) (Fig. 1A). This ability to acquire exogenous DNA indicates an open pan-genome for S. sciuri (Fig. 1B). This is further supported by a prevalence of “cloud genes” (genes found in up to 15% of the strains), which corresponds with approximately 43.2% of the pan-genome (Fig. 1A). S. sciuri C2865 harbored the highest number of genes (3,063) and the highest number of unique genes (521), whereas the average gene numbers were 2,707 and 95, respectively, considering the 21 strains (Fig. 1C). This was not correlated with the genome size or overall genome coding density (89%, median 89); however, MRSS C2865 encompassed the shortest median intergenic distance (Fig. 1C), mainly included within the flexible unique gene set (Table 2). Several novel chromosomal and extrachromosomal elements were detected (see below), which represented 75% of C2865 unique genes (390/521). Three plasmids were identified within the unique fraction: two small single resistance plasmids (pUR2865-1 and pUR2865-2) (Fig. S4, plus Text S1 for details) and a novel MDR plasmid, pUR2865-34 (see section below). Table 2 summarizes the most relevant characteristics of the MGEs identified.

**TABLE 2:** General characteristics of most relevant S. sciuri C2865 mobile genetic elements detected as consensus of sequencing data

Parameter	Value for:								
	pUR2865-1	pUR2865-2	pUR2865-34	SCC*mec*_C2865_	C2865-pp1	C2865-pp2	C2865-pp3	SscPIC2865	ψTn*554*
Contig coverage (compared to chromosome)	1.52	5.66	3.48	NA^*b*^	NA	NA	NA	NA	NA
Length of sequence (bp)	2,559	3,830	40,108	55,137	41,284	45,020	126,192	9,645	7,306
G+C content (%)	31.3	29.0	30.1	31.5	34.6	33.8	30.8	30.1	35.3
Protein-coding genes	2	3	41	61	62	64	166	21	6
Gene density (no. genes/kb)	0.78	0.78	1.02	1.11	1.50	1.42	1.32	2.18	0.82
Coding density (%)	67	89	78	85	95	94	89	87	96
Median intergenic spacer (size, bp)	851	203	85	53	9.5	11	25	55	6
Replication	RepC	RepC	RepA_N					Primase	
Recombinase^*a*^ (no.)		Res/Rec	Res/Rec (3)	LSR, Res/Rec	LSR	LSR	LSR, Y-Int/Rec (2)	Y-Int/Rec	Y-Int/Rec (2)
Resistance gene(s)	*lnu*(A)	*cat*_pC221_	*erm*(B), *aacA-aphD*, *dfr_like*, *tet*(K)	*mecA*, *tet*(S), *aadE*, *arsC*, *arsB*, *copB*, *arsAD*					*cadC*, *cadA*, *cadD*
Other genes of interest		*mob*	*ica*-locus variant			*abiF*	*hicA-hicB*, *ardA*	*mazF*	
Insertion sequences (family)			5 (IS*6*)	4 (IS*6*)			1 (IS*110*)		
Transfer RNAs (aa)							1 (Pro, TGG)		

a Res/Rec, serine recombinase (S-rec) of the resolvase family; LSR, large serine recombinase; Y-Int/Rec, tyrosine recombinase; aa, amino acids.

b NA, not applied.

10.1128/mSystems.00511-21.1 TEXT S1 This file includes a detailed description of methods, further methods not indicated in the main text, additional results, and an extended discussion. Download Text S1, PDF file, 0.5 MB. Copyright © 2021 Gómez-Sanz et al. 2021 Gómez-Sanz et al. https://creativecommons.org/licenses/by/4.0/ This content is distributed under the terms of the Creative Commons Attribution 4.0 International license.

10.1128/mSystems.00511-21.5 FIG S4 Graphical representation of the RCR plasmids detected in MRSS C2865 and related plasmids. Arrows denote the genes, length, and orientation. Gene colors other than gray represent the following functions (color): replication and/or mobilization (green), antimicrobial resistance (pink, yellow). Areas of nucleotide similarity plasmids are indicated in gray. (A) Nucleotide sequence comparative analysis of the three RCR plasmids detected in MRSS C2865: pUR2865-1, pUR2865-2, and pUR2865-int, which is integrated in the larger pUR2865-34 plasmid. (B) Nucleotide sequence comparative analysis of pC194-related pUR2865-1 and S. aureus pC194 (GenBank accession no. NC_002013). Estimated double-strand origin (*dso*) and single-strand origin (*sso*) of replication are indicated. (C) Nucleotide sequence comparative analysis of the pT181-related pUR2865-2, the integrated pUR2865-int, and the RCR family prototype S. aureus pT181 (GenBank accession no. J01764.1). Putative *dso* nick and *sso* sites for pUR2865-1 and pUR2865-2 are denoted (100). Origin-of-transfer (*oriT*) for pT181-related plasmids is also indicated. (D) Sequence comparison of closest plasmids to pUR2865-1 (> 80% ID, > 70% coverage). (E) Sequence comparison of plasmids closest to pUR2865-2 (> 80% ID, > 70% coverage). Download FIG S4, SVG file, 0.5 MB. Copyright © 2021 Gómez-Sanz et al. 2021 Gómez-Sanz et al. https://creativecommons.org/licenses/by/4.0/ This content is distributed under the terms of the Creative Commons Attribution 4.0 International license.

**FIG 2:**
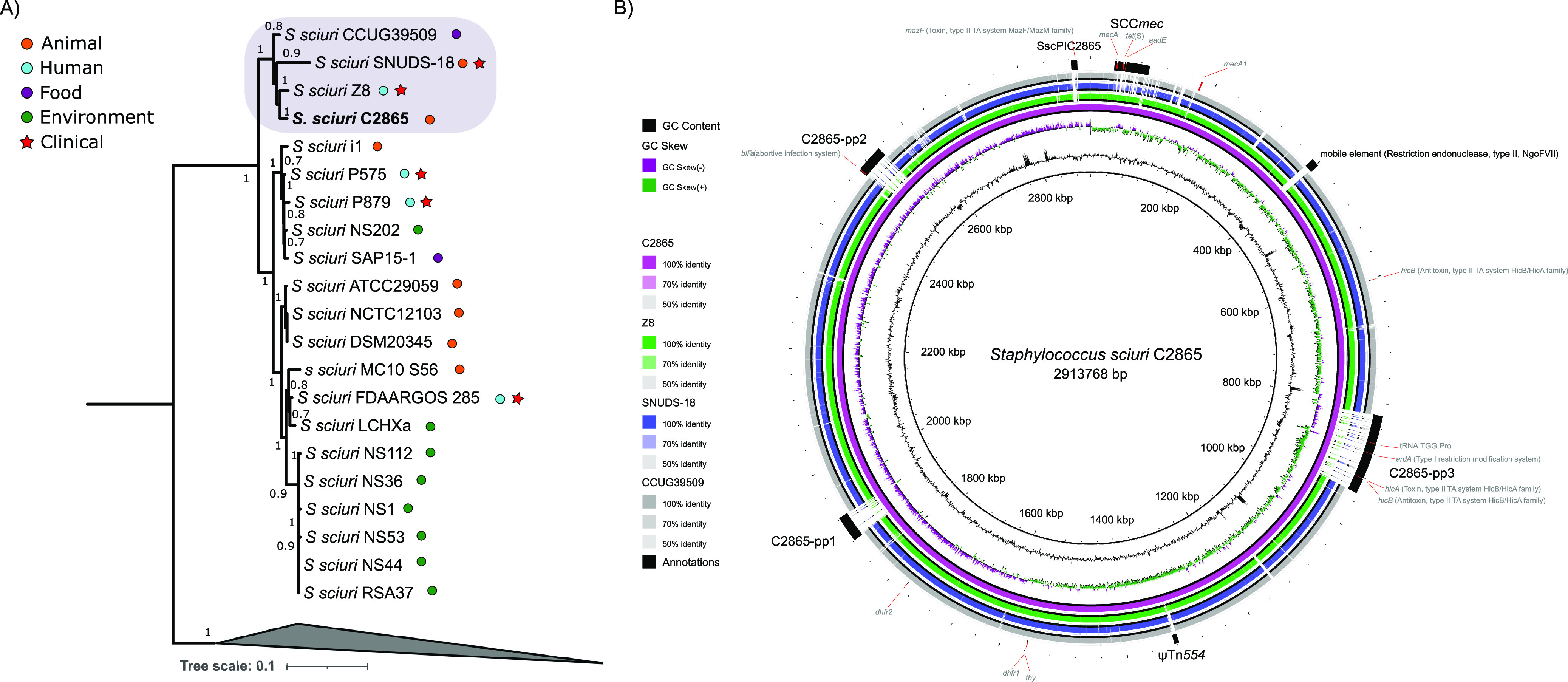
Phylogenomic analysis of S. sciuri and comparative diagram of MRSS C2865 closest relatives. (A) Maximum likelihood phylogenomic tree of S. sciuri C2865 and all 29 Staphylococcus sciuri group genomes deposited in the NCBI database (accessed until April 2018). Phylogenomic view of S. sciuri group species other than S. sciuri is collapsed as an outgroup to facilitate relatedness analysis of S. sciuri strains. Sample source is indicated as human, animal, food, and environment as well as whether the sample was isolated from a clinical infection. Note: strains S. sciuri DSM 20345 and S. sciuri NCTC12103 correspond to the same type strain (also known as ATCC 29062). (B) Comparative diagram of S. sciuri C2865 (pink ring) (reference genome) against its three closest genomes (S. sciuri Z8 [green ring], S. sciuri SNUD18 [blue], and S. sciuri CCUG39509 [gray]) as a set of concentric rings, where color indicates a BLAST match using BLAST Ring Image Generator (BRIG) (83). GC content and GC skew of reference genome is also displayed. Several features of interest are depicted, highlighting the unique presence of remarkable mobile genetic elements (SCC*mec*, C2865-pp3, ψTn*554*, C2865-pp1, C2865-pp2, SscPIC2865) in S. sciuri C2865. In addition, the genome location of the methicillin-susceptible *mecA1* gene, two intrinsic *dhfr* genes (*dhfr1* and *dhfr2*, the former next to a *thy* gene), a bacterial chromosomal *hicA*-antitoxin *hicB* (type II TA system), and the most relevant adaptive genes from the novel chromosomally located MGEs are displayed.

A refined S. sciuri phylogenomic tree revealed that MRSS C2865 formed a separate subgroup, together with MDR S. sciuri Z8, isolated from a human skin wound infection in China (38), MDR S. sciuri SNUDS-18, from a duckling with tremor in South Korea (39), and S. sciuri CCUG39509, from a sliced veal leg in Sweden (Fig. 2A). To obtain the most accurate phylogeny possible, we removed from the alignment all regions affected by recombination (see Materials and Methods). Average nucleotide identity (ANI) analysis evidenced that these four genomes shared ≥98% identity, while they displayed an ANI of 96% with respect to the major species cluster, positioning just above the species demarcation threshold (>95%) (Fig. S2). Genomic comparison of MRSS C2865 chromosome against its three closest relatives (Fig. 2C) evidenced the MRSS C2865 unique chromosomal islands. They corresponded to novel site-specific recombinase carrying MGEs (SCC*mec*, three prophages, staphylococcal pathogenicity island, transposon ψTn*554*) described in the following sections or in the supplemental material (for ψTn*554*, see also Fig. S3).

10.1128/mSystems.00511-21.3 FIG S2 Phylogenomic tree and heat map resultant from the average nucleotide identity (ANI) of 30 Staphylococcus sciuri group genomes, plus one Staphylococcus aureus and one Staphylococcus epidermidis reference genome used as outgroup for the genus level. Download FIG S2, EPS file, 0.3 MB. Copyright © 2021 Gómez-Sanz et al. 2021 Gómez-Sanz et al. https://creativecommons.org/licenses/by/4.0/ This content is distributed under the terms of the Creative Commons Attribution 4.0 International license.

10.1128/mSystems.00511-21.4 FIG S3 Graphical overview of MRSS C2865 ψTn*554* chromosomal region and comparative elements. The diagram depicts (i) MRSS C2865 ψTn*554* chromosomal region including the interrupted *radC* gene, (ii) its corresponding closest relative S. aureus 6850 (GenBank accession no. CP006706), and (iii) the chromosomal *radC* region and remnant 3′-end *radC* of phenicol and oxazolidinone resistant S. sciuri wo22_7 (KX982170) and S. sciuri MS11-3 (KX447571), respectively. Arrows denote the genes, length, and orientation. Gene colors other than gray represent the following functions (color): truncated or remnant chromosomal integration *radC* in Tn*554* family transposons (black), antimicrobial resistance (pink), metal resistance or transport (bright blue), transposition or recombination (yellow), ABC transporter ATP-binding proteins (faint brown). The 6-bp nucleotides resultant from Tn*554*-like integration are also depicted. Areas of nucleotide similarity (nblastn, >100 bp match, >85% identity) between strains/structures are indicated in gray. Download FIG S3, EPS file, 0.2 MB. Copyright © 2021 Gómez-Sanz et al. 2021 Gómez-Sanz et al. https://creativecommons.org/licenses/by/4.0/ This content is distributed under the terms of the Creative Commons Attribution 4.0 International license.

### Mosaic mobile adaptive elements within the novel plasmid pUR2865-34.

**FIG 3:**
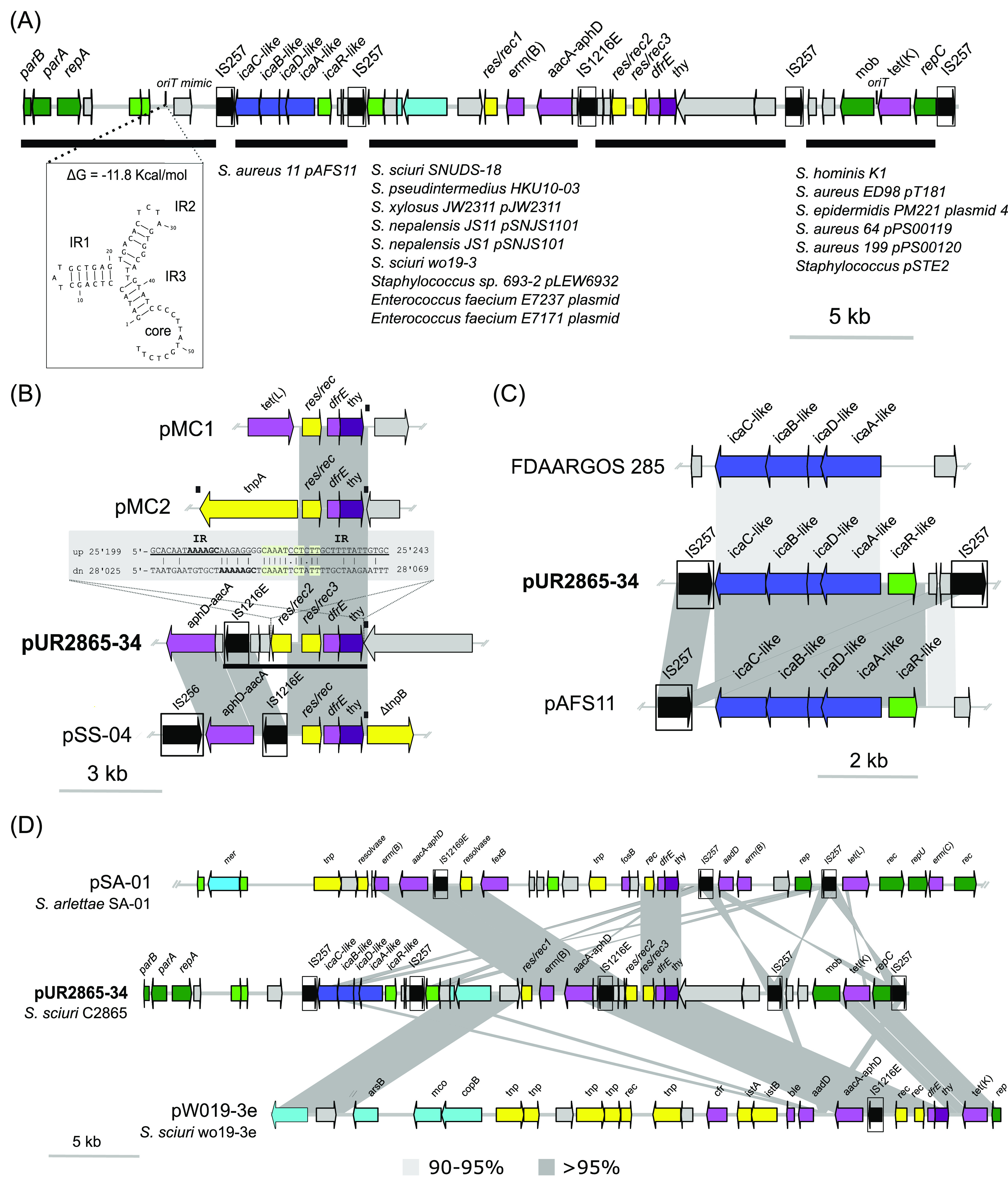
Comparative analysis of novel pUR2865-34 with the closest sequences in NCBI. Arrows denote the genes, length, and orientation. Gene colors other than gray represent the following genes of interest (color): antimicrobial resistance genes (pink), genes involve in metal resistance or transport (bright blue), intercellular adhesion gene cluster (*ica*) genes (navy blue), genes involved in transcription regulation (bright green), plasmid backbone genes (dark green), genes involved in transposition or recombination (yellow), and insertion sequences with defined imperfect inverted repeats (boxed and black). Areas of nucleotide similarity (nblastn, >100 bp match, >80% identity) between strains/structures are indicated in gray. For plasmids pSA-01, pAFS11, and S. sciuri FDAARGOS plasmid unnamed, only the area of interest is represented. (A) S. sciuri C2865 pUR2865-34 underlining its modular organization and indicating strain/plasmid sequences detected in NCBI with ≥50% coverage and ≥95% identity carrying those modules. The novel predicted secondary structure of the origin of conjugative transfer mimic (*oriT* mimic) is depicted, indicating the free energy of the DNA hairpins formation (ΔG). (B) Truncated *dfrE*-carrying transposon and immediate up- and downstream regions of S. sciuri C2865 pUR2865-34 and comparison with different *dfrE*-enclosing regions of *Exiguobacterium* sp. S3-2 pMC1 and pMC2, as well as S. sciuri GN5-1 pSS-04. Black boxes above the graphical display represent the Tn*3*-like characteristic 38-bp inverted repeats detected, involved in excision and integration of Tn*3* related elements. Region conserved in all *dfrE*-carrying elements except for those graphically represented is underlined in pUR2865-34 segment. (C) *ica*-locus variant-carrying region in S. sciuri C2865 pUR2865-34 and closest relatives deposited in NCBI database: S. aureus strain 11 pAFS11 and S. sciuri FDAARGOS plasmid. (D) Graphical comparison of pUR2865-34 and closest plasmids in the NCBI. Represented samples correspond to *S. arlettae* strain SA-01 plasmid pSA-01 and S. sciuri strain wo19-3e plasmid.

Plasmid pUR2865-34 was 40,108 bp in size and harbored 41 coding sequences (CDSs) (Fig. 3A). It showed a mosaic IS*6*-like-delimited modular organization (Fig. 3A), encompassing novel adaptive and backbone modules. pUR2865-34 cargo region enclosed a copy of the tetracycline efflux major facilitator superfamily (MFS) transporter gene *tet*(K), located within the small rolling-circle replication (RCR)-integrated plasmid, designated pUR2865-int. Sequence identity of RepC (83.1%) and composition of the double- (nick site: 5′-AAAACCGGaTACTCT/AATAGCCGGTT-3′, where capital letters denote conserved bases with respect to pT181, and slash the site of cleavage) and single-strand origin of replication (*dso* and *sso*, respectively), typical of RCR plasmids, classified this plasmid as a member of the pT181 family (Fig. 3A; Fig. S4). In addition, pUR2865-34 harbored a macrolide/lincosamide/streptogramin B resistance gene *erm*(B), coding for a 23S rRNA adenine *N*-6-methyltransferase and an aminoglycoside modifying *aacA-aphD* gene, encoding the bifunctional enzyme 6′-aminoglycoside *N*-acetyltransferase AAC(6′)-Ie aminoglycoside *O*-phosphotransferase APH(2″)-Ia, located immediately downstream of an IS257/IS*1216E* copy in the same orientation.

**FIG 4:**
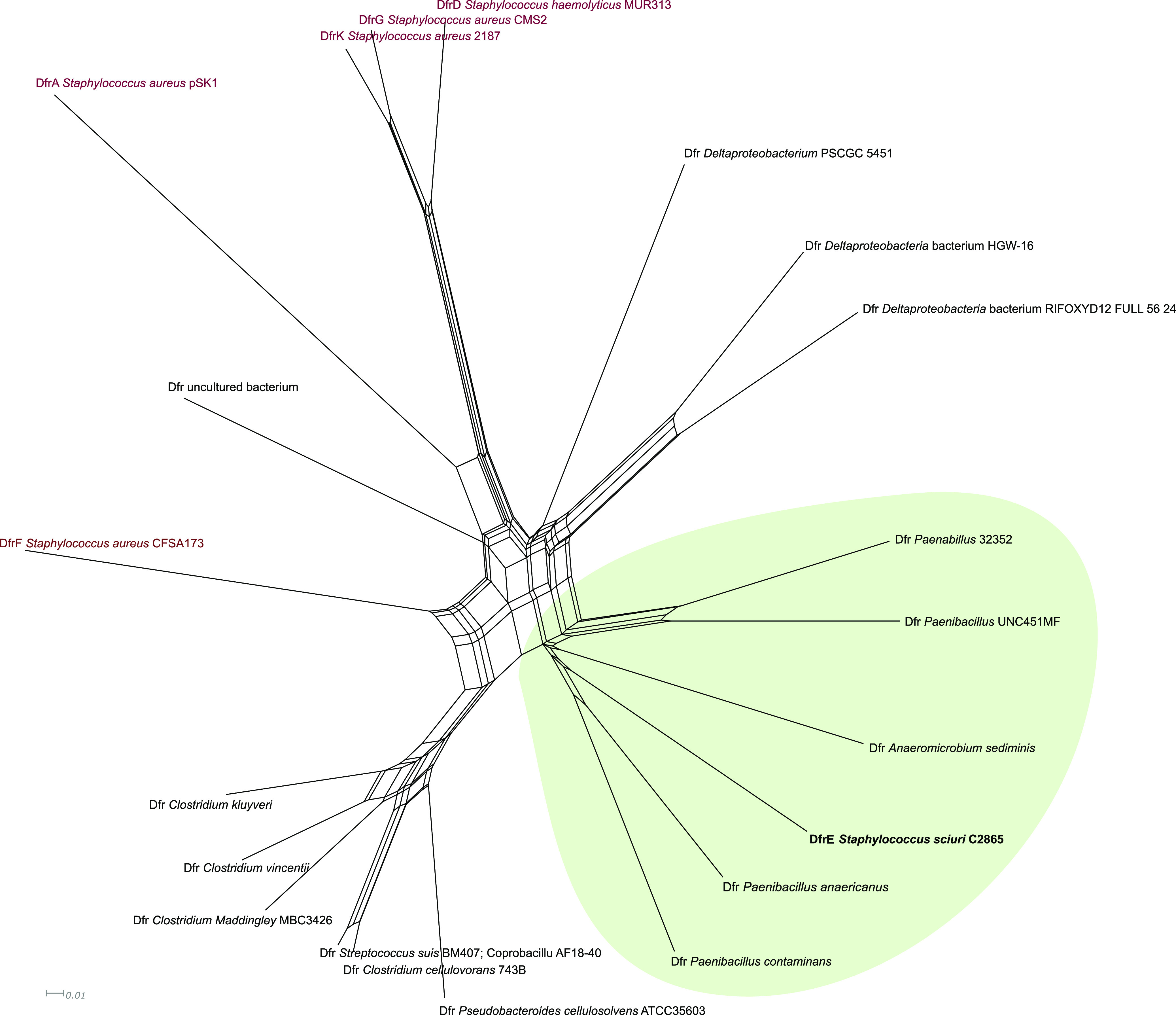
Phylogenetic network of aligned amino acid sequences of the dihydrofolate reductases (Dfr) closest to DfrE. The diagram illustrates all Dfr proteins derived from NCBI NR hits with a percentage of identity of >50% to DfrE of S. sciuri strain C2865 (bold), plus all trimethoprim resistance Dfrs described so far in staphylococci (DfrA, DfrD, DfrG, DfrK, DfrF) (dark red) (32–36). Identical Dfr proteins present in different bacterial classes were labeled with the identity of one representative per class (i.e., Firmicutes: Streptococcus suis BM407 and *Coprobacillus* sp. AF18-40). The amino acid branch clustering the trimethoprim resistance DfrE is highlighted with a faint green background.

Three serine recombinase (S-rec) genes of the resolvase/invertase subfamily (Text S1), named *res*/*rec1*, *res/rec2*, and *res/rec3*, were identified. Res/Rec2 and Res/Rec3 shared the highest amino acid identity to few staphylococcal isolates (four S. aureus, two S. sciuri, and one Staphylococcus arlettae strains) (>93% identity), while most entries corresponded to resolvases found in other Gram-positive bacteria (Macrococcus caseolyticus, *Exiguobacterium* sp., *Bacillus* sp., *Enterococcus* sp., *Lactococcus* sp., Streptococcus sp., *Lysinibacillus* sp., *Paenibacillus* sp., *Clostridium* sp.). A *dfr* gene, designated *dfrE*, and a thymidylate synthase gene (*thy*) were detected immediately downstream of *res*/*rec3*. DfrE shared 31.5 and 15.5% identity to two additional intrinsic Dhfr detected in S. sciuri C2865 genome, both located in conserved chromosomal regions lacking insertion sequences (ISs) or other mobile elements. DfrE was present in a limited number of strains at 100% sequence identity (Table 3). The *dfrE*-carrying strains corresponded to all the staphylococcal isolates harboring the *res*/*rec3* plus M. caseolyticus strain JCSC5402 and *Exiguobacterium* sp. strain S3-2, the latter harboring the *dfrE* gene in two different coexisting plasmids (Table 3). All *dfrE*-carrying staphylococcal genomes corresponded to WGS projects with direct submission to NCBI. In all these cases, the *dfrE* gene was unnoticed and included within MDR plasmids or plasmid-associated elements hosting additional AMR genes (Table 3). Of note, all strains harboring the overlooked *dfrE* were reported in Asian countries from animal-related or clinical human samples (Table 3). Only Yang et al. (40) denoted an identical gene, designated “*dfr_like*,” in *Exiguobacterium* sp. S3-2 from a fish farm sediment in China and proved its ability to confer TMP resistance in Gram-negative Escherichia coli DH5α. Hence, the role of this gene, here renamed *dfrE* (for *Exiguobacterium* spp), in TMP resistance in staphylococci remained open. DfrE revealed phylogenetically distant from the intrinsic Dhfr (Fig. S5) and was closest to the Dhfr of soil-related Paenibacillus anaericanus (68% identity) (Fig. 4). Within the staphylococcal TMP resistance genes, DfrE shared closer phylogenetic identity with TMP resistance DfrF, typical of enterococci and streptococci (Fig. S5).

**TABLE 3:** General features of *dfrE-*carrying strains deposited in the NCBI database and genetic platforms containing *dfrE*

Bacterial species	Location (plasmid ID)	Plasmid size (bp)	Tn*3* family element	Coverage/ID *dfrE* region^*b*^	Additional resistance pattern in *dfrE*-carrying element	Source	Country	TMP phenotype (μg/ml)	Reference	NCBI acc. no.
Staphylococcus sciuri C2865	Plasmid (pUR2865-34)	41,108	3′-end region	Reference	*erm*(B), *aacA-aphD*, *tet*(K)	Dog	Nigeria	MIC ≥ 4,096	This study	SAMN16182282 (PRJNA663854)
*Exiguobacterium* sp. S3-2	Plasmid (pMC2)	19,981	Complete + IRs^*a*^	47/99.95		Fish farm sediment	China	MIC > 1,024 in E. coli	40	KF648875
	Plasmid (pMC1)	71,276	3′-end region	46/99.85	*aadE*, *mefA*, *fexA*, *mph_like*, *mph*(B), *tet*(L)	Fish farm sediment	China	MIC > 1,024 in E. coli	40	KF648874
Macrococcus caseolyticus JCSC5402	Plasmid (pMCCL2)	80,545	3′-end region	100/100	*erm*(B), *aacA-aphD*, *mec*(B)	Chicken	Japan	Not tested	65	AP009486
Staphylococcus sciuri GN5-1	Plasmid (pSS-04)	18,496 (partial)	3′-end region	73/99.96	*erm*(B), *aacA-aphD*, *fexA*, *cfr*	Swine	China	Not indicated	Direct submission (2016)	KF129410
Staphylococcus sciuri wo19-3e	Genomic sequence (plasmid)	38,241	3′-end region	100/99.98	*aacA-aphD*, *tetK*, *aadD*, *ble*, *cfr*, *copB-mco*, *arsB*	Swine	China	Not indicated	Direct submission (2017)	KX982172
Staphylococcus aureus NTUH_3874	Plasmid (pNTUH_3874)	14,566	3′-end region	99/99.98	*erm*(B), *aacA-aphD*	Human blood	Taiwan	Not indicated	Direct submission (2016)	LC102479
Staphylococcus aureus GD1677	Chromosome (integrated plasmid)		3′-end region	99/100	*erm*(B), *aacA-aphD*, *aadE*, *cad*	Human	China	Not indicated	Direct submission (2017)	CP019595
Staphylococcus aureus FORC_039	Plasmid	35,415	3′-end region	99/99.98	*aacA-aphD*, *blaZ*, *cadAC* operon	Food	South Korea	Not indicated	Direct submission (2017)	CP015818
Staphylococcus aureus *FORC59*	Plasmid (pFORC59)	35,269	3′-end region	99/99.11	*erm*(B), *aacA-aphD*, *blaZ* operon, *cadX*	Human blood	South Korea	Not indicated	Direct submission (2017)	CP020355
Staphylococcus arlettae *SA-01*	Plasmid (pSA-01)	63,558	3′-end region	99/99.96	*erm*(B), *aacA-aphD*, *cfr*, *erm*(C), *tet*(L), *erm*(T), *aadD*, *fosD*, *fexB*, *ars*	Chicken	China	Not indicated	Direct submission (2017)	KX274135

a *Exiguobacterium* sp. S3-2 pMC2 harbors a primordial complete *dfrE*-carrying Tn*3* family element, flanked by two 38-bp imperfect inverted repeats (IRs) characteristic of transposases of the Tn*3* family, involved in excision and integration of the element.

b Coverage/ID in percentage of the remnant Tn-like *dfrE*-carrying element, which was defined as the region covering the IS*1216E*-*res/rec2-res/rec3-dfrE-thy* genes plus the 3′-end 38-bp IR characteristic of Tn*3* family elements (4,345 bp), using MRSS C2865 as reference.

10.1128/mSystems.00511-21.6 FIG S5 Neighbor-joining tree of aligned amino acid sequences of S. sciuri group dihydrofolate reductases. The alignment was performed with (i) the entire dihydrofolate reductase (Dhfr) protein(s) present in all S. sciuri species group genomes deposited in the NCBI database (accessed until April 2018), (ii) both Dhfrs present in S. sciuri strain C2865, and (iii) the amino acid sequence of all trimethoprim resistance dihydrofolate reductases (Dfr) described so far in staphylococci (DfrA, DfrD, DfrG, DfrK, DfrF) (32–36), as well as (iv) the Dhfr of trimethoprim-susceptible S. epidermidis ATCC 12228 (NC_004461) and S. aureus ATCC 25923 (Z16422) as reference, in blue color. Clusters enclosing the different TMP resistance Dfrs are colored differently. Download FIG S5, EPS file, 0.2 MB. Copyright © 2021 Gómez-Sanz et al. 2021 Gómez-Sanz et al. https://creativecommons.org/licenses/by/4.0/ This content is distributed under the terms of the Creative Commons Attribution 4.0 International license.

The *dfrE*-carrying region suggested a transposon-like structure encompassing an IS*1216E* copy, the *res/rec2* and *res/rec3* resolvase genes, and the *dfrE* and *thy* genes (4,345 bp) (Fig. 3B). This element seems to be a truncated version of the original *dfr_like*-carrying transposon (Tnp) of the *Tn*3 family detected in plasmid pMC2 of *Exiguobacterium* sp. S3-2 (40), as only the 38-bp imperfect inverted repeats (IRs) downstream of *thy* were present (Fig. 3B). Here, the pMC2-carrying Tn*3*-like *tnpA* gene, including the 38-bp upstream flanking IR, has been replaced by *res/rec2*. This Tn*3*-3′-end truncated region (IS*1216E-res/rec2*-*res/rec3-dfrE-thy-*IR) was conserved in all *dfrE*-enclosing strains except Staphylococcus sciuri GN5-1, which lacks the *res/rec2* plus immediate downstream coding sequences (CDSs) before the IS*1216E* (Table 3; Fig. 3B).

Plasmid pUR2865-34 harbored an additional IS*257*-flanked module, which included a variant of the intercellular adhesion gene cluster (*ica*ADBC) (Fig. 3C). This operon comprised the *icaA*, *icaD*, *icaB*, and *icaC* genes and the adjacent *ica*-locus repressor *icaR* gene (41). The *ica*-locus is involved in the early steps of biofilm formation (intercellular adhesion and cell agglutination) in staphylococci and appeared functional according to the CRAmod assay (Text S1; Fig. S6). This gene cluster shared the highest identity (97.9%) to the *ica*-locus variant of S. aureus pAFS11, an apramycin resistance *apmA*-carrying plasmid (GenBank accession no. FN806789.3) (Fig. 3C), followed by the *ica*ADBC cluster present in S. sciuri FDAARGOS_285 chromosome and respective plasmid (CP022046.2 and CP022047.2, respectively).

10.1128/mSystems.00511-21.7 FIG S6 Biofilm formation ability of *ica*-locus variant-carrying S. sciuri strains and transformants. (A) Biofilm formation capacity based on visual analysis of colony colors by a modified Congo red agar (CRAmod) assay. Spots were plated in duplicates. From left to right, (1) the positive controls (strong biofilm formers) S. aureus SA113 (DSM 4910) and S. aureus ATCC 25923 (DSM 1104), (2) negative-control (pUR2865-34 recipient) strain S. aureus RN4220, (3 and 4) the original S. sciuri *ica*-locus variant-carrying strains C2865, C2853, C2854, and C2855, (5) two selected S. aureus RN4220/pUR2865-34 transformants (S319 and S320). (B) Quantification of biofilm formation by crystal violet (CV) staining of adherent cells. The bar chart displays the results of six independent experiments in triplicates. Original S. sciuri *ica*-locus variant-carrying strains C2865, C2853, C2854, and C2855, two selected S. aureus RN4220/pUR2865-34 transformants (S319 and S320) as well as an *ica*-negative non-biofilm-producing methicillin-resistant Staphylococcus lentus strain (C3030) (101) are included. The three bars on the left show the control strains used: the strong biofilm formers S. aureus SA113 (DSM 4910) and S. aureus ATCC 25923 (DSM 1104), as well as S. aureus RN4220 strain. Significant differences by *t* test or analysis of variance (ANOVA; *P* < 0.01) are indicated with two stars on the compared cluster, i.e., *ica*-carrying strains (C2865, C2853, C2854, C2855, S319, S320; ns, not significant differences between values) and C3030, and *ica*-carrying strains and RN4220. Download FIG S6, EPS file, 0.7 MB. Copyright © 2021 Gómez-Sanz et al. 2021 Gómez-Sanz et al. https://creativecommons.org/licenses/by/4.0/ This content is distributed under the terms of the Creative Commons Attribution 4.0 International license.

The pUR2865-34 backbone region contains a type Ib partitioning system and a *repA_N* replication initiation gene (*repA*), characteristic of theta-replicating plasmids (42). It also harbors a single relaxase gene (*mob*) with an origin-of-transfer sequence (*oriT*) located immediately upstream as part of the integrated pUR2865-int (Fig. 3). In addition, an *oriT* mimic sequence (positions 6,077 to 6,132) similar to one recently described by Bukowski et al. (43) was observed proximal to the replication and partitioning genes (Fig. 3A). It is important to note that although pUR2865-34 does not carry any conjugative genes, these *oriT* regions can mediate the transfer of plasmid-carried phenotypes, such as resistance to trimethoprim, in the presence of conjugative elements.

MDR plasmids pSA-01 from S. arlettae strain SA-01 and pW019-3e from S. sciuri strain W019-3e resulted in the closest relatives to mosaic pUR2865-34 (Fig. 3D). MRSS C2853, C2854, and C2855 harbored a similar pUR2865-34 plasmid and enclosed the *dfrE* and *ica*-gene cluster (see Text S1 for details).

### The novel *dfrE* gene confers high-level resistance to trimethoprim.

All S. aureus RN4220 transformants carrying the different types of *dfrE*-carrying constructs exhibited a 2,048-fold increase in TMP resistance with respect to the empty S. aureus RN4220, irrespective of the presence of the natural or constitutive promoter located upstream of *dfrE* (Table 4). These results report the activity of the *dfrE* in S. aureus for the first time in literature. Likewise, all E. coli *dfrE*-carrying DH5aα transformants exhibited a 2,046- or 4,096-fold increase in TMP resistance with respect to the control (Table 4). Lack of synergistic activity was observed when *dfrE* and *thy* were cloned together into E. coli DH5α and S. aureus RN4220. Two S. aureus RN4220 transformants carrying entire pUR2865-34, named S319 and S320, exhibited a 2,048-fold increase in TMP resistance with respect to control strains and displayed additional resistance to tetracycline, erythromycin, clindamycin, gentamicin, kanamycin, and tobramycin, as confirmed by disc-diffusion agar tests.

**TABLE 4:** MIC values to trimethoprim (TMP) of original strains and respective DH5a and RN4220 constructs

Species	Strain	Characteristics, origin, or description	Reference or source	Antimicrobial resistance gene(s)	MIC (μg/ml) to TMP
Escherichia coli	DH5α	Recipient strain for electroporation, plasmid free	Promega		0.5
	DH5α/pBUS1-HC	DH5α with S. aureus*-*E. coli shuttle vector pBUS1-HC	96	*tet*(L)	0.5
	DH5α/pBUS1-Pcap-HC	DH5α with S. aureus*-*E. coli shuttle vector pBUS1-Pcap-HC	96	*tet*(L)	0.5
	DH5α/1B-1	DH5α/pBUS1-Pcap-HC/*dfrE* alone	This study	*tet*(L), *dfrE*	2,048
	DH5α/2A-1	DH5α/pBUS1-HC/+*dfrE*+^*a*^	This study	*tet*(L), *dfrE*	2,048
	DH5α/4A-1	DH5α/pBUS1-HC/+*dfrE*+*thy* +	This study	*tet*(L), *dfrE*	1,024
Staphylococcus aureus	DSM 2569	Reference strain for MIC (agar dilution method)	DSMZ^*b*^		1
	RN4220	Recipient strain for electroporation, plasmid free	DSMZ		1
	RN4220/pBUS1-HC	RN4220 with S. aureus*-*E. coli shuttle vector pBUS1-HC	This study	*tet*(L)	0.5
	RN4220/pBUS1-Pcap-HC	RN4220 with S. aureus*-*E. coli shuttle vector pBUS1-Pcap-HC	This study	*tet*(L)	0.5
	RN4220/1B-1	RN4220/pBUS1-Pcap-HC/*dfrE* alone	This study	*tet*(L), *dfrE*	2,048
	RN4220/2A-1	RN4220/pBUS1-HC/+*dfrE*+	This study	*tet*(L), *dfrE*	2,048
	RN4220/4A-1	RN4220/pBUS1-HC/+*dfrE*+*thy* +	This study	*tet*(L), *dfrE*	2,048
	RN4220/pUR2865-34	RN4220 with natural pUR2865-34 from S. sciuri C2865	This study	*tet(K)*, *erm(B)*, *aacA-aphD*, *dfrE*	2,048
Staphylococcus sciuri	C2865	Groin of dog in Nigeria	12	*mecA*, *tet*(K), *tet*(M), *tet*(S)^*c*^, *erm*(B), *lnu*(A), *aacA-aphD*, *ant6’*, *cat*_pC221_, *dfrE*	≥4,096
	C2853	Groin of dog in Nigeria	12	*mecA*, *tet*(K), *erm*(B), *aacA-aphD*, *dfrE*	≥4,096
	C2854	Groin of dog in Nigeria	12	*mecA*, *tet*(K), *tet*(M), *erm*(B), *aacA-aphD*, *cat*_pC223_, *dfrE*	≥4,096
	C2855	Groin of dog in Nigeria	12	*mecA*, *tet*(K), *tet*(M), *erm*(B), *lnu*(A), *aacA-aphD*, *cat*_pC221_, *dfrE*	≥4,096

a “+” represents *dfrE* immediate upstream or downstream region.

b Leibniz Institute DSMZ-German Collection of Microorganisms and Cell Cultures.

c The tetracycline resistance *tet*(S) was only detected after WGS in this study.

### Novel SCC*mec*_C2865_ element lacking formerly described chromosomal cassette recombinases.

**FIG 5:**
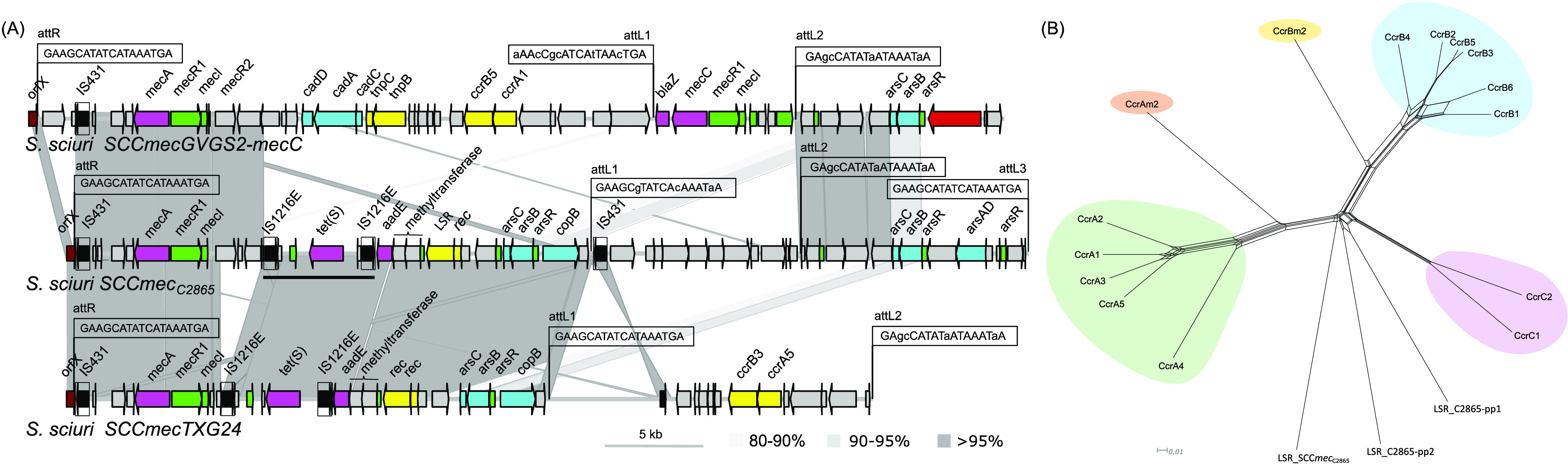
Graphical comparative analysis of the novel SCC*mec* and related large serine recombinases (LSR) detected in MRSS C2865. (A) Novel SCC*mec*_C2865_ (55,137 bp) and its closest elements in NCBI (SCC*mec*GVGS2-*mecC* of S. sciuri strain GVGS2 and SCC*mec*TXG24 of S. sciuri strain TXG24) (20, 45). Arrows denote the genes, length, and orientation. Gene colors other than gray represent the following genes of interest (color): antimicrobial resistance genes (pink), genes involved in metal resistance or transport (bright blue), genes involved in transcription regulation (bright green), genes involved in transposition or recombination (yellow), insertion sequences with defined imperfect inverted repeats (boxed and black), phage-related genes (red), and the SCC*mec* integration gene (dark red), which strictly does not belong to the SCC*mec* cassette, except for the *rlmH* 3′-end terminal 18-bp attachment site (ISS1 or *att*R 5′-GAAGCATATCATAAATGA-3′). The two perfect direct repeats found at both extremities of the cassette (*att*R 5′-GAAGCATATCATAAATGA-3′ and *att*L3 5′-GAAGCATATCATAAATGA-3′) are depicted. Two additional imperfect *att* sites (*att*L1 5′-GAAGCGTATCACAAATAA-3′ and *att*L2 5′-GAGCCATATAATAAATAA-3′) within SCC*mec*_C2865_ at base-pair positions 29,884 and 41,929 of the cassette, respectively, are also represented. Attachment sites detected in SCC*mec*GVGS2-*mecC* and SCC*mec*TXG24 are indicated. Unique bases with respect to *att*R, per SCC*mec* cassette, are represented in lower case. Areas of nucleotide similarity (nblastn, >100 bp match, >80% identity) between SCC*mec* cassettes are indicated in grayscale. (B) Phylogenetic network of aligned amino acid sequences of one representative staphylococcal chromosomal cassette recombinase (Ccr) per allotype described in Staphylococcus spp. and in *Macrococcus* spp. as well as the three LSRs present in S. sciuri C2865 genome comprising the Ccr consensus motif Y-[LIVAC]-R-[VA]-S-[ST]-x(2)-Q or Y-[LIVAC]-R-[VA]-S-[ST]-x(4)-Q. LSRs from S. sciuri C2865 originate from SCC*mec*_C2865_ (LSR_SCC_*_mec_*), prophage C2865-pp1 (LSR_C2865-pp1_) and prophage C2865-pp2 (LSR_C2865-pp2_).

A novel MDR SCC*mec* cassette, denominated SCC*mec*_C2865_, was identified at the 3′-end region of 23S rRNA [pseudouridine(1915)-*N*(3)]-methyltransferase RlmH gene (*rmlH*). SCC*mec*_C2865_ was 55,137 bp in size and contained a class A *mec* gene complex (IS*431-mecA-mecR1-mecI*) (Fig. 5A). None of the so-far-described staphylococcal or macrococcal chromosome cassette recombinase genes (*ccr*), the resultant large serine recombinases (LSR) of which are responsible for excision and integration of the cassette, were detected. SCC*mec*_C2865_ was delimited at both ends by characteristic SCC*mec*-flanking direct repeats (DRs) with typical insertion site sequences (ISS). Characteristic imperfect inverted repeats (IRs), required for CcrAB or CcrC recognition of the *att* sites, were located at the internal boundaries of the complete cassette (Fig. 5A; Text. S1) (44). The SCC*mec*_C2865_ *rlmH*-proximal region (*att*R-*att*L1) carried a class A *mec* gene complex and one transposon-like structure consisting of one tetracycline resistance gene *tet*(S), coding for a translation elongation factor G (EF-G) involved in tetracycline ribosomal protection, flanked by two IS*1216E* copies in the same orientation. No DRs were present, which could evidence its mobilization as a composite transposon (Tn) or translocatable unit (Fig. 5A). A streptomycin resistance gene *aadE*, coding for a 6-aminoglycoside adenylyltransferase, was detected immediately downstream of the 3′-end IS*1216E* copy. In addition, an arsenic resistance operon (*arsCBR*) and a copper resistance gene (*copB*), coding for a copper-translocating P-type ATPase, were located (Fig. 5A). SCC*mec*TXG24 from S. sciuri TXG24 and SCC*mec*GVGS2 from S. sciuri GVGS2 were identified as closest relatives to SCC*mec*_C2865_, sharing 57% and 40% coverage, respectively (Fig. 5A) (20, 45).

Protein domain analysis revealed a phage-related LSR, designated LSR_SCC*mec*_, which encompassed the typical PF00239, PF07508, and PF13408 domains, also present in Ccrs. As Ccrs can still transpose SCC*mec* elements when located elsewhere in the genome (46), the possible presence of *ccr* gene variants outside SCC*mec*_C2865_ was determined by a search of the translated genome of MRSS C2865 for the site-specific Ccr S-rec motif Y-[LIVAC]-R-[VA]-**S**-[ST]-x(2)-Q present in LSR. Two hits were identified: (i) LSR from prophage C2865-pp1, designated LSR_C2865-pp1_, and (ii) LSR from prophage C2865-pp2, designated LSR_C2865-pp2_ (see below). LSR_SCC_*_mec_* was identified only when we searched for consensus motif Y-[LIVAC]-R-[VA]-**S**-[ST]-x(4)-Q. Phylogenetic analyses evidenced these recombinases remarkably distant from known Ccrs. Yet, they revealed phylogenetically closer to CcrCs (Fig. 5B).

### Unique *Siphoviridae* prophages vB_SsS-C2865-pp1, vB_SsS-C2865-pp2, and vB_SsS-C2865-pp3 enclose adaptive features and excise the bacterial chromosome.

**FIG 6:**
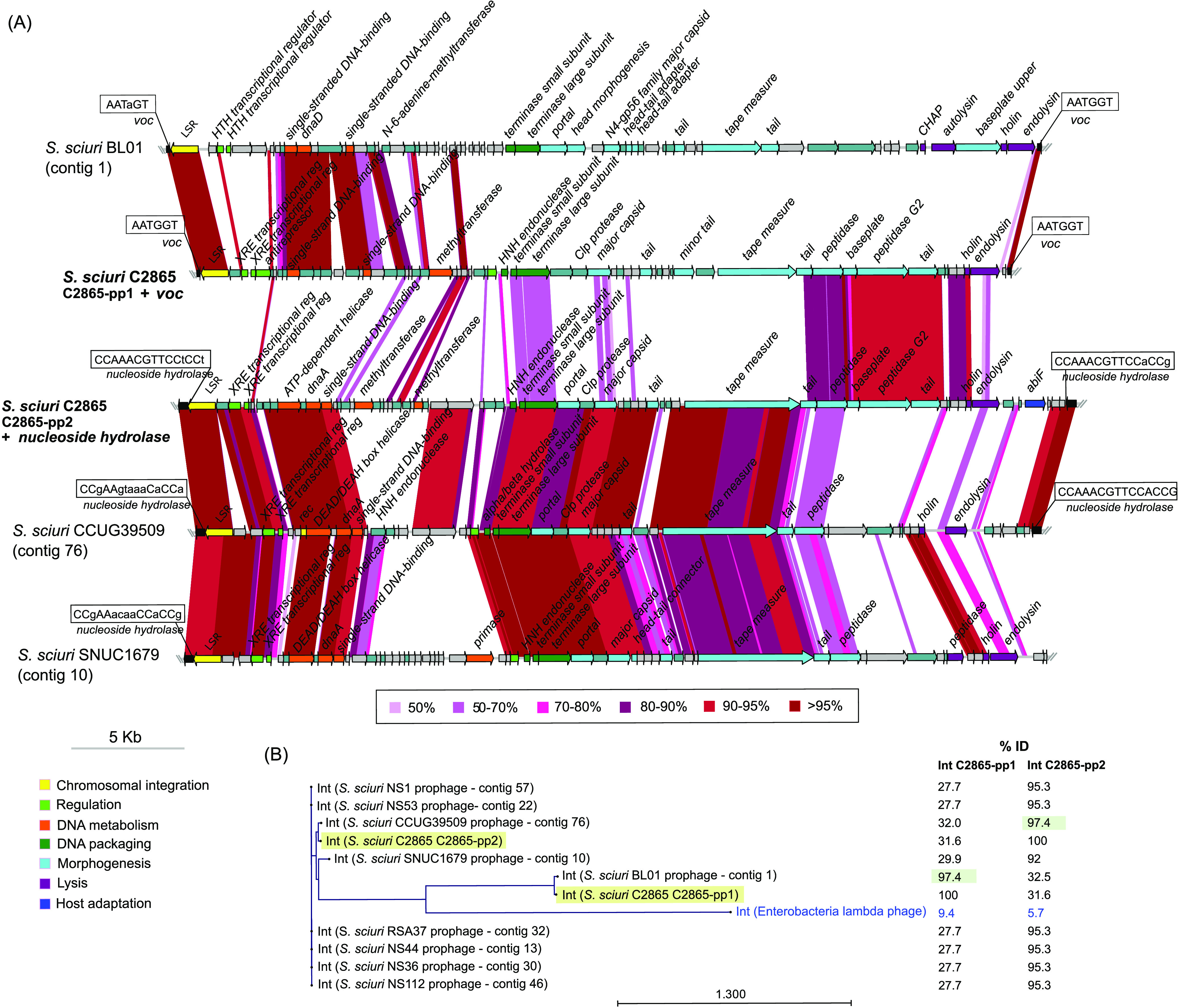
Graphical comparative analysis of prophages vB_SscS-C2865-pp1 (C2865-pp1) and vB_SscS-C2865-pp2 (C2865-pp2) and closest relatives. (A) Comparative analysis of the novel staphylococcal prophages C2865-pp1 and C2865-pp2 integrated in the chromosome of S. sciuri strain C2865 and its closest relatives based on (i) all staphylococcal phages deposited in the Viral RefSeq database and (ii) genetic elements enclosing the closest integrases in the NCBI NR database. Arrows denote the genes, length, and orientation. Gene colors other than gray (hypothetical proteins) and gray-turquoise (others) represent genes involved in the following processes (color): chromosomal integration (yellow), regulation (green), DNA metabolism (orange), DNA packaging (dark green), phage morphogenesis (bright blue), cell lysis (purple), host adaptation (navy blue). Areas of identity (tblastx, >50 aa match, >50% identity) between (pro)phages are indicated in the color scale. (B) Maximum likelihood phylogenetic tree of C2865-pp1 and C2865-pp2 integrases (Int), as well as all integrases sharing >80% amino acid identity with respect to either C2865-pp1 and C2865-pp2 integrases and integrase of *Enterobacteria* lambda phage, used as outgroup (colored in blue). C2865-pp1 and C2865-pp2 integrases are highlighted in faint yellow. Percentage of identity is indicated, with that sharing highest identity to respective integrase highlighted in faint green.

**FIG 7:**
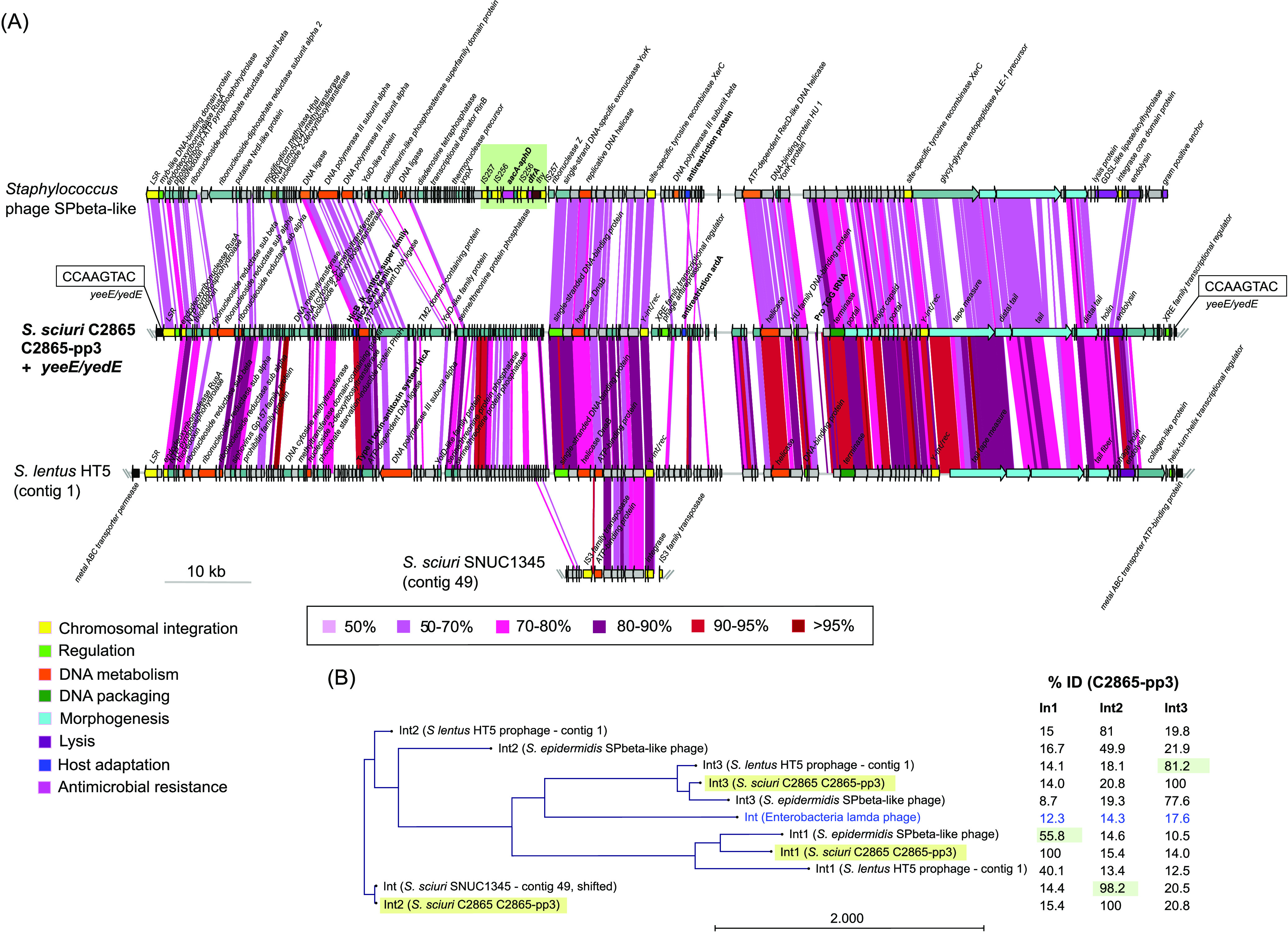
Graphical comparative analysis of prophage vB_SscS-C2865-pp3 (C2865-pp3) and closest relatives. (A) Comparative analysis of the novel staphylococcal prophage C2865-pp3 integrated in the chromosome of S. sciuri strain C2865 and its closest relatives based on (i) all staphylococcal phages deposited in the Viral RefSeq database as well as on (ii) genetic elements enclosing the closest integrases in the NCBI NR database. Arrows denote the genes, length, and orientation. Gene colors other than gray (hypothetical proteins) and gray-turquoise (others) represent genes involved in the following processes (color): chromosomal integration (yellow), regulation (green), DNA metabolism (orange), DNA packaging (dark green), phage morphogenesis (bright blue), cell lysis (purple), host adaptation (navy blue), antimicrobial resistance (pink). Areas of identity (tblastx, >50 aa match, >50% identity) between (pro)phages are indicated in the color scale. Staphylococcus phage SPbeta-like was linearized at position of interest. Area with green background denotes the region enclosing an independent mobile genetic region (flanked by two IS*257* copies in the same orientation), which encompasses the transposon Tn*4001* (IS*256*-*aacA/aphD*-IS*256*) as well as the trimethoprim resistance gene *dfrA*. (B) Maximum likelihood phylogenetic tree of the three integrases (Int) detected in C2865-pp3, as well as those present in its two closest (pro)phages and closest integrases (>80% amino acid identity) and integrase of *Enterobacteria* lambda phage, used as outgroup (colored in blue). Integrases from C2865-pp3 are highlighted in faint yellow. Percentage of identity is indicated, with that sharing highest identity to respective integrase highlighted in faint green.

We identified three novel prophages designated vB_SsS-C2865-pp1, vB_SsS-C2865-pp2, and vB_SsS-C2865-pp3, according to recommended guidelines (47, 48). ANI revealed that they differed from all available staphylococcal phages (Text S1; Fig. S7). Figures 6 and 7 show a comparative analysis of prophages C2865-pp1 and C2865-pp2, and C2865-pp3, respectively, with their closest relatives. Phylogenetic analysis of the closest integrases (LSR, tyrosine recombinases [Y-Int/Rec]) (>70% coverage, >80% identity), all recovered from phage-related elements, is displayed (Fig. 6B; Fig. 7B). This indicates the distribution of (pro)phages harboring this conserved feature. Full lysogenic modules were detected in all three phages (see Text S1 for details).

10.1128/mSystems.00511-21.8 FIG S7 Phylogenomic tree and heat map resultant from the average nucleotide identity (ANI) of a subset of 43 staphylococcal phages deposited in the Viral RefSeq NCBI database positioned within the same subbranch as C2865-pp1 to -pp3, plus the three prophages from S. sciuri strain C2865 colored in green. A phylogenomic tree including all 187 staphylococcal phages included in the analysis is represented in the upper left corner. Download FIG S7, PDF file, 2.5 MB. Copyright © 2021 Gómez-Sanz et al. 2021 Gómez-Sanz et al. https://creativecommons.org/licenses/by/4.0/ This content is distributed under the terms of the Creative Commons Attribution 4.0 International license.

C2865-pp1 was 41,283 bp long and consisted of 62 CDSs, of which 26 (41.9%) had predicted functions. C2865-pp1 had a GC content of 34.6% and was integrated truncating a VOC family-protein gene, 1,880,677 bp downstream of *dnaA*. The structural gene distribution of C2865-pp1 shared a typical phage modular organization. Characteristic phage gene groups involved in lysogeny (integrase, repressors, antirepressor; see Text S1 for details), DNA metabolism (single-strand DNA binding, methyltransferase), packaging (HNH endonuclease, terminases TerS, TerL), morphogenesis (major capsid, tail fiber, tape measure), and cell lysis (peptidase, holin, endolysin) were detected from left to right arm (Fig. 6A). Chromosomal integration of C2865-pp1 generated a 6-bp perfect DR (*attL* and *attR*) (5′-AATGGT-3′) (Fig. 6A) at its boundaries.

C2865-pp2 was 45,020 bp and consisted of 64 CDSs, of which 27 (42.2%) had predicted functions. C2865-pp2 had a GC content of 33.8% and was integrated truncating a nucleoside hydroxylase gene, 2,501,758 bp downstream of *dnaA*. C2865-pp2 CDSs were arranged in functional modules in synteny with those detected in C2865-pp1. Unusually, an abortive infection bacteriophage resistance gene, designated *abiF* (Abi_2 family, PF07751), was detected at its accessory right-arm region. The Abi group of proteins are involved in bacteriophage resistance mediated by abortive infection in *Lactococcus* species. Two 15-bp imperfect DRs (*attL* and *attR*) with consensus sequence 5′-[A/C]GG[A/T]GGAACGTTTGG-3′ were identified at the extremities of the phage integration core site (Fig. 6A).

C2865-pp3 was 126,192 bp and consisted of 166 CDSs, of which only 34 (20.5%) had predicted functions. C2865-pp3 had a GC content of 30.8% and was integrated truncating a *yeeE/yedE* gene, coding for an inner membrane protein, positioned 819,965 bp downstream of *dnaA*. C2865-pp3 genome was organized into three proposed modules delimited by three different integrases, one LSR and two Y-Int/Rec. The combined modules contained genes involved in lysogeny, DNA replication and metabolism, virion packaging, phage morphogenesis, cell lysis, and adaptation (Fig. 7A; see Text S1 for details). Two putative elements involved in phage adaption to the host were detected at its left and central regions, *hicA-hicB* and *ardA*, respectively. The putative HicA and HicB belong to type II toxin-antitoxin systems, where the toxin (HicA) acts as mRNA interferase and the antitoxin (HicB) as neutralizer. The *ardA* gene coded for a putative antirestriction protein ArdA with an N-terminal domain (PF07275) involved in evasion of the bacterial type I restriction-modification system. Moreover, C2865-pp3 harbored a tRNA, which shared 86.7% nucleotide identity with the corresponding tRNA of several *Listeria* phages (see supplemental material for analysis of tRNA codon and amino acid usage; Fig. S9). The integration of C2865-pp3 generated two 8-bp perfect DRs (5′-GTACTTGG-3′) at its boundaries (Fig. 7A). C2865-pp3 shared the closest identity to the prophage-like element of mouse Staphylococcus lentus HT5 and to Staphylococcus phage SPbeta-like, obtained from a clinical Staphylococcus epidermidis 36-1, both carrying three different integrases (Fig. 7B; Text S1). Importantly, the left-arm region of SPbeta-like phage enclosed a composite transposon-like element (IS*257*-flanked) carrying the TMP resistance gene *dfrA* and transposon Tn*4001* (IS*256*-*aacA/aphD*-IS*256*), which harbors the aminoglycoside resistance gene *aacA-aphD* (Fig. 7A).

10.1128/mSystems.00511-21.10 FIG S9 Comparison of codon usage (A) and amino acid (B) percentage between the three prophages (C2865-pp1, C2865-pp2, C2865-pp3) and C2865 host genome. The scale (in percentage) in the left plots is represented at the upper center of the graph. (A) Left, radar plot of the 64 different amino acid codons (at DNA level) highlighting the tRNA gene present in prophage C2865-pp3 genome. Right, specific abundance (%) of TGG codon (UGG). (B) Left, radar plot of the 20 amino acids highlighting proline (P), as resultant amino acid from tRNA present in C2865-pp3. Right, specific abundance (%) of proline (P). Download FIG S9, EPS file, 0.2 MB. Copyright © 2021 Gómez-Sanz et al. 2021 Gómez-Sanz et al. https://creativecommons.org/licenses/by/4.0/ This content is distributed under the terms of the Creative Commons Attribution 4.0 International license.

Sequencing analyses of potential circular intermediates (CIs), as well as restorage of respective phage chromosomal integration genes, revealed that the three prophages could excise the bacterial genome. Phylogenetic analysis of terminase TerL plus the presence of an HNH endonuclease gene in front of terminase *terS*, involved in DNA packaging of *cos* phages (49), predicted a cohesive end packaging strategy (*cos*) for C2865-pp1 and C2865-pp2 (Text S1; Fig. S8).

10.1128/mSystems.00511-21.9 FIG S8 Circular phylogenetic tree of all available staphylococcal terminase large subunit (TerL). The tree built with the amino acid sequences of all identifiable terminase large subunit (TerL) present in 187 staphylococcal phages deposited in the Viral RefSeq database, TerL of C2865-pp1, C2865-pp2, and C2865-pp3, and TerL of *Enterobacteria* phage lambda, used as outlier. Phage nomenclature: S-phage, which refers to staphylococcal phage, is followed by the phage name, faconcant (refering to concatenated fasta), plus the CDSs number of respective TerL in the phage genome. Branch enclosing the C2865-pp1, C2865-pp2, and C2865-pp3 integrases is bold. C2865-pp1, C2865-pp2, and C2865-pp3 integrases are boxed in green, while phage lambda is boxed in gray. Phages with known packaging mechanism (95), carrying either *cos*-sites (*cos* packaging strategy) or *pac*-sites (headful packaging strategy), are indicated in black and green, respectively. Download FIG S8, PDF file, 1.6 MB. Copyright © 2021 Gómez-Sanz et al. 2021 Gómez-Sanz et al. https://creativecommons.org/licenses/by/4.0/ This content is distributed under the terms of the Creative Commons Attribution 4.0 International license.

### The novel S. sciuri pathogenicity island (SscPIC2865) belongs to the SaPI4 family, and related elements are disclosed to be common among S. sciuri genomes.

**FIG 8:**
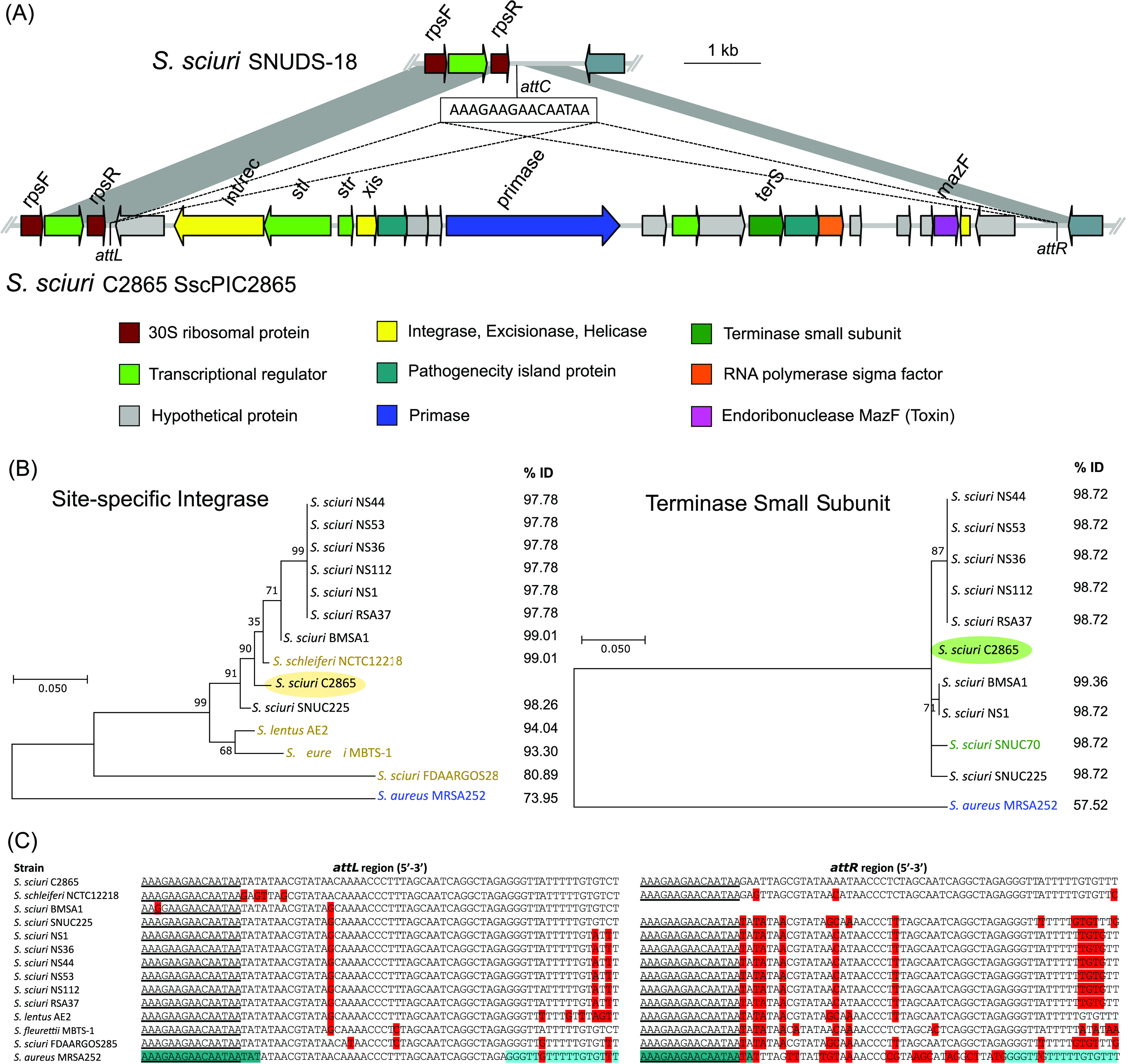
Graphical analysis of the novel staphylococcal pathogenicity island SscPIC2865. (A) Schematic representation of scPIC2865, including its chromosomal integration flanking region, downstream the 30S ribosomal S18 protein (*rpsR* gene), and comparison of the integration region with that of S. sciuri SNUDS-18. Arrows denote the genes, length, and orientation. The core integration site sequence detected (5′-AAAGAAGAACAATAA-3′) as well as position for integration (*attC* for putative integration site in S. sciuri SNUDS-18 chromosome; *attL* and *attR* at both extremities of SsPIC2865) are indicated. Areas of nucleotide similarity (nblastn, >100 bp match, >99% identity) between both structures are indicated in gray. (B) Maximum likelihood phylogenetic tree of (left) all site-specific integrases (Int) deposited in the protein NCBI database and (right) all terminases small subunit (TerS) sharing >80% amino acid identity with respect to those present in SscPIC2865, in addition to those of prototype SaPI4 from S. aureus MRSA252 (69) (displayed in blue). Strains with Int but either lack of or divergent (<50% ID) TerS, and vice versa, are indicated in yellow or green color, respectively. Percentages of amino acid identity with respect to Int or TerS of SscPIC2865 are displayed on the right of the respective tree. TerS-carrying strains corresponded to those harboring the SscPIC2865-conserved integrase in all cases but one (S. sciuri SNUC70), which was integrated at a different position of the bacterial chromosome (at tRNA, *ssrA* gene). (C) Sequence comparison analysis of the phage-related chromosomal island *att* sequence core site region (*attL* and *attR*) of SscPIC2865 and corresponding region of all strains carrying a similar integrase (>80% identity), in addition to that of S. aureus MRSA252 (harboring prototype SaPI4), which also integrates at the 3′-end of the S18 ribosomal gene *rpsR*. Underlined area represents the proposed integration core sequence of SscPIC2865 and related elements. Bases differing from the *att* region of S. sciuri C2865 SscPIC2865 are indicated in red. Original *att* core site length of SaPI4 is indicated in dark blue (69), and the additional direct repeat is shown in blue.

A novel phage-inducible chromosomal island (PICI) of 13,101 bp, named SscPIC2865, was detected 20,705 bp upstream of *dnaA* gene. SscPIC2865 was classified as an S. sciuri pathogenicity island (SscPI), as it contains homologues of the entire core set of genes characteristic of S. aureus pathogenicity islands (SaPIs) and displays synteny with previously characterized SaPIs (Text S1). SscPIC2865 consisted of 21 CDSs, of which 9 (42.9%) lacked any predicted function (Fig. 8A). SscPIC2865 enclosed a tyrosine recombinase (Y-Int/Rec) directing chromosomal integration at the end of the 3′-end of the 30S ribosomal protein S18 gene (*rpsR*). Two SscPIC2865-flanking 15-bp DRs (5′-AAAGAAGAACAATAA-3′) constituted its integrase attachment core sites (Fig. 8A). None of the genes involved in interference of the helper phage reproduction in favor of SscPIC2865 packaging were identified. However, this 3′ region carried a putative transcriptional regulator, an RNA polymerase sigma factor, and a putative helicase, which might be involved in the replication of the element (Fig. 8A). Interestingly, a putative *mazF* toxin gene, coding for an endoribonuclease (mRNA interferase) of a type II toxin-antitoxin system (MazF/MazE), was identified in the accessory region of SscPIc2865.

Several integrases sharing >80% identity to SscPIc2865 integrase were identified, all truncating *rpsR* (Fig. 8B). Of these, nine belong to S. sciuri and the rest to Staphylococcus schleiferi, S. lentus, and Staphylococcus fleurettii. Their integrase-surrounding regions share the characteristic structure of SaPIs (data not shown). We denoted that these elements belong to the SaPI4 family (integrase group I), according to the integrase sequence homology, integration site, and conserved core site 5′-AAAGAAGAACAATAA-3′ (Text S1; Fig. 8C). Sequencing analyses of potential circular intermediates (CIs) and restorage of SscPIC2865 chromosomal integration site revealed that SscPIC2865 could excise the bacterial chromosome.

## DISCUSSION

The combination of Illumina followed by deep PacBio sequencing using large DNA fragmentation and size selection allowed the resolution of the complete genome of IS-rich MDR S. sciuri C2865 at a minimum error rate. This enabled the discovery of transferable TMP resistance *dfrE* within a novel mosaic plasmid and additional novel MGEs. MRSS C2865 revealed high genome plasticity with respect to the S. sciuri genomes analyzed, as it was enriched in novel unique chromosomal and extrachromosomal elements.

We display for the first time that S. sciuri has an open pan-genome, with an ever-increasing flexible genome as new strains are added. Similar patterns were observed in other clinically relevant staphylococcal species formerly analyzed, such as S. aureus (50), S. epidermidis (51–53), Staphylococcus haemolyticus (54), Staphylococcus capitis, and Staphylococcus caprae (52). This result could be anticipated, as an open pan-genome is characteristic of species living in multiple environments and/or mixed bacterial communities. This feature facilitates multiple ways of exchanging genetic material, enabling them to unceasingly expand their total gene repertoire. Our data set revealed MRSS C2865 as the major acceptor of adaptive mobile traits, as it exhibited the highest number of unique genes, most of them (75%) corresponding to MGEs. However, this was not correlated with phylogeny, as C2865 closest relatives (SNUDS-18 and Z8) did not disclose this sharp profile. Based on a range of phenotypic, biochemical, physiological, and genetic analyses, Svec et al. (55) recently showed high S. sciuri intraspecies heterogeneity with no clear differentiation into different subspecies. While agreeing with those statements, our whole-genome-based phylogenomic analysis revealed a clear distinction of an intraspecies subcluster, which included MRSS C2865 (96% ANI with the rest of the S. sciuri genomes). This ANI value falls just above the threshold to be considered a different species (≈95%) (56, 57). Hence, we suggest that there indeed may be subspecies discrimination among the S. sciuri species, but such distinction needs to be addressed by WGS comparisons and might not correspond to the outcomes retrieved by the above-mentioned traditional methods. Alternatively, the available metadata of compared genomes evidence the absence of clear phylogeny delineation depending on the origin, source, or host. This corroborates the low host tropism suggested for this species (3).

We reveal that the novel *dfrE* confers high-level resistance to TMP in both staphylococci and E. coli. DfrE is phylogenetically distant from all staphylococcal TMP resistance genes and displays a common ancestor with Dfrs from soil-associated *P. anaericanus*. Scarce data are available on *P. anaericanus*, but bacteria belonging to this genus are ubiquitous in nature, and closest species have been reported in different environmental sources, such as soils and rhizosphere from different crops (58–60). Importantly, *dfrE* evaded discovery in all available *dfrE*-carrying genomes except *Exiguobacterium* sp. (*dfr_like*), where it proved to confer TMP resistance in E. coli. *Exiguobacterium* spp. are extremophiles adapted to a wide range of habitats, including cold environments (40, 61–64). We also identify here that *dfrE* is already distributed in Asia, as it is present in at least three different staphylococcal species (S. sciuri, S. aureus, and *S. arlettae*) of human and animal origin (including clinical samples) as well as in animal-associated *M. caseolyticus* strain JCSC5402 (65) from several countries. Importantly, all *dfrE*-carrying genomes harbored it in MDR plasmids or plasmid-associated elements. This colocalization is of concern, because it enables the transfer of diverse adaptive traits via a single HGT event. Our data reflect the transferability of TMP resistance via *dfrE* across diverse bacteria from different taxonomic families (order *Bacillales*), diverse environments, and geographically distant regions. Of note, in *Exiguobacterium* sp. strain S3-2, *dfrE* was enclosed within a conserved Tn*3*-like transposon (40). MRSS C2865 (pUR2865-34) and all additional *dfrE*-carrying strains harbored a truncated version of this element. In particular, the *dfrE*-carrying region of S. sciuri GN5-1 (pSS-04) seems to have evolved afterwards by losing an additional recombinase-carrying segment from the Tn*3*-like remnant. This indicates that *Exiguobacterium* sp. harbored an ancestral *dfrE* transposable element that has recombined and jumped to different species from a single ancestor. The fact that the *dfrE* element is already detected in several unrelated animal S. sciuri strains from China and Nigeria, and that S. sciuri GN5-1 has an evolved *dfrE* segment, points toward the assumption that S. sciuri is the ancestral species within the genus, as several recombination events have already occurred within this region. Unfortunately, no flanking regions of the *dfr* gene of *P. anaericanus* are available, so potential homology with its surrounding area could not be addressed. The backbone of mosaic pUR2865-34 denoted a theta replication mechanism, characteristic of larger staphylococcal plasmids. The putative type Ib partitioning system detected is well studied in Gram-negative bacteria, but little to nothing is known about its functions in Gram-positive cocci. These systems contribute to the prevalence and spread of these plasmids, ensuring stable inheritance and effectively maintaining resistance in the absence of selection. pUR2865-34 could only move by conjugation via the detected *oriT* mimic sequence in the presence of a pWBG749-like plasmid or via the integrated *mob* gene and associated *oriT* of pUR2865-int in the presence of a conjugative plasmid (42). However, MRSS C2865 did not harbor any member of the three known staphylococcal conjugative plasmid families (pSK41, pWBG4, and pWBG749) or any recognizable transfer gene cluster that could act in *trans.*

We identified a novel *ccr*-lacking SCC*mec*_C2865_ encompassing additional AMR genes, including an IS*1216E*-flanked region carrying the tetracycline resistance gene *tet*(S). This gene has been found among *Firmicutes* and *Gammaproteobacteria* from diverse ecological sources since the 1950s (66). However, the *tet*(S) has been detected only twice before in staphylococci: (i) among methicillin-resistant Staphylococcus aureus (MRSA) isolates from animal carcasses (67) and (ii) within the S. sciuri SCC*mec*_C2865_-related SCC*mec*TG24 cassette, from ready-to-eat meat (45). The additional closest cassette corresponded to *mecA-mecC* hybrid SCC*mec*-*mecC* in S. sciuri GVGS2 from a bovine infection (20). These strain sources highlight that S. sciuri isolates from animals behave as reservoirs for mosaic MGEs carrying AMR genes.

MRSS C2865 resulted in a polylysogen with three novel unrelated prophages, two of them harboring several putative adaptive features that may promote bacteria and/or phage survival. Only these prophages constituted over 7% of MRSS C2865 genome, evidencing their role as drivers of bacterial evolution and genome modulation. As recently observed by Oliveira et al. (68), a low rate of staphylococcal phages may exhibit more than one site-specific recombinase, as was the case for phage C2865-pp3. Phage integrases are required for the establishment of the lysogeny, but their maintenance is also dependent on the regulatory proteins next to them (68). At least two of its three integrases were surrounded by transcriptional regulators and/or antirepressor proteins. This may enable the integration at additional chromosomal sites depending on the integrase used. In fact, the closest putative prophage, integrated into S. lentus HT5, was incorporated at a different location. Importantly, C2865-pp3-related S. epidermidis SPbeta-like phage harbored an AMR gene cluster enclosed within a composite transposon-like element, harboring resistance determinants for aminoglycosides and TMP. This is a central feature demonstrating that C2865-pp3-related functional phages can indeed harbor mobilizable AMR genes.

PICIs are characterized by a specific set of phage-related functions that enable them to hijack the phage lytic reproduction cycle of helper phages for their own highly efficient transduction. These elements often carry critical staphylococcal virulence genes (69). Several staphylococcal strains harbored SscPIC2865-related PICIs at the same integration site, disclosing that these elements are common among S. sciuri and contribute to genome plasticity and potential toxigenicity. Hence, the ribosomal protein S18 (*rpsR* gene) appears as a hub for the integration of mobile islands and should be considered when addressing staphylococcal chromosomal-integrated elements.

The conservation of the integrase-generated DRs flanking the three prophage genomes, together with their ability to excise the bacterial chromosomal DNA and circularize, as well as the high genome synteny observed among functional phages, strongly suggest that the novel prophages are functional. If this is the case, along with progeny generation, also plasmids or chromosomal DNA of the bacterial host may be mistakenly encapsidated (26). Hence, it is conceivable that any of these prophages may package and mobilize the MGEs discovered here, highlighting any of the three nonconjugative plasmids, the novel *ccr*-lacking SCC*mec*, and/or SscPIC2865 of the SaPI4 family. In fact, PICIs of the SaPI4 family are only induced by endogenous prophages (69). Upcoming studies are warranted to explore the transduction ability of these prophages.

We unveil a transferable TMP resistance gene that has so far evaded identification; although *dfrE* is already present in diverse species from varied sources geographically distant, *dfrE* seems to have emerged from a single ancestor. Molecular data analysis suggests S. sciuri as the donor for transfer within staphylococci. Since *dfrE* seems not yet common in staphylococcal clinical specimens, the data presented here enable early surveillance and facilitate molecular diagnosis, which could promote spread mitigation. Additional experimental analyses may elucidate the spread vehicle and routes for this evolving element. We highlight *S. sciuri* as a resourceful hub for diverse mobilizable adaptive traits.

## MATERIALS AND METHODS

### Bacterial strain selection and characteristics.

MRSS strains C2865, C2853, C2854, and C2855 were obtained in a former study on the occurrence of methicillin-resistant coagulase negative staphylococci from canine samples and were recovered from the groin area of unrelated stranded dogs in Nsukka, Nigeria (12). These strains exhibited TMP resistance, while none of the known staphylococcal TMP resistance genes (*dfrS1*, *dfrD*, *dfrG*, *dfrK*) (32, 33, 35, 36) nor the typical streptococcal/enterococcal *dfrF* gene (34, 37) was detected. All strains were MDR and exhibited related AMR patterns. Strain C2865 was selected for WGS to unveil the genetic basis for TMP resistance. MRSS C2865 was chosen for WGS as it harbored the highest number of detected AMR genes.

### DNA extraction, whole-genome sequencing, assembly, and annotation.

Detailed DNA extraction procedures are described in the supplemental material. Briefly, genomic DNA was extracted by two different methods: for Illumina sequencing, the Wizard Genomic DNA purification kit was used including both lysozyme and lysostaphin (10 mg/ml each) (A1120, Promega Corporation, Spain), and for PacBio sequencing, DNA was isolated using a phenol-chloroform method with some modifications for improved cell lysis. Plasmid DNA was obtained using the GenElute Plasmid Miniprep kit (PLN350, Sigma) also including a lysis step with lysozyme (2.5 mg/ml) and lysostaphin (0.25 mg/ml) for 20 min at 37°C after the resuspension solution step.

High-throughput WGS of S. sciuri C2865 DNA was performed with Illumina Miseq (2 × 300 bp), with NEBNext Ultra kit. For long-read sequencing, PacBio RSII was used after DNA fragmentation of 15 kb followed by a mild size selection. Illumina read quality was checked by Fastqc (103), and reads were trimmed using Trimmomatic v0.36 (70). Good-quality Illumina reads were *de novo* assembled using SPAdes (71). PacBio RSII raw reads were assembled using Canu (72). Protein-coding genes, tRNAs, and rRNA operons were predicted using Prodigal (73), tRNAscan-SE, and RNAmmer on both data sets (74). Predicted protein sequences were compared against the NCBI NR database (NCBI nonredundant database) using DIAMOND (75) and against COG (76) and TIGFRAM (77) using HMMscan (78) for taxonomic and functional annotation. Genomic alignment dot plots between Illumina- and PacBio-resultant contigs were generated with D-GENIES software to evaluate consistency and reliability of both sequencing and assembly approaches (79). Amino acid and codon usages were determined for C2865 chromosome and prophages using the compareM package (https://github.com/dparks1134/CompareM).

### Pan-genome examination, S. sciuri phylogenomic analysis, and comparative genomics.

All genomes available belonging to the S. sciuri group species were downloaded from the NCBI database (accessed in April 2018). In total, 30 strains (29 from NCBI, MRSS C2865) were included, which belonged to the following species (no. of strains): S. sciuri (21), S. lentus (5), Staphylococcus vitulinus (2), S. fleuretti (1), and Staphylococcus stepanovicii (1) (see the supplemental material for strain characteristics). In order to measure the probability of two genomes belonging to the same species, an ANI of the 30 S. sciuri group genomes was calculated using JSpecies as indicated before (56). A heat map was generated using the ANI matrix output table with R (80). In parallel, a maximum likelihood tree for all the S. sciuri group genomes was generated using RAxML (version 7.2.6) (81) using core alignment obtained with Parsnp software within Harvest Suite package (82). Before phylogenomic analysis, all the genomic regions where recombination was detected were removed from the alignment. These regions were determined using the Gingr software, also included in the Harvest Suite package (82), which uses as input the output obtained directly from the Pasnp software. The results were visualized using iTOL v6 (https://itol.embl.de/). All S. sciuri group species other than S. sciuri were considered an outgroup for graphical representation. BLAST Ring Image Generator (BRIG) was used to evaluate and visualize comparisons between MRSS strain C2865 and its closest genomes (>98% ANI), using C2865 as reference (83). Pan-genome analysis (core plus accessory genome) for the 21 S. sciuri genomes was carried out using Roary with a 95% identity cutoff value (84).

### Detection and analysis of resistome and mobilome from S. sciuri C2865.

**Antimicrobial resistance (AMR) genes.** AMR genes formerly detected in MRSS C2865 (see Table S1 in reference 85) were blasted against the WGS of strain C2865. Contig(s) were manually checked for redundant *dhfr* genes and for additional resistance genes of interest.

**Plasmids.** Plasmid contig identification and plasmid reconstruction were achieved by contig coverage, sequence similarity with plasmid backbone genes, gene composition and organization, and contig boundaries redundancy (circularity). Putative *oriT* and *oriT* mimics on the mobilizable elements were searched using the core sequence of those from conjugative plasmids (86). For RCR plasmids, *dso* and *sso* regions, involved in the initiation of replication of the leading and lagging strand, respectively, were searched using core sequences of an RCR representative per plasmid family (87). The secondary structures of *dso*, *sso*, *oriT*, and *oriT* mimic were generated using Mfold web server for single-stranded linear DNA at default parameters (88).

**Prophages.** Detailed analyses are described in the supplemental material. Manual inspection of phage-associated genes (morphology/structure, lysogeny, cell lysis, DNA metabolism) and characteristic functional modular organization was implemented for phage confirmation and integrity. Integrase motif and domain analysis of the translated candidate CDSs was performed against ScanProsite database (89), Pfam database (90), and NCBI conserved domain database (CDD) (91). Integrase-directed generation of DRs as a result of genome integration was investigated manually by sequence comparison of bacterial chromosome-prophage boundaries using a S. sciuri strain prototype (SNUDS-18, GenBank accession no. CP020377) lacking those prophages. ANI pairwise comparison between C2865-enclosed prophages and all staphylococcal phage genomes available in the Viral RefSeq database (accessed until November 2018, *n* = 187) were calculated using the JSpecies with default parameters (56). A heat map was generated using the ANI matrix output table with R (80).

**Staphylococcal chromosomal cassette *mecA* (SCC*mec*).** ORFs found downstream of the integration gene 23S rRNA [pseudouridine(1915)-N(3)]-methyltransferase RlmH, initially known as OrfX, as well as regions containing characteristic SCC*mec* genes (*mecA*, *mecR*, *mecI*, *ccr*) were analyzed. To detect *ccr* gene(s), whose resultant proteins belong to the large S-rec (LSR) family, consensus motif Y-[LIVAC]-R-[VA]-S-[ST]-x(2)-Q derived from Prosite entry PS00397 (http://prosite.expasy.org) was used (supplemental material). Additional S-rec encompassing the Y-[LIVAC]-R-[VA]-S-[ST]-x(4)-Q motif were likewise screened along the entire MRSS C2865 genome. Putative ISS for SCC*mec* or *att* core sites recognized by the typical staphylococcal Ccr were manually identified by searching for the consensus sequence 5′-GAAGC[AG]TATCA[TC]AAAT[AG]A-3′ (supplemental material).

**Others**. Additional recombinases associated with MGEs were investigated as described above for motif and domain analysis (supplemental material). SscPI chromosomal integration site was compared with similar integrase-carrying genomic islands (supplemental material). Integrase-directed generation of DRs as a result of SscPI integration was analyzed by sequence comparison of bacterial chromosome-SscPI boundaries with corresponding regions of S. sciuri strain SNUDS-18, which lacks any insertion in that region. In addition, ISsaga2 web tool was used for IS identification and quantification (92).

**Excision ability of chromosomally located MGEs.** Potential excision and circularization of selected chromosomally located mobile elements, in addition to detection of the resultant chromosomal region after excision, were tested by specific inverse and conventional PCR, respectively (supplemental material; see Table S1 in reference 85).

### Phylogenetic analyses of proteins of interest.

Phylogenetic analyses for Dhfr/Dfr and Ccr of interest were investigated by the construction of unrooted phylogenetic networks with SplitsTree v4 (93) using a neighbor-net with default parameters. Evidence for phylogenetic heterogeneity due to recombination was conducted with SplitsTree v4 using the Phi test for recombination option.

A maximum likelihood phylogenetic tree for MRSS C2865 prophage integrases and all related integrases was built upon amino acid sequence alignment as indicated above (using CLC Genomics Workbench 11) (supplemental material). To estimate the packaging mechanism of identified prophages, a circular phylogenetic tree of the terminase large subunit (TerL) from MRSS C2865 prophages and those identified in the 187 staphylococcal phages deposited in the Viral RefSeq database was likewise created upon amino acid sequence alignment using CLUSTALW (CLC Genomics Workbench 11). TerL of staphylococcal phages with known packaging mechanism was indicated (95).

Phylogenetic analyses for all site-specific tyrosine recombinases and terminases small subunit (TerS) related to those of SscPI in C2865 were performed by the construction of a maximum likelihood tree using the MEGA 7.0.21 program (supplemental material) (94).

### Construction of recombinant plasmids to analyze DfrE functionality, transfer assays, and analysis of native plasmid integrity.

To address the functionality of the candidate TMP resistance gene and for potential synergistic activity by its immediate 3′-end thymidylate synthase gene (*thy*), three different *dfrE*-containing regions were amplified and cloned into pBUS-Pcap-HC (constitutive promoter) or pBUS-HC vectors using the Gibson assembly workflow: (i) *dfrE* gene alone, (ii) *dfrE* gene plus flanking regions, and (iii) *dfrE* gene, *thy* gene, and flanking regions (Table S2; supplemental material) (96). Constructs of interest were transformed into Escherichia coli DH5α and subsequently into S. aureus RN4220 (supplemental material). In addition, native *dfrE*-containing plasmid was transformed into RN4220.

Purified DNA of two independent transformants containing the native *dfrE*-carrying plasmid (RN4220/pUR2865-34) were sent for Illumina WGS (HiSeq, 10 million reads, 2 × 150 bp) for confirmation of the integrity of transformed plasmid. In parallel, one microgram of *dfrE*-containing plasmid DNA from selected transformants (RN4220/pUR2865-34) and from original strains (C2865, C2853, C2854, C2855) was digested with 1 unit of S1 enzyme (1 min at 37°C) (Thermo Fisher Scientific) for plasmid linearization. Plasmid length and integrity were then assayed by gel electrophoresis.

### Antimicrobial susceptibility testing.

Minimum inhibitory concentration of TMP was determined in strains of interest by the agar dilution method on Mueller-Hinton plates (MH, Becton, Dickinson) with a concentration range of 0.5 to 4,096 μg/ml (supplemental material) (97). The agar disk-diffusion method was also used to test for AMR profile of transformants (97). Antimicrobials tested were as follows (μg/disk): erythromycin (15), clindamycin (2), gentamicin (10), kanamycin (30), streptomycin (10 U), tobramycin (10), tetracycline (30), trimethoprim (5), sulfonamide (300), and TMP-sulfamethoxazole (1.25 + 23.75).

### Phenotypic characterization of biofilm formation.

Biofilm formation ability of the four S. sciuri strains as well as S. aureus RN4220/pUR2865-34 transformants was tested by a modified Congo red agar (CRAmod) assay and by crystal violet microtiter plate assay (see supplemental material for detailed procedures) (98, 99). S. aureus SA113 and S. aureus ATCC 25923 were used as positive controls for their strong biofilm-forming potential, while S. aureus RN4220 (DSM 26309) was selected as negative control for biofilm production.

### PCR detection of the TMP resistance gene *dfrE* and the *ica*-locus variant.

Primers were designed for the detection of the TMP resistance *dfrE* gene, the biofilm formation *ica*-locus (*ica*ADBC) variant genes, and the *ica*-locus repressor (*icaR*) (see Table S1 in reference 85). For the *dfrE* gene, primers designed enclosed the Dhfr superfamily domain region (Cd-Search PF00186). All *dhfr* genes with ≥45% nucleotide similarity were included to search for specificity of *dfrE* gene primer set. Original strains (C2853, C2854, C2855), RN4220/pUR2865-34 transformants, and several control strains were tested for sensitivity and specificity (supplemental material).

### Data availability.

The whole-genome shotgun sequence data supporting this work are available via NCBI SRA database under BioProject and Biosample accession numbers PRJNA663854 and SAMN16182282, respectively.
