# Supplementary material for: The Resistome and Mobilome of Multidrug-Resistant Staphylococcus sciuri C2865 Unveil a Transferable Trimethoprim Resistance Gene, Designated dfrE, Spread Unnoticed
Source: mSystems. 2021 Aug 10;6(4):e00511-21. doi: 10.1128/mSystems.00511-21 (PMC8407400; doi:10.1128/mSystems.00511-21)
Supplement: TEXT S1 [file msystems.00511-21-s0001.pdf]

The resistome and mobilome of multidrug resistance *Staphylococcus sciuri* C2865 unveils a transferable trimethoprim resistance gene, designated *dfrE*, spread unnoticed

Elena Gómez-Sanz[1,2*], Jose Manuel Haro-Moreno[3], Slade O. Jensen[4,5], Juan J. Roda-García[3], Mario López-Pérez[3]

[1] *Institute of Food Nutrition and Health, ETHZ, Zurich, Switzerland*

[2] *Área de Microbiología Molecular, Centro de Investigación Biomédica de La Rioja (CIBIR), Logroño, Spain*

[3] *Evolutionary Genomics Group, División de Microbiología, Universidad Miguel Hernández, Apartado 18, San Juan 03550, Alicante, Spain*

[4] *Infectious Diseases and Microbiology, School of Medicine, Western Sydney University, Sydney, New South Wales, Australia.*

[5] *Antimicrobial Resistance and Mobile Elements Group, Ingham Institute for Applied Medical Research, Sydney, New South Wales, Australia.*

[*] Corresponding author: Email: elena.gomez@hest.ethz.ch (EGS)

Short title: Novel *dfrE* and mobile elements in distinct *S. sciuri*

Additional results

Sequencing approach reasoning and general characteristics of *S. sciuri* C2865 genome

Based on the resultant genome-genome alignments using dot-plots of assembled Illumina contigs against the PacBio contigs (reference), 1.9% of the PacBio assembled genome showed no alignment (Fig. S1). Instead, when using the Illumina data as reference, 3.2% of Illumina contigs had no matching (Fig. S1). These unaligned Illumina segments were associated with (i) both small plasmids, (ii) one contig corresponding to plausible contamination and (iii) 166 contigs smaller than 800 bp, many carrying truncated ISs (summing up to 3.1% of Illumina genome). Subsequently, all downstream analyses, except for the two small Illumina-sequenced plasmids, made use of the PacBio data.

A total of 78 ISs were predicted throughout the chromosome of *S. sciuri* strain C2865 using PacBio (data not shown). Among them, 18 were different and belonged to the following families (number of different ISs/total ISs): IS*6* (2/4), IS*200*_IS*605*_ssgr_IS*200* (1/3), IS*256* (2/11), IS*NCY* (2/2), IS*3*_ssgr_IS*150* (6/29), IS*1182* (1/21), IS*21* (1/4), IS*3* (2/2) and IS*110* (1/2). Remarkably, only one IS was validated by the system, due to low percentage of identity with deposited ISs in ISfinder (https://isfinder.biotoul.fr/) implying that most of the identified ISs were new. The plasmid retrieved by PacBio predicted and validated five ISs (two different belonging to IS*6* family). Illumina data produced 22 predicted ISs (15 different) (Table 1), most likely due to the collapse of the repeated regions within the ISs.

Significant differences were observed in the total number of pan-genome genes detected by MRSS C2865 PacBio and Illumina data (5,721/5,220, p-value=1.7E-06). These differences were

associated with the accessory genome (4,174/3,584, p-value=2.1E-11) and unique genes (1,996/1,722, p-value=7.0E-06).

Chromosomal *radC* integration of cadmium resistance ψTn*554*

A variant of the MLS_B and spectinomycin resistance unit transposon Tn*554*, known as ψTn*554*, was identified in MRSS C2865 integrated in the chromosome truncating the DNA repair *radC* gene at its 3' region, 1'316'939 bp downstream of *dnaA* gene (Fig. S3). This ψTn*554* was 7'306 bp and belonged to the Tn*554* family of unit transposons, which insert at high efficiency into a primary unique site in the staphylococcal chromosomes (*att554*, within the *radC* gene) (1). Transposon ψTn*554* contains a cadmium resistance gene cluster whose products code for a predicted transcriptional regulator (CadC), a cadmium-translocating P-type ATPase (CadA) and a cadmium resistance transporter family protein (CadD). ψTn*554* shared highest identity (94% coverage, 97.5% identity) with a *radC*-independent chromosomal region detected in a highly cytotoxic and clinically virulent *S. aureus* strain 6850 (2) (Fig. S3). Within *S. sciuri* available genomes, only a truncated version of this ψTn*554* was present within SCC*mec*GVGS2 cassette of *S. sciuri* strain GVGS2 (Fig. 5), representing the single former report of a ψTn*554*-like structure in this species (3). Nine additional *S. sciuri* genomes revealed to carry interrupted *radC* copies or 3'-end *radC* remnants (data not shown). In all these cases, the *radC* gene was intruded by a mobile element carrying the recently described oxazolidinone and phenicol resistance gene *optrA* (Fig. S3).

An additional unique region of ca. 11.5 kb was detected 390,146 bp downstream of *dnaA* (Fig. 1B). This segment carried an NgoFVII family restriction endonuclease cluster (restriction modification system of the Type II), which was delimited at its downstream boundary by a transposase [N-terminal domain PF05598) and C-terminal domain DDE_Tnp_1_6 (PF13751) of

the DDE superfamily]. This cluster included a Type II restriction endonuclease of the NgoFVII family (PF09562), which carries out the endonucleolytic cleavage of DNA to give specific double-stranded fragments with terminal 5'-phosphates (by recognizing the double-stranded sequence GCSG/C), and two consecutive DNA methyltransferases (DNA MTase) (PF00145).

Potential genes or chromosomal elements involved in conjugation, such as integrative or conjugative elements or conjugative transposons, which could mediate intercellular transfer of identified MGEs, were not detected.

Small single resistance rolling-circle replicating pUR2865-1 and pUR2865-2 plasmids

Small plasmids pUR2865-1 (2,559 bp) and pUR2865-2 (3,830 bp), which carried the *lnuA* gene, encoding the lincosamide nucleotidyltransferase LnuA, and the cat_{pC221} gene, encoding the chloramphenicol acetyltransferase Cat_{pC221}, respectively, were detected (Fig. S4). Both plasmids consist of a basic plasmid backbone, with a replication initiation protein (Rep), as well as a mobilization initiation protein or relaxase (Mob) in the case of pUR2865-2. Here, an *oriT* was detected in the immediate upstream region of *mob*. Both *dso* and *sso* were detected in both plasmids based on sequence homology and secondary structure analysis (Fig. S4B-C).

Based on the *dso* region, amino acid identity and motif analysis of Rep with one prototype plasmid per RCR family (pC194, pC221, pT181, pE194, pPV141, pSN2 and pUB110) (1), the novel plasmid pUR2865-1 belonged to the pC194 family (Fig. S4B). Even though Rep identity to prototype pC194 was substantially low (Rep 24.1% ID), all pC194-family conserved motifs were present in pUR2865-1, highlighting the HUH motif (U-bulky hydrophobic residue; here Met178), whose His residues are involved in metal ion coordination required for the activity of RCR, and the HUH C-proximal Glu213 and Tyr217, corresponding to both catalytic residues (4). The

putative *dso nick* site was found immediately upstream of the *lnu*(A) gene and contained the following sequence: 5'-TCTT-CTTgTCTTG/ATAcTA-3' (capital letters denote conserved bases with respect to that pC194, and slash the site of cleavage) (5). Plasmid pUR2865-2 shared clear conserved regions with the pT181 family (Rep 69.4% ID) (Fig. S4C). The *dso* was located within the N-terminal region of the *repC* gene and the conserved *nic*k site was identified: 5'-AAAACCGGCgTACTCT/AATAGCCGGTT-3' (5, 6) (Fig. S4). Characteristic *ssoA*-enclosed conserved recombination site (RS_B) 5'-GAGAAAA-3' and the primer RNA transcriptional terminator CS-6 (5'-TAGCGT-3'), needed for replication initiation of the lagging strand, were detected upstream of *lnu*(A) and downstream of the relaxase gene, in pUR2865-1 and pUR2865-2, respectively (Fig. S4).

Comparative analysis of pUR2865-1 with closest publicly available plasmids is shown in Fig. S4D. Such plasmids were present in different staphylococcal species of diverse origins (human and dairy cattle clinical samples) and are as follows (GenBank accession no.): *S. aureus* strain C5425 plasmid pUR5425, complete sequence (JQ861958.1); *S. aureus* strain SR434 plasmid pSR04, complete genome (CP019567.1); *Staphylococcus chromogenes* plasmid pLNU4, isolate KNS48 (NC_007771.1); *Staphylococcus simulans* plasmid pLNU2, isolate 184/61 (NC_007769.1). As for pUR2865-2, closest relatives shared 70-78% coverage and ≥ 81% nucleotide ID, and they were mainly enclosed in *S. aureus* of different origins (Fig. S4E). Represented samples are as follows (GenBank accession no.): *Staphylococcus hyicus* strain 1211 plasmid pSWS1211 (KM276081.1); *S. aureus* plasmid pSWS2889, strain MRSA ST398, isolate 1110902889 (NC_023385.1); *S. aureus* plasmid pDJ91S, complete sequence (KC895984.1); *S. aureus* chloramphenicol resistance plasmid pKH7, complete sequence (U38429.1); *S. aureus* plasmid pOC160-2 DNA, complete sequence, strain OC160 (LC012933.1); and *S. aureus* R-

plasmid pSBK203 replication initiation protein gene, chloramphenicol acetyltransferase gene, and Pre protein gene (U35036.1).

Mosaic mobile adaptive elements enclosed within the novel plasmid pUR2865-34

Illumina output plus scaffolding generated two potential plasmids (pUR2865-3 and pUR2865-4) flanked by IS*257* elements, which combination corresponded to PacBio sequenced pUR2865-34 (Table 1). Plasmid pUR2865-34 length and integrity was confirmed by plasmid linearization and gel electrophoresis in addition to WGS analysis of two *S. aureus* RN4220 transformants (RN4220/pUR2865-34) (data not shown).

Four identical copies of staphylococcal IS*257* in the same orientation (with their 15-bp flanking perfect inverted repeats [IRs] 5'-GGTTCTGTTGCAAAG-3') and one copy of the enterococcal IS*1216E* (with 23-bp flaking perfect terminal IRs 5'-GGTTCTGTTGCAAAGTTTTAAAT-3') were identified.

pUR2865-int shared 65.1% and 47.2% nucleotide identity to RCR prototype pT181 and pUR2865-2 (RepC 76.9% ID), respectively. This integrate was flanked by two identical copies of IS*257* in the same orientation. Two identical DRs on the internal ends (to the insertion) for each IS copy were detected, suggesting that this IS*257* set may have mediated insertion of a pT181-like ancestor carrying one IS copy with an IS*257*-carrying pUR2865-34 precursor by homologous recombination. Of note, the IS*257* copy adjacent to the *rep* gene in pUR2865-int has disrupted its promoter region (data not shown), rendering this gene likely not functional.

Three S-rec genes of the resolvase/invertase subfamily, named *res/rec* (*res/rec1, res/rec2, res/rec3*), were identified. They contained the characteristic catalytic N-terminal PF00239 domain

(with consensus motif 5'- Y-[AI]-R-V-S-[ST]-x (2)-Q- 3') and the C-terminal helix-turn-helix (HTH) domain of S-rec resolvases (PF02796).

Phylogenetic analysis of DfrE (>50% ID) in NCBI database plus all Dfrs responsible for TMP resistance in staphylococci revealed that DfrE was closer to the Dhfr of soil-related *Paenibacillus anaericanus* (68% ID) (Fig. 4). Comparative analysis of (i) all Dhfr/Dfrs detected in the 30 *S. sciuri* group genomes available, (ii) all TMP resistance Dfrs formerly detected in staphylococci and (iii) two susceptible Dhfrs as reference (Dhfr from *S. epidermidis* ATCC12228 and DhfrB from *S. aureus* ATCC25923) revealed that both chromosomally located Dhfrs cluster with intrinsic, susceptible staphylococcal Dhfrs whereas DfrE shared closer phylogenetic identity with TMP resistance DfrF, typical of enterococci and streptococci (Fig. S5).

Of note, two 6-bp identical DRs (5'-AAAAGC-3') and two 11-bp imperfect DRs (consensus: 5'-CAAAT[C/T]CT[C/A]TT-3'), were detected immediately up- and downstream of the *dfrE*-carrying structure. In addition, two 19-bp perfect IRs (5'-GCACAATAAAAGCAAGAGG-3') located upstream of *ser*/*rec2* were detected, which might be involved in the recombination of this region (Fig. 3B).

The *ica*-locus variant shared 59.6% nucleotide identity with intercellular adhesion *ica*-locus prototype of methicillin-resistant *S. aureus* (MRSA) strain N315 (GenBank ac. No. BA000018) (7, 8). Comparison of the deduced amino acid sequences with MRSA strain N315-encoded Ica proteins revealed identities of 60.9% for the IcaA protein, 35.8% for IcaD, 47.3% for IcaB, 47.2% for IcaD and as low as 26.3% for IcaR. Regardless both IS copies likely mediated movement/capture of this adhesion cluster variant, IS*257*-characteristic 8-bp DRs were not detected.

pUR2865-34 backbone region enclosed a replication initiation *repA* gene with the typical RepA_N N-terminal domain, characteristic of theta-replicating plasmids (1). The N- and C-terminal domains of the encoded protein were homologous to different RepA_N proteins, showing that these proteins can undergo domain replacement events. A Type Ib partitioning system, involved in plasmid segregational stability, consisting of a *parA* gene (coding for a deviant Walker ATPase motor protein) and a *parB* gene (coding for a DNA-binding protein with a RHH secondary structure motif) was detected. Resultant ParA and ParB shared high identity with *Staphylococcus aureus* apramycin resistance plasmid pAFS11 sequence, although the Type-Ib system was not annotated in this plasmid (8). pUR2865-34 harbored a single relaxase gene (*mob*) with an origin of transfer (*oriT*) sequence immediately upstream this gene, which was enclosed within the RCR integrated plasmid pUR2865-int (Fig. 3). In addition, an *oriT* mimic sequence (positions 6,077 to 6,132) similar to one recently described by M. Bukowski et al. (9) was observed proximal to the replication and partitioning genes (Fig. 3A). However, conjugation related genes from known staphylococcal conjugative elements, such as pSK41, or integration and conjugative elements, which could act in *trans* for plasmid transference, were not detected throughout the entire bacterial genome.

Ambiguous biofilm formation ability of *ica*-locus variant containing strains

Based on the CRAmod assay, all four *ica*-locus variant carrying *S. sciuri* strains were biofilm formers, while they were considered weak biofilm formers by the CV assay (Fig. S6). By this later method, both positive controls, strains SA113 and DSM1104, confirmed strong biofilm formers. Instead, remarkable lack of correlation was observed with the non-biofilm former *S. aureus* RN4220 and both RN4220 transformants carrying the pUR2865-34 plasmid (S319 and S320) between both assays (Fig. S6). Based on colorimetry by the CRAmod assay, neither recipient *S.*

aureus RN4220 strain nor resultant transformants were biofilm formers (Fig. S6A). Instead, by the CV assay, both recipient and resultant transformants resulted biofilm formers, with *S. aureus* RN4220 (Fig. S6B). Yet, the *ica*-negative non biofilm producing *S. lentus* strain (C3030) confirmed lack of biofilm formation.

dfrE, *ica*-locus variant and pUR2865-34-like are present in C2853, C2854 and C2855 strains

In addition to *S. sciuri* C2865, the three additional TMP resistant *S. sciuri* strains carried the novel *dfrE* gene and the *ica*-locus variant cluster, based on the PCR assays. Linearized-plasmid analysis by gel electrophoresis revealed that these three strains also harbored a similar-sized plasmid as pUR2865-34. Strains C2853, C2854 and C2855 were also resistant to tetracyclines, macrolides-lincosamides and aminoglycosides and harbored the corresponding *tet*(K), *erm*(B) and *aacA-aphD* resistance genes, which were also present in mosaic plasmid pUR2865-34 (Table S1 in reference (10)).

Novel SCC*mec*$_{C2865}$ element lacking formerly described chromosomal cassette recombinases

Two chromosomally located homologues of the *mecA* gene were detected: the methicillin-resistance determinant *mecA* and an intrinsic copy of the methicillin-susceptible variant *mecA1*. Resultant proteins shared 81.4% identity. The *mecA1* gene of *S. sciuri* C2865 shared 99.7% nucleotide identity (99.4% at amino acid level) to the *mecA1* gene present in methicillin-susceptible *S. sciuri* strain K11 (GenBank acc. No. Y13094), exhibiting five-point substitutions. One of these substitutions implied a stop codon (Glu607Stop) at the 3'-end region of penicillin binding protein transpeptidase domain (PF00905), resulting in a truncated shorter variant of MecA1. Both, *mecA* and *mecA1*, were located on the chromosomal right-arm near the start origin of replication (*dnaA*) (41,464 and 177,170 bp downstream of *dnaA*, respectively).

SCC*mec*$_{\text{C2865}}$ was delimited at both ends by characteristic SCC*mec*-flanking DRs with typical insertion site sequences (ISS) or attachment sites (*att*): *att*R 5'-GAAGCATATCATAAATGA-3' at the 3'-end of *rlmH* and an identical direct repeat (DR) designated *att*L3 at the right boundary of the cassette, defining a transferable unit. Two additional imperfect *att* sites (*att*L1 5'-GAAGCGTATCACAAATAA-3' and *att*L2 5'-GAGCCATATAATAAATAA-3') were detected within SCC*mec*$_{\text{C2865}}$ at base-pair position 29,884 and 41,929 of the cassette, respectively, comprising 6 potential transferable units with the potential to form circularly excised elements: three different SCC*mec* cassettes (segments enclosing the *att*R + either *att*L1, *att*L2 or *att*L3) and three SCC cassettes (*att*L1 + either *att*L2 or *att*L3; *att*L2 + *att*L3). (Fig. 5A). Characteristic imperfect IR sequences, required for CcrAB or CcrC recognition of the *att* sites, were located at the internal boundaries of the complete cassette (*att*$_{\text{IR-R1}}$ 5'-AATGATGCGGTTTTTT-3' at the 3'-end of *att*R and *att*$_{\text{IR-L3}}$ 5'-TAAAAACCGCAACATT-3'located 11 bp upstream *att*L3), as well as at the upstream boundary of *att*L1 (*att*$_{\text{IR-L1}}$ 5'-TAAAAACCGCATCATT-3' 12 bp upstream of *att*L1) and *att*L2 (*att*$_{\text{IR-L2}}$ 5'-ATAAAACCAAACCATT-3' 113 bp upstream of *att*L2) (11). SCC unit within *att*L1-*att*L2 boundaries consisted of a unique 11-kb region enclosing a cluster of genes coding for several metabolic routes. Of note, part of this region (62-66% coverage, >96% ID) was present in the chromosome of *S. xylosus* SA4009, *S. xylosus* S04010 and *S. xylosus* C2a (12), just after the SCC*mec* downstream junction. The *att*L2-*att*L3 module encompassed another copy of the arsenic resistance operon CBR (*arsCBR*) and an additional arsenic resistance gene (*arsAD*) plus an arsenic transcriptional regulator.

Closest LSR to currently described Ccrs was LSR$_{\text{C2865-pp1}}$ (sharing 27.4% ID with CcrC2 and CcrB4), followed by LSR$_{\text{C2865-pp2}}$ (25.2% ID with CcrB4) and LSR$_{\text{SCC}mec}$ (24% identity with

CcrC1). Of note, PCRs for potential CIs of the different units, as well as an intact chromosomal *rlmH* region (after SCC*mec* excision) were negative.

SCC*mec*TXG24 from *S. sciuri* TXG24 (GenBank accession no. KX774481) and SCC*mec*GVGS2 from *S. sciuri* GVGS2 (HG515014) were identified as closest relatives to SCC*mec*$_{C2865}$, sharing 57% and 40% coverage, respectively (3, 13).

Unique *Siphoviridae* prophages vB_SsS-C2865-pp1, vB_SsS-C2865-pp2 and vB_SsS-C2865-pp3 enclose adaptive features and excision and circularization ability

ANI, phage classification and presumed packaging strategy. C2865-pp1 and C2865-pp2 shared highest identity each other (82% ANI; NCBI blastn 33% coverage, 88.3% ID). (Fig. S7). Alternatively, *Staphylococcus* siphophage SPbeta-like shared closest identity with C2865-pp3 (66% ANI; NCBI blastn 46% coverage, 72.91% ID) (Fig. S7) (14). According to a recent reclassification scheme by H. Oliveira et al. (14), based on phage genome homology, genome size and gene synteny, modular organization, percentage of functionally annotated predicted genes, temperate lifestyle and lack of or simple baseplate region, the three prophages were classified as S*iphoviridae*: C2865-pp1 and C2865-pp2 belonged to the staphylococcal phage cluster B, while C2865-pp3 clustered with SPbeta-like Shipophage, constituting the unique singleton so far among staphylococcal phages. Regardless of its large genome (126 kb), phage C2865-pp3 seems to belong to the *Siphoviridae* family as its putative simple base plate region (structural region highly conserved in *Siphoviridae*) shares high structural homology to that of lactococcal temperate *Siphoviridae* phage TP901-1, a model for *Siphoviridae* virion assembly (15).

According to TerL phylogenetic tree, C2865-pp1 and C2865-pp2 TerL clustered with typical *cos*-packaging phages, such as *S. aureus* 2638A phage. Hence, the predicted packaging mechanism

used by these two novel phages seems to be mediated by cohesive ends (16) (Fig. S8). This was correlated in both phages with the presence of an HNH endonuclease gene in front of *terS*, specifically involved in DNA packaging of *cos* phages (17). No packaging mechanism could be proposed for C2865-pp3, as its TerL was allocated distant from *cos/pac* characteristic clusters.

Figure 6 (A) depicts a comparative analysis of C2865-pp1 and C2865-pp2 and its closest relatives based on (i) all staphylococcal phages deposited in the Viral RefSeq database and (ii) the genetic elements enclosing the closest integrases in the NCBI pBLAST database (*S. sciuri* BL01, contig 1 [WGS, RefSeq accession no. NZ_SPPH01000001], *S. sciuri* CCUG39509, contig 76 [NZ_PPRP01000076], *S. sciuri* SNUC1679, contig 10 [NZ_QXVB01000010]). The integrases from C2865-pp1 and C2865-pp2 encompassed the characteristic LSR resolvase, recombinase and the zinc beta ribbon domains (PF00239, PF07508 and PD13408, respectively), and shared the core consensus motif typical of LSR Ccrs from SCC*mec* elements (Y-[LIVAC]-R-[VA]-S-[ST]-x(2)-Q). Figure 6 (B) demarks a maximum likelihood phylogenetic tree of C2865-pp1 and C2865-pp2 integrases, as well as all integrases sharing >80% amino acid identity with respect to either C2865-pp1 and C2865-pp2 integrases (*S. sciuri* NS1, contig 57 [WGS, NCBI accession no. LDTK01000057], *S. sciuri* NS53 ,contig 22 [LDTP01000022], *S. sciuri* CCUG39509, contig 76 [WGS, RefSeq accession no. NZ_PPRP01000076], *S. sciuri* SNUC1679, contig 10 [NZ_QXVB01000010], *S. sciuri* BL01 , contig 1 [NZ_SPPH01000001], *S. sciuri* RSA37, contig 32 [LDTQ01000032], *S. sciuri* NS44, contig 13 [LDTO01000013], *S. sciuri* NS36, contig 30 [LDTN01000030], *S. sciuri* NS112, contig 46 [LDTL01000046]), and integrase of Enterobacteria lambda phage (NC_001416).

C2865-pp1. The LSR integrase gene was detected at the left extremity of the phage (Fig. 6). Two transcriptional regulators of the XRE superfamily (PF01381) were detected in front of $lsr_{\text{C2865-pp1}}$.

These genes are homologs of lambda phage transcriptional regulators *cI* and *cro* (Cro/C1-type HTH domain) responsible for maintenance of phage integration (CI, lysogeny) and excision (Cro, lytic cycle). In addition, a phage antirepresor with two distinct antirepresor domains [N-terminal AntA/AntB family domain (PF08346); C-terminal KilAC domain (ANT family, PF03374)] was identified immediately downstream of the second transcriptional regulator. Comparative analysis of $LSR_{C2865\text{-}pp1}$ on the NCBI protein database revealed a single hit enclosed in *S. sciuri* strain BL01 (NCBI RefSeq. No. NZ_SPPH01000001) (Fig. 6B). This integrase belonged to a putative prophage integrated at the same chromosomal gene as C2865-pp1; however, the DR were not exactly conserved, with consensus sequence 5'-AAT[A/G]GT-3'. At the right-arm end, C2865-pp1 endolysin gene was arranged as a single gene with an N-terminus CHAP domain (PF05257), a central N-acetylmuramoyl-L-alanine amidase domain of the Ami_2 family (PF01510) and a C-terminal domain of the SH3 superfamily (PF08460).

C2865-pp2. The LSR integrase gene, $lsr_{C2865\text{-}pp2}$, was detected at the left extremity of the phage (Fig. 6). Two $lsr_{C2865\text{-}pp1}$-unrelated transcriptional regulators of the XRE superfamily (PF01381) were detected in front of $lsr_{C2865\text{-}pp2}$. The one closer to $\mathrm{lsr}_{C2865\text{-}pp2}$ harbored an additional C-terminal peptidase domain (Peptidase S24-like, PF00717), typical of the lambda repressor CI/C2 family and related bacterial prophage repressor proteins. The putative adaptive gene AbiF shared highest identity (97% coverage, 58.1% ID) to the AbiF of food-associated biofilm-former *Staphylococcus cohnii* MF1844 (RefSeq assembly. No. GCF_001876725), among others. Two 15-bp imperfect DRs (*attL* and *attR*) with consensus sequence 5'-[A/C]GG[A/T]GGAACGTTTGG-3' were identified at the extremities of the phage integration core site, with 5'-aGGaGGAACGTTTGG-3' (*attL*; which remained in the restored chromosomal bacterial gene upon prophage excision: *attB*) and 5'-cGGtGGAACGTTTGG-3' (*attR*, carried in the excised circularized element: *attP*)

allocated at each phage boundary (Fig. 6A). Comparative analysis of $LSR_{C2865\text{-}pp2}$ revealed that eight *S. sciuri* lysogens shared related integrases (Fig. 6B). In all but one case we could determine the integrated elements were incorporated truncating the same chromosomal gene as in MRSS C2865; however, no conservation of the DRs was observed in MRSS C2865 (Fig. 6). At the right-arm end, C2865-pp2 endolysin gene was organized as a single gene with an N-terminus CHAP domain (PF05257), a central N-acetylmuramoyl-L-alanine amidase domain of the Amidase_2 family (PF01510) and a C-terminal domain of the SH3 superfamily (PF08460).

C2865-pp3. Figure 7 (B) depicts a Maximum likelihood phylogenetic tree of the three integrases detected in C2865-pp3, as well as those present in its two closest (pro)phages (NCBI NR search) (Staphylococcus phage SPbeta-like and *Staphylococcus lentus* HT5, contig 1), and closest integrase (>80% amino acid identity, *S. sciuri* SNUC1345, contig 49) and integrase of Enterobacteria lambda phage (NC_001416), as outgroup. Regardless of their lower identity with those of C2865-pp3, their Y-Int fell within the same sub-clusters as those of C2865-pp3 (Fig. 7B). As expected by the low amino acid identity of the recombinase leading integration (40.1%), HT5-prophage-like element was incorporated at a different location, *i.e.* within the intergenic region of an ABC transporter ATP-binding protein and a ABC transporter permease genes, respectively (Fig. 7), both present in the chromosomal DNA of *S. sciuri* C2865.

C2865-pp3 genome was organized into three proposed modules delimited by three different integrases. The leftmost 60.5-kb leftwards-transcribed arm carries genes whose predicted proteins are involved in lysogeny (integrase, DNA-binding protein), DNA metabolism (ribonucleoside reductase, DNA polymerase, deoxyribosyltransferase, methyltransferases, single strand DNA binding, DNA ligase, helicase DnaB) and adaption (Type II toxin-antitoxin system HicA and HicB). A centrally located 34-kb region, half leftwards and half rightwards transcribed, carried

genes involved in lysogeny (integrase, represors, supplementary data), DNA replication (helicase), virion packaging (only TerL identified), and adaption (antirestriction ardA, Pro TGG-tRNA). The 31.5-kb rightmost rightward-transcribed phage genome region encompassed genes involved in phage morphogenesis (tape measure) and cell lysis (holin, endolysin) (Fig. 7A).

The leftmost located integrase gene (*lsr*$_{C2865\text{-}pp3}$), belonged to the S-rec LSR subfamily and was responsible for C2865-pp3 integration within *yeeE/yedE* (Fig. 7) It carried the characteristic N-terminal resolvase and the central LSR recombinase domains. *lsr*$_{C2865\text{-}pp3}$ was preceded by a CDSs enclosing a DNA-binding domain (HTH) of the of the transposase family ISL*3* (PF13542), in the same orientation, which could behave as a transcriptional regulator. The centrally-located integrase, named *Y-int/rec1*$_{C2865\text{-}pp3}$, belonged to the Y-rec family and harbored the typical Y-rec C-terminal catalytic domain (PF00589). This integrase was preceded by an XRE superfamily transcriptional regulator (HTH_3 family, PF01381) as well as by a phage antirepresor with two distinct antirepresor domains [N-terminal Bro-N family domain (PF02498), C-terminal KilAC domain of the ANT family (PF03374)]. Both regulatory genes where disposed in opposite orientation to *Y-int/rec1*$_{C2865\text{-}pp3}$ gene. The third integrase copy, located at the right arm and designated *Y-int/rec2*$_{C2865\text{-}pp3}$, also belonged to the Y-rec family and encompassed a phage integrase N-terminal SAM_4-like domain (PF13495) and the phage integrase family C-terminal catalytic domain (PF00589). However, this integrase gene lacked any identifiable transcriptional regulator at its immediate surroundings. Instead, a CDS coding for a XRE superfamily transcriptional regulator was detected by CD-search (HTH_XRE, cd00093) 19 CDSs after *Y-int/rec2*$_{C2865\text{-}pp3}$, in the same orientation. The LSR$_{C2865\text{-}pp3}$ shared 20.6 and 23.1% identity with LSR of C2865-pp1 and C2865-pp2, respectively, while both tyrosine integrases shared 20.8% identity. LSR$_{C2865\text{-}pp3}$ and tyrosine recombinase Y-Int/Rec2$_{C2865\text{-}pp3}$ were unique in the entire NCBI NR

database (>70% coverage, >80% ID). Instead, two hits were retrieved for tyrosine recombinase Y-Inte/Rec1$_{C2865\text{-}pp3}$, one of them in the genome of *S. lentus* strain HT5 in a similar prophage-like region (Fig. 7B). At the right-arm end, C2865-pp3 endolysin gene was arranged as a single gene with and N-terminus CHAP domain (PF05257) and a centrally located N-acetylmuramoyl-L-alanine amidase domain of the Amidase_3 family (PF01520), but lacked any C-terminal SH3-carrying domain.

Remarkably, an antitoxin *hicB* gene copy of the HicB_lk_antitox superfamily (PF15919) lacking any associated *hicA* was also identified at a different region of the *S. sciuri* C2865 bacterial chromosome (602'665 bp downstream of *dnaA*). C2865-pp3 harbored a transcriptional RNA with a Proline anticodon (Pro TGG tRNA) within the central region (phage position 81,705), which shared 86.7% nucleotide identity with corresponding tRNA of a number of Listeria phages (phage LP-124, LP-083-2, LP-064 and LP-125). The three prophages and the entire MRSS C2865 bacterial genome were evaluated for codon usage and amino acid content to appraise whether acquisition of this tRNA could be an adaptive feature to favor phage replication, consequence of an increase TGG or proline within phage genome. However, proline was less abundant in C2865-pp3 (2.1%) than in MRSS C2865 genome (3.1%) and the other two phages (2.8-3%) (Fig. S9). TGG codon usage was alike in both C2865-pp3 and C2865 genome (0.85% each), while revealed increased in C2865-pp1 and C2865-pp2 (1.18, 1.17%, respectively) (Fig. S9).

C2865-pp3 shared closest identity to prophage-like element of *S. lentus* HT5 (ca. 129 kb) (RefSeq acc. No. NZ_SPOY01000001) and to 127.7-kb *Staphylococcus* phage SPbeta-like, obtained from a clinical *S. epidermidis* 36-1 in Russia (Viral RefSeq acc. No. NC_029119). Of note, both phages also carried three different integrases (Fig. 7).

The novel *S. sciuri* pathogenicity island (SscPIC2865) belongs to the SaPI4 family and related elements disclose common among *S. sciuri* genomes

Immediately upstream of the *Y-int/rec* gene, two divergently oriented CDSs encoding DNA-binding proteins of the HTH_XRE superfamily (CD-search cl22854), termed Stl and Str in elements of the SaPI family, were detected (18). Stl is essential for maintenance of lysogeny while Str is responsible for activation of the lytic cycle prior mobilization by a helper phage. An excisionase gene (*xis*), involved in excision of SaPI-like elements upon regulation of these repressors, was located downstream of *str* gene. In the middle of the island, a primase gene [D5 N-terminal like domain (PF08706), characteristic of DNA viruses and P4 DNA primase phages] fusioned with a replication initiation protein gene [C-terminal domain (PF032288)] was detected. This was coupled with an AT-rich iteron region at its 3'-end, comprising the putative replication origin. Only two hits >80% identity to SscPIC2865 primase were detected: primase of *S. fleurettii* MBTS-1 (96.5% ID, NCBI RefSeq. No. NZ_MWJM01000005) and that of *S. sciuri* SNUC740 (80.6% ID; NZ_PZHE01000007). Downstream of this area, a copy of a terminase small subunit (*terS*) of the family Terminase 2 was detected (Fig. 8A).

Figure 8 (B) depicts a maximum likelihood phylogenetic tree of (left) all site-specific integrases deposited in the protein NCBI database and (right) all terminases small subunit (TerS) sharing >80% amino acid identity with respect to those present in SscPIC2865 (*S. sciuri* NS44 [NCBI RefSeq. No. NZ_LDTO01000002]; *S. sciuri* NS53 [NZ_LDTP01000017]; *S. sciuri* NS36 [NZ_LDTN01000005]; *S. sciuri* NS112 [NZ_LDTL01000018]; *S. sciuri* NS1 [NZ_LDTK01000052]; *S. sciuri* RSA37 [NZ_LDTQ01000004]; *S. aureus* BMSA1 [NZ_LFXB01000008]; *S. sciuri* SNUC225 [NZ_PZHF01000049] for both Int and TerS; *S. schleiferi* NCTC12218 [LR962863]; *S. lentus* AE2 [NZ_SPPP01000007]; *S. fleurettii* MBTS-1

[NZ_MWJM01000005] and *S. sciuri* FDAARGOS285 [NZ_CP022046] for Int; and *S. sciuri* SNUC70 [NZ_PZHH01000006) for TerS), in addition to those of prototype SaPI4 from *S. aureus* MRSA252 (GenBank acc. No. BX571856), the integrase of which belongs to *att*/integrase family I (18) (displayed in blue).

Among the six groups of site-specific integrases detected so far among elements of the SaPI family, SaPIs of integrase group I, with SaPI4 as prototype, also integrate at the 3' region of the ribosomal protein S18 (*rpsR* gene) (18). Even though the amino acid identity of SaPI4 (*S. aureus* MRSA252) and SscPIC2865 integrases was considerably low (74%) (Fig. 8B), they belong to the same SaPI4 family (conserved core site 5'-AAAGAAGAACAATAA-3'). To address whether SscPIC2865 integrase and relatives belong the SaPI4 family, the integration region (*attL*, *attR*) of these elements was analyzed in further detail (Fig. 8C). The SscPIC2865 perfect DR 5'-AAAGAAGAACAATAA-3' (15 bp) was detected at both extremities of the islands in all cases, including in *S. aureus* MRSA252, except for *S. sciuri* strain BMSA1 (Fig. 8C). Hence, we propose SscPIC2865-related elements belong to the same Int group I, with conserved core site 5'-AAAGAAGAACAATAA-3', instead of the slightly longer formerly deduced attachment site of Int group I 5'-AAAGAAGAACAATAATAA-3'.

Sequencing analyses of potential CIs and re-storage of SscPIC2865 chromosomal integration site revealed SscPIC2865 had the ability to excise the bacterial host genome reestablishing the expected *attP* and *attB*, in the genomic island and bacterial chromosome, respectively.

Extended discussion

We determined the complete genome of a canine MDR MRSS strain from Nigeria and identified a novel staphylococcal TMP resistance gene (*dfrE*) located within a novel MDR mobilizable plasmid, which harbored additional adaptive traits. Our WGS approach allowed us to resolve its additional mobilome, unveiling high genome plasticity enriched in chromosomal and extrachromosomal novel mobile elements. Regardless the ubiquity of *S. sciuri* in different ecological niches and its proven role as reservoir for clinically relevant AMR genes, very limited WGS data are available in comparison with *S. aureus* or the CoNS *S. epidermidis*. In addition, most genome sequences are draft genomes generated by de novo assembly of short reads, which do not enable the complete determination of elements containing repeat regions, such as ISs. As observed here, ISs are highly abundant not only among AMR-carrying mobile elements, but also across the staphylococcal chromosome. Here, the combination of short-read Illumina sequencing followed by deep long-read PacBio sequencing using large DNA fragmentation allowed the determination of the complete genome and mobilome at the minimum mutation rate. Therefore, our sequencing approach evidenced the necessity for long-read sequencing to establish and complete the landscape of larger mobile elements surrounded by repeat regions. A total of 11.2% of *S. sciuri* C2865 genome consisted of the MGEs here described, which increased to over 13% when considering the length of the 78 predicted ISs (estimated IS length: 700 bp). Of note, all functionally proved AMR genes were carried by MGEs, evidencing HGT acquisition.

Phylogenomic analyses of the 30 *S. sciuri* group genomes analyzed revealed that MRSS C2865 clustered within a *S. sciuri* species sub-branch, which included three additional strains: two from human and animal infections (*S. sciuri* Z8, SNUD-18) - indicating pathogenicity potential at the genomic level - and one from food, *S. sciuri* CCUG39509. The latter was originally considered

the type strain of *S. sciuri* subsp. *carnaticus* (ATCC 700058). Nevertheless, the subspecies division into *S. sciuri* subsp. *sciuri*, *S. sciuri* subsp. *carnaticus* and *S. sciuri* subsp. *rodentium* is currently rejected (19, 20). Based on a range of phenotypic, biochemical, physiological and genetic analyses, P. Svec et al. (20) recently showed high interspecies heterogeneity with no clear differentiation into the different subspecies. While agreeing with those statements, our WG-based phylogenomic analysis revealed a clear distinction of an intraspecies sub-cluster, sharing 96% ANI with the rest of the *S. sciuri* genomes. This ANI value falls within the threshold to be considered a different species (≈95–96%) (21). Hence, we suggest that there indeed may be a subspecies discrimination among the *S. sciuri* species, but such distinction needs to be addressed by WGS comparisons and might not correspond to the outcomes retrieved by the abovementioned traditional methods. Alternatively, the available metadata of compared genomes evidence lack of clear phylogeny demarcation depending on the origin, source or host. This corroborates the low host tropism already suggested for this species (22). The pan genome analysis of all 21 *S. sciuri* genomes included revealed that MRSS C2865 significantly exhibited the highest number of both total and unique genes, and that most of them (75%) corresponded to the MGEs identified. These values highlight the particularity of MRSS C2865 as acceptor of adaptive mobile traits and the importance to search for environmental niches to understand the evolution of this exceptionally versatile bacterial species. At the species level, the open pan genome identified, with an accessory gene content of over 73%, highlights an important source of evolutionary novelty that facilitates rapid adaptation via HGT. Hence, genome-wide association studies among this species and additional commensal staphylococci are needed to identify and understand their exchange platforms and mechanisms, important to track their genome evolution.

Importantly, the novel *dfrE* alone conferred high level TMP resistance in both staphylococci and *E. coli*. A potential environmental origin is predicted, as Dhfr from soil-associated *P. anaericanus* evidenced the closest relative and DfrE was remarkably distant from the typical staphylococcal TMP resistant Dfrs. Scarce data is available on *P. anaericanus*, but bacteria belonging to this genus are ubiquitous in nature, and closest species have been reported in different environmental sources, such a soils and rhizosphere from different crops (23-25). We identified the *dfrE* gene in a limited number of staphylococcal genomes from *S. sciuri*, *S. aureus* and *S. arlettae* of human and animal origin (including clinical samples) as well as in animal-associated *M. caseolyticus* strain JCSC5402 (26) and *Exiguobacterium* sp. strain S3-2 (27). However, only in the later strain the *dfrE* gene, which authors denominated *dfr_like*, was denoted as AMR gene and proved to confer TMP resistance in *E. coli*. *Exiguobacterium* spp. are extremophiles adapted to a wide range of habitats, including cold environments. They are also recovered from closer human-associated niches, such as aquaculture, landfills, plant-derived foods, food-processing plants and pharmaceutical wastewaters (28, 29), including AMR strains (27, 30, 31). These data evidence the transferability of clinically relevant AMR genes across diverse bacteria from different taxonomic families (order Bacillales) from diverse environments. All *dfrE*-carrying genomes harbored it in MDR plasmids or plasmid-associated elements, hosting additional antibiotic and/or metal resistance genes. This co-localization is of concern, because it enables the transfer of diverse adaptive traits via a single HGT event. Importantly, in *Exiguobacterium* sp. strain S3-2, the *dfrE* gene, enclosed within plasmid pMC2, was enclosed within an integral Tn*3*-like transposon (27). This element was flanked by two typical 38-bp IRs involved in excision and integration, common in this transposon family members. pUR2865-34 harbored a truncated version of this element, as only the IS*1216E-res/rec2-res/rec3-dfrE-thy* region (4,345 bp), including the 3’-end 38-bp IR, was

conserved. Indeed, this region was highly preserved in all additional *dfrE*-carrying strains but *S. sciuri* GN5-1 pSS-04. Here, the *dfrE*-carrying region seems to have evolved afterwards by losing the *res/rec2* plus two immediate downstream genes from the Tn*3*-like remnant. This indicates that *Exiguobacterium* sp. harboured an ancestral *dfrE* (*dfr_like*) transposable element that has recombined and jumped to different staphylococcal species and to macrococci from a single ancestor. The *ica*-locus variant detected within pUR2865-34 did not prompt remarkable biofilm formation when tested in the CV assay, as formerly observed for its closest relative in pAFS11 in MRSA Rd11 (8). However, *ica*-locus variant carrying strains tested positive to the colorimetric test. Its evolutionary history and *in-vivo* activity will be the object of further characterization.

The backbone of mosaic pUR2865-34 denoted a theta replication mechanism, characteristic of larger staphylococcal plasmids. The N- and C-terminal domains of the encoded RepA were homologous to different RepA_N proteins, reflecting the ability of these proteins to undergo domain replacements, which are presumably driven by selection pressures and host/plasmid incompatibility (32). The putative Type Ib partitioning system detected in pUR2865-34 is well studied in Gram-negative bacteria, but little to nothing in Gram-positive cocci. Nevertheless, these systems contribute to the prevalence and spread of these plasmids, ensuring stable inheritance and effectively maintain resistance in the absence of selection. Although this plasmid was not conjugative, it contained a mimic of an origin of conjugative transfer, very similar to those recently identified by M. Bukowski et al. (9). Hence, pUR2865-34 could only move by conjugation via this *oriT* mimic sequence in the presence of a pWBG749-like plasmid, or via the integrated *mob* gene and associated *oriT* of pUR2865-int in the presence of a conjugative plasmid (1). However, MRSS C2865 did not harbor any member of the three known staphylococcal conjugative plasmid families (pSK41, pWBG4 and pWBG749) nor any recognizable transfer gene cluster that could act *in-*

trans. Indeed, conjugative plasmids are rare in staphylococci, representing only ca. 5% of plasmids in *S. aureus* (33).

The novel MDR SCC*mec*$_{C2865}$ enclosed additional AMR genes, remarking an IS*1216E*-flanked region carrying the tetracycline resistance gene *tet*(S), which IS copies may have facilitated its capture. This gene has been found among *Firmicutes* and Gammaproteobacteria from diverse ecological sources since 1950s (34). However, the *tet*(S) has only been detected twice before in staphylococci: (i) among MRSA isolates from animal carcasses (35) and (ii) within the *S. sciuri* SCC*mec*$_{C2865}$-related SCC*mec*TG24 cassette, from ready-to-eat meat (13). The additional closest cassette corresponded to *mecA-mecC* hybrid SCC*mec-mecC* in *S. sciuri* GVGS2 from a bovine infection (3). These strain sources highlight that *S. sciuri* from animals behave as reservoirs for mosaic MGEs carrying AMR genes from different species. CcrA/B recombinases mediate SCC*mec* excision, circularization, site-specific chromosomal integration and recombination reactions between SCC*mec att*-flanked regions. Unusually, SCC*mec*$_{C2865}$ lacked any recognizable *ccr*-encoded recombinase. The ancestral Ccrs might have been removed by IS flanking deletions rendering this cassette not mobile; however, no deleterious regions flanking the cassette were observed. Alternatively, all SCC*mec* Ccr enclose a conserved motif that was only detected in the LSR of two novel prophages C2865-pp1 and C2865-pp2, and shared closest phylogenetic distance to CcrC allotypes. Hence, even though neither circular intermediates nor an intact *rlmH* gene could be detected using the primer sets designed, the possibility that these prophage-located LSR participate in any of the abovementioned functions is tempting. Of note, virtually all staphylococci contain prophages and the transduction of small SCC*mec* types and SCC*mec* internal modules between compatible *S. aureus* strains has been demonstrated (36-38).

We detected for the first time in *S. sciuri* the integration of transposon ψTn*554* at the chromosomal *radC* gene. Further insights revealed that several *S. sciuri* genomes exhibited this *radC* gene disrupted, always by the presence of a transposon-like element - initially detected among enterococci, which harbors an AMR gene cluster carrying the emerging oxazolidinone and phenicol resistance gene *optrA* and the phenicol resistance gene *fexA* (39). This observation signals the *radC* gene of *S. sciuri* as hotspot for integration and recombination of mobile adaptive elements coming from different bacterial backgrounds.

A novel chromosome excisable PICI, named SscPIC2865, was identified. PICIs are characterized by a specific set of phage-related functions that enable them to hijack the phage lytic reproduction cycle of helper phages for their own high-efficient transduction. These elements normally carry critical staphylococcal virulence genes, such as Panton-Valentine Leukocidin, toxic-shock syndrome, enterotoxins and other superantigens (18). However, some PICIs do not carry any noticeable pathogenicity-related accessory genes. Unlike other members of the SaPI4 family, SscPIC2865 harbored a putative *mazF* toxin gene of a toxin-antitoxin system type II (*mazEF* locus) in its accessory region. MazF is a sequence-specific RNase that cleaves several transcripts, including those encoding pathogenicity factors. In addition, several staphylococcal strains from WGS projects harbored related elements at the same integration site. Particularly, *S. lentus* AE2, *S. fleurettii* MBTS-1 and *S. schleiferi* NCTC12218, either lacked an identifiable TerS or harbored a highly diverse TerS. In addition, *S. sciuri* strain BMSA-1 harboured a 3-'end mosaic structure carrying a 23S rRNA methyltransferase Erm gene sharing 78.5% nucleotide identity with the recently described MLS_B resistance gene *erm*(44)v (40). These observations evidence that SscPIC2865-related elements are common among *S. sciuri* and have counterparts or remnants in different staphylococcal species, contributing to genome plasticity and potential toxigenicity.

Hence, the ribosomal protein S18 (*rpsR* gene) appears as a hub for integration of mobile islands and should be consider when addressing staphylococcal chromosomal-integrated elements.

MRSS C2865 resulted to be a polylysogen, with three novel unrelated prophages, which accounted for 7.2% of the bacterial genome length. Despite the ecological importance of *S. sciuri* within the staphylococcal genus, only two former reports have identified *S. sciuri* phages: (i) three myoviruses from urban sewage in Poland (41), and (ii) two temperate siphophages from UV-induced *S. sciuri* strains (42), which were highly different from our novel phages. C2865-pp2 shared homology with two putative *S. sciuri* prophages; however, only C2865-pp2 enclosed a predicted adaptive gene at its accessory region: a bacterial abortive infection protein (AbiF). These systems are considered as "altruistic" cell death mechanisms that are activated by phage infection and limit viral replication, thereby providing protection to the bacterial population (43). Hence, resultant AbiF might be involved in superinfection immunity, to prevent the infection of similar phages at the bacterial population level. As recently observed by H. Oliveira et al. (14), a low rate of staphylococcal phages may exhibit a variety and uncommon number of site-specific recombinases, as it was the case for phage C2865-pp3, which enclosed 3 different integrases. Phage integrases are required for the establishment of the lysogeny, but its maintenance is also dependent on the regulatory proteins next to it (14). At least two of these three integrases were surrounded by transcriptional regulators and/or antirepresor proteins. This may enable the integration at additional chromosomal sites depending on the integrase used. In fact, the closest putative prophage enclosed within animal *S. lentus* HT5, also carrying three different integrases, was integrated at a different location. According to a recent staphylococcal phage re-classification scheme (14), C2865-pp3 falls within the *S. epidermidis* SPbeta-like singleton, which also encloses three integrases but was reported to lack genes associated with stable lysogeny maintenance. Here,

we show that a highly related phage can indeed stably reside as a prophage. Importantly, C2865-pp3 enclosed several adaptive genes, highlighting the presence of a putative type II toxin-antitoxin system of the HicA family (HicA/HicB) and an antirestriction protein (ArdA family). These elements could promote maintenance of the phage and/or avoid cell damage as well as to evade restriction in the recipient bacterium by the host restriction enzyme systems, respectively. Finally, the role of the tRNA unique to C2865-pp3, which shared homology to that present in *Listeria* spp., remains elusive, as its presence was not justified by an increased in the specific codon or amino acid usage (44). Importantly, SPbeta-like phage harbored an AMR gene cluster enclosed within a composite transposon-like element, harboring resistance determinants for aminoglycosides and TMP. This is a central feature demonstrating that C2865-pp3-related functional phages can indeed behave as reservoirs for AMR genes, which can be further spread into different bacterial hosts. Whether the different lysogenic modules of these phages are functional and widen their infectivity range and site-integration versatility still needs to be clarified, but the fact that closest relatives were retrieved from different bacterial species (*S. epidermidis*, *S. lentus*) strongly points towards this possibility. In addition, the enclosed adaptive features detected reflect the impact of staphylococcal prophages in genome evolvability.

The conservation of the integrase-generated DRs flanking the three prophage genomes, together with their ability to excise the bacterial chromosomal DNA and circularize, as well as the high genome synteny observed among unrelated (pro)phages strongly suggest that these prophages are functional. If this is the case, along with progeny generation, also plasmids or chromosomal DNA of the bacterial host may be mistakenly encapsulated (45). Although it is generally assumed that this process is mainly performed by *pac* phages (headful packaging mechanism), the ability of some *cos* phages – which are more sequence-specific - to pack determined PICIs has been also

proved (17). Indeed, phage-mediated HGT is one of the primary driving forces of bacterial evolution. However, transduction of AMR genes is poorly understood. Hence, it is conceivable that any of these prophages may package and mobilize any of the MGEs discovered here. This hypothesis is supported by the fact that no conjugative element that could mediate mobilization of any of the three plasmids was present. These prophages could also contribute to further genome evolution by mediating the mobilization of the novel *ccr*-lacking SCC*mec* element and SscPIC2865 islands. In fact, PICIs of the SaPI4 family are only induced by endogenous prophages (18). Further studies are warranted to explore the transduction ability of these prophages to further spread the novel MGEs identified.

Detailed materials and methods

DNA extraction, whole-genome sequencing, assembly and annotation

DNA was extracted by two different methods. For Illumina sequencing, the Wizard Genomic DNA Purification Kit was used including both lysozyme and lysostaphin (10 mg/ml each) (Ref. A1120, Promega Corporation, Spain). For PacBio sequencing, DNA was isolated using a phenol-chloroform method with the following modifications for improved cell lysis. Five milliliters of overnight culture in Tryptic Soy Broth (TSB) was centrifuged for at 8,000 rpm for 15 min. Cells were resuspended in 500 µl Tris-EDTA (10 mM) buffer plus 2.5 mg/ml lysozyme and 0.25 mg/ml lysostaphin and incubated for two hours at 37°C. Cells were further lysed with 1 ml of lysis buffer (20 mM Tris-HCl, 10 mM EDTA, 1% SDS) and 1 mg/ml of proteinase K for two additional hours at 37°C. Plasmid DNA was obtained using the GenElute Plasmid Miniprep Kit (Ref. PLN350, Sigma) also including a lysis step with lysozyme (2.5 mg/ml) and lysostaphin (0.25 mg/ml) for 20 min at 37°C after the resuspension solution step. All samples were eluted in 10mM Tris-HCl (pH

8). The integrity and concentration of the extracted DNA were assessed by agarose gel electrophoresis and spectrophotometric measurement (Qubit; Invitrogen), respectively.

High-throughput WGS of *S. sciuri* C2865 DNA was performed with Illumina Miseq (2 X 300 bp), with NEBNext Ultra kit (http://www.genomicsbasel.ethz.ch). For long-read sequencing, PacBio RSII was used prior DNA fragmentation of 15 kb followed by a mild size selection. Illumina read quality was checked by Fastqc (https://www.bioinformatics.babraham.ac.uk/projects/fastqc/) and reads were trimmed using Trimmomatic v0.36 (46). Good-quality Illumina reads were de-novo assembled using SPAdes (47). PacBio RSII raw reads were assembled using Canu (48). Protein-coding genes, tRNAs and rRNA operons were predicted using Prodigal (49), tRNAscan-SE and RNAmmer on both datasets (50). Predicted protein sequences were compared against the NCBI nr database using DIAMOND (51), and against COG (52) and TIGFRAM (53) using HMMscan (54) for taxonomic and functional annotation. Genomic alignment dot-plots between Illumina and PacBio resulting contigs were generated with D-GENIES software to evaluate consistency and reliability of both sequencing and assembly approaches (55). Amino acid and codon usages were determined for C2865 chromosome and prophages using the compareM package (https://github.com/dparks1134/CompareM).

Pan genome examination, *S. sciuri* group phylogenomic analysis and comparative genomics

All genomes available belonging to the *S. sciuri* group species were downloaded from the NCBI database (accessed until April 2018). In total, thirty strains (29 from NCBI, MRSS C2865) were included, which belonged to the following species (no. of strains): *S. sciuri* (21), *S. lentus* (5), *S. vitulinus* (2), *S. fleuretti* (1) and *S. stepanovicii* (1). Strains were as follow [GenBank assembly/RefSeq accession no.]: *S. sciuri* NS36 [GCA_001477335], *S. sciuri* NS1 [GCA_001476955], *S. sciuri* NS44 [GCA_001477405], *S. sciuri* RSA37 [GCA_001476585], *S.*

sciuri NS53 [GCA_001476575], *S. sciuri* NS112 [GCA_001477395], *S. sciuri* ATCC29059 [GCA_900117375], *S. sciuri* FDAARGOS285 [GCA_002209165.], *S. sciuri* LCHXa [GCA_002091355], *S. sciuri* NCTC12103 [GCA_900474615], *S. sciuri* DSM20345 [GCA_001046995], *S. sciuri* MC10_S56 [GCA_003006205], *S. sciuri* P575 [GCA_001766775], *S. sciuri* S P879 [GCA_001766785], *S. sciuri* SAP15-1 [GCA_001684285], *S. sciuri* NS202 [GCA_001476555], *S. sciuri* i1 [GCA_002407445], *S. sciuri* SNUD18 [GCA_002072755], *S. sciuri* CCUG39509 [GCA_002902225], *S. sciuri* Z8 [GCA_000612145], *S. lentus* MF1767 [GCA_001651345], *S. lentus* 050AP [GCA_900098655], *S. lentus* MF1862 [GCA_001651255], *S. lentus* NCTC12102 [GCA_900458735], *S. lentus* F1142 [GCA_000286395], *S. vitulinus* DSM15615 [GCA_002902265], *S. vitulinus* F1028 [GCA_000286335], *S. fleuretti* MBTS1 [GCA_002018435] and *S. stepanovicii* NCTC13839 [GCA_900187075]. In addition, *S. aureus* NCTC8325 [NC_007795] and *S. epidermidis* ATCC 12228 [NC_004461] were included as reference genomes used as outgroup for the genus level.

In order to measure the probability of two genomes belonging to the same species, an ANI of the 30 *S. sciuri* group genomes was calculated using JSpecies as indicated before (21). A heat map was generated using the ANI matrix output table with R (56). A maximum likelihood tree for all the *S. sciuri* group genomes was generated using RAxML (version 7.2.6) (57) using core alignment obtain with Parsnp software within Harvest Suite package (58). Before phylogenomic analysis, we removed from the alignment all the genomic regions where recombination was detected. These regions were determined using the Gingr software, also included in the Harvest Suite package (82), which uses as input the output obtained directly from the Pasnp software. The results were visualized using iTOL v6 (https://itol.embl.de/).

BLAST Ring Image Generator (BRIG) was used to evaluate and visualize BLAST comparisons (blastn, -evalue Expectation value 10.0) of a particular percentage identity (100, 70, 50) between MRSS strain C2865 and its closest genomes (>98% ANI), using MRSS C2865 genome as reference with the *dnaA* gene set at position 1 (59). Here, sequences that are not present in the reference genome are not represented. Pan genome analysis (core plus accessory genome) for the 21 *S. sciuri* genomes was carried out using Roary with a 95% identity cutoff value (60). The parameters were defined as follows: Core genes: ≥ 99% of analyzed genomes, accessory genes: 1 - 99% (soft core 95-99%; shell 15-95%; cloud ≤ 15%).

Detection and analysis of resistome and mobilome from *S. sciuri* C2865

Antimicrobial resistance (AMR) genes. AMR genes formerly detected in MRSS C2865 were blasted against the WGS of strain C2865. Contig/s were manually checked for redundant *dhfr* genes and for additional resistance genes of interest. Specific PCRs followed by Sanger sequencing were performed for scaffolding AMR-carrying contigs of interest or to double check assembly correctness (position and orientation) (Illumina data).

Plasmids. Plasmid contig identification and plasmid reconstruction was achieved by contig coverage, sequence similarity with plasmid backbone genes (i.e. replication initiation proteins), gene composition and organization and contig circularity. For this, candidate contigs were blasted against the NCBI database and PasmidFinder 2.0 (61). In parallel, all *S. sciuri* and *S. lentus* plasmids deposited on the NCBI were downloaded and blasted against all C2865 contigs. When needed, PCR and Sanger sequencing was performed for scaffolding (Illumina data). Putative *oriT* and *oriT* mimics on the mobilizable elements were searched using the core sequence of those from conjugative plasmids (62). For RCR plasmids, *dso* and *sso* regions, involved in the initiation of replication of the leading and lagging strand, respectively, were searched using core sequences of

a RCR representative per plasmid family (63). The secondary structures of *dso, sso, oriT* and *oriT* mimic were generated using Mfold web server for single-stranded linear DNA at default parameters (64).

Prophages. Phaster website was used to identify phage containing regions and phage contigs (Illumina data) (http://phaster.ca/) (65). Manual inspection of phage-associated genes (morphology/structure, lysogeny, cell lysis, DNA metabolism) and characteristic functional modular organization was implemented for phage confirmation and integrity. Phage-related integrases belong either to the λ family of tyrosine recombinases (Y-rec), with conserved domain PF00589, or to a subgroup of the serine recombinase superfamily, the large serine recombinases (LSR), which have larger C-terminal domains compared to serine recombinases (S-rec) of the resolvases/invertases subfamily, responsible for coordination of unique LSR activities (66). For identification of LSR phage integrases, both the N-terminal catalytic domain (Pfam ID PF00239) and the DNA-binding recombinase domain (Pfam ID PF07508) had to be recognized (67). For this, motif and domain analysis of the translated candidate CDSs was performed against ScanProsite database (68), Pfam database (69), and NCBI conserved domain database (CDD) (70). Integrase-directed generation of DRs as a result of genome integration was investigated manually by sequence comparison of bacterial chromosome-prophage boundaries with corresponding regions of MRSS C2865 and a *S. sciuri* strain prototype (SNUDS-18, Genbank ac. No. CP020377) lacking those prophages. To analyze prophage chromosomal integration sites with those of similar integrase-carrying prophages, prophage integrases were blasted against the non-redundant NCBI NR database. Genomic regions harboring integrases sharing >80% ID and >70% coverage were selected and prophage boundaries were likewise analyzed. ANI (genome-to-genome) pairwise comparison between C2865-enclosed prophages and all staphylococcal phage genomes available

in the Viral RefSeq database (as of November 2018, n=187) were calculated using the JSpecies with default parameters (21). A heat map was generated using the ANI matrix output table with R (56).

Staphylococcal Chromosomal Cassette mecA (SCC*mec*). ORFs found downstream of the integration gene 23S rRNA (pseudouridine(1915)-N(3))-methyltransferase RlmH, initially known as OrfX, as well as regions containing characteristic SCC*mec* genes (*mecA, mecR, mecI, ccr*) were analyzed. To detect *ccr* gene/s, which resultant proteins belong to the LSR family, CcrA and CcrB proteins from reference methicillin-resistant *S. sciuri* TXG-24 (GenBank acc. No KX774481) (13) were blasted against MRSS C2865 translated contigs (NCBI tblastx). Translated candidate genes were scanned for the motif constituting the residues in the active site of S-rec. For this, consensus motif Y-[LIVAC]-R-[VA]-S-[ST]-x (2)-Q, derived from Prosite entry PS00397 (http://prosite.expasy.org) was used. Here, the catalytic serine residue is indicated in bold, amino acids acceptable for one given position appear between square brackets and "x" stands for any amino acid followed by the possible repetition number within the parentheses (71). Gene candidates containing this motif were scanned as above (ScanProsite, Pfam; NCBI CDD) for LSR characteristic domains (PF00239 and PF07508, and often PF13408). Additional S-rec encompassing the Y-[LIVAC]-R-[VA]-S-[ST]-x (4)-Q motif were likewise screened along the entire MRSS C2865 genome. Putative ISS for SCC*mec* or *att* core sites recognized by the typical staphylococcal Ccr were manually identified by searching for the consensus sequence 5'-GAAGC[AG]TATCA[TC]AAAT[AG]A-3' (positions with possible alternative nucleotides are indicated in square brackets).

Others. Additional recombinases associated with MGEs, i.e. Y-rec of phage or phage-derived elements, such as SaPI – or as recently denoted PICI -and Tnp, as well as S-rec of the

resolvases/invertases subfamily (which enclose an N-terminal catalytic domain and a DNA-binding recombinase domain belonging to the HTH superfamily), were investigated as above for motif and domain analysis in translated gene candidates of MGEs. SscPI chromosomal integration site was compared with similar integrase-carrying genomic islands. For this, SscPI Y-rec was blasted against the non-redundant BLASTp NCBI database. Genomic regions harboring integrases sharing >80% ID and >70% coverage were selected. Integrase-directed generation of DRs as a result of SscPI integration were investigated by sequence comparison of bacterial chromosome-SscPI boundaries with corresponding regions of *S. sciuri* strain SNUDS-18 (Genbank ac. No. CP020377), which lacks any insertion in corresponding chromosomal region. In addition, ISsaga2 web tool was used for IS identification and quantification (www-is.biotoul.fr) (72).

Excision ability of chromosomally located MGEs. Potential excision and circularization of selected chromosomally located mobile elements, in addition to detection of resultant chromosomal region after excision were tested by specific inverse and conventional PCR, respectively (see Table S1 in reference (10)). For this, PCR MasterMix (2X) (#K0172, ThermoFisher Scientific) was used according to provider indications. An annealing temperature of 56°C and an extension time of one minute per kilobase of amplified product were used. PCR products were run on 1% agarose gels for 45 min at 6 V/cm. Positive amplicons were subjected to Sanger sequencing with both primers (Microsynth, Germany) and attachment regions encompassing the recognition core sites were analyzed.

Phylogenetic analyses of proteins of interest

Phylogenetic analyses of closest Dhfrs (percentage of identity >50%) as well as former staphylococcal TMP resistance Dfrs (DfrA, DfrD, DfrG, DfrK, DfrF) were investigated by the construction of an unrooted phylogenetic network with SplitsTree v4 (73), using a neighbor-net

with default parameters. TMP resistance dihydrofolate reductases included originated from *S. sciuri* C2865 (DfrE), *S. aureus* pSK1 (DfrA) (GU565967), S. *haemolyticus* MUR313 (DfrD) (NG_047754), *S. aureus* CFSA173 (DfrF) (NZ_NDQI01000082), *S. aureus* CMS2 (DfrG) (NG_047756) and *S. aureus* 2187 (DfrK) (FM207105). In addition, Dhfrs were included from the following organisms: *Paenibacillus* UNC451MF (WP_028548169), *Paenibacillus* 32352 (WP_079908945) *P. anaericanus* (WP_127193417), *P. contaminans* (WP_113036777), *Anaeromicrobium sediminis* (WP_095136168), *Clostridium kluyveri* (WP_073540407), *C. vincentii* (WP_106061057), *C. cellulovorans* 743B (WP_010075211), *C. Maddingley* MBC34-26 (WP_008427509), *Streptococcus suis* BM407 (YP_003028702), *Coprobacillus* AF18-40 (RGT84250), *Pseudobacteroides cellulosolvens* ATCC35603 (WP_036943100), Deltaproteobacteria bacterium HGW-16 (PKN49713), Deltaproteobacterium PSCGC_5451 (WP_027985810), uncultured bacterium (AIA11975) and Deltaproteobacteria bacterium RIFOXYD12_FULL_56_24_OGQ88340. Evidence for phylogenetic heterogeneity due to recombination was conducted with SplitsTree v4 using the Phi test for recombination option.

Phylogenetic analysis of all *S. sciuri* group Dhfrs was in parallel generated. The alignment was performed with (i) the entire dihydrofolate reductase (Dhfr) protein(s) present in all *S. sciuri* species group genomes deposited in the NCBI database (accessed until April 2018), (ii) both Dhfrs present in *S. sciuri* strain C2865, and (iii) the amino acid sequence of all trimethoprim resistance dihydrofolate reductases (Dfr) described so far in staphylococci (DfrA, DfrD, DfrG, DfrK, DfrF), as well as (iv) the Dhfr of trimethoprim susceptible *S. epidermidis* ATCC 12228 (NC_004461) and *S. aureus* ATCC 25923 (Z16422) as reference. Of note, when more than one dfr gene was present per genome, both resultant proteins were included and labeled “Dhfr1” and “Dhfr2,” based on the genome position as deposited in NCBI, respectively. Amino acid sequence alignment was

performed using MUSCLE, with UPGMB as clustering method. The tree was built by the neighbor-joining method using MEGA 7.0.21 program (bootstrap of 1,000 replications (substitution model: poison model; rates among sites: gamma distribution) (74).

One representative staphylococcal and macrococcal Ccr per type (originating from SCC*mec* I to the recently described SCC*mec* XIII) (75, 76), in addition to all MRSS C2865 LSR harboring the Ccr consensus motif Y-[LIVAC]-R-[VA]-**S**-[ST]-x (2)-Q or Y-[LIVAC]-R-[VA]-**S**-[ST]-x (4)-Q were equally analyzed. Represented Ccrs originate from SCC*mec* I of *S. aureus* strain NCTC10442 (Genbank acc. No. AB033763) (CcrA1 and CcrB1); SCC*mec* II of *S. aureus* strain N315 (BA000018) (CcrA2 and CcrB2); SCC*mec* III of *S. aureus* strain 85/2082 (AB037671) (CcrA3 and CcrB3); SCC*mec* VI of *S. aureus* strain HDE288 (AF411935) (CcrA4 and CcrB4); SCC*mec* VII of *S. aureus* strain JCSC6082 (AB373032) (CcrC1); SCC*mec* X of *S. aureus* strain JCSC6945 (AB505630) (CcrB6); SCC*mec* XII of *S. aureus* strain BA01611 (KR187111) (CcrC2); SCC*mec* VII-241 of *S. pseudintermedius* strain KM241 (AM904731*)* (CcrA5 and CcrB5); and SCC*mec*KM45013 of *Macrococcus canis* KM45013 (HG970732) (CcrAm2 and CcrBm2).

In order to disclose phage integrase phylogenetic relationship of prophage integrases, a maximum likelihood phylogenetic tree with amino acid sequences of (i) MRSS C2865 prophage integrases, (ii) all integrases sharing >80% amino acid identity and >70% coverage, and (iii) Enterobacteria lambda phage integrase (RefSeq acc. No. NC_001416), used as outgroup, were included. Amino acid sequence alignment was performed using MUSCLE and the tree was built by the Neighbour-joining method with a bootstrap of 500 replications (protein substitution model: WAG) (CLC Genomics Workbench 11). To estimate the packaging mechanism of identified prophages, a circular phylogenetic tree of the terminase large subunit (TerL) from MRSS C2865 prophages and those identified in the 187 staphylococcal phages deposited in the Viral RefSeq database (accessed

until November 2018) was likewise created upon amino acid sequence alignment using CLUSTALW (CLC Genomics Workbench 11). TerL of Enterobacteria phage lambda (RefSeq, accession no. NC_001416) was used as outlier. TerL of staphylococcal phages with known packaging mechanism was indicated (16).

Phylogenetic analyses of all site-specific tyrosine recombinases and terminase small subunits (TerS) in the NCBI NR database sharing >80% amino acid identity to those of SscPI in C2865 were performed by the construction of a Maximum likelihood tree. For this, Amino acid sequence alignment of respective proteins from NCBI NR (NCBI non-redundant database) was generated using MUSCLE, with UPGMB as clustering method. A phylogenetic tree was built by the Maximum-likelihood method using the MEGA 7.0.21 program (bootstrap of 500 replications, substitution model: poison model; rates among sites: Gamm Distribution) (74). The evolutionary analyses of the Integrases and terminase small subunits (TerS) of the staphylococcal pathogenicity island SscPIC2865 were conducted in the MEGA 7.0.21 program as indicated above (bootstrap of 500 replications, substitution model: poison model; rates among sites: Gamm Distribution) (74). All trees were drawn to scale, with branch lengths measured in the number of substitutions per site. The percentage of trees in which the associated taxa clustered together is shown next to the branches.

Construction and selection of recombinant plasmids carrying the *dfrE* TMP resistance gene

To address functionality of the candidate TMP resistance gene, as well as for potential synergistic activity by its immediate 3'-end thymidylate synthase gene (*thy*), three different *dfrE* containing regions were amplified: (i) *dfrE* gene alone, (ii) *dfrE* gene plus flanking regions, and (iii) *dfrE* gene, *thy* gene and flanking regions (Table S2). *Staphylococcus aureus* - *Escherichia coli* shuttle vectors pBUS-HC and pBUS-Pcap-HC were used for cloning (77). The 495-bp PCR-amplified

dfrE gene was cloned into the multiple cloning site (MCS) of pBUS-Pcap-HC, which contains a constitutive promoter, leaving *dfrE* expression under its control. The other amplified fragments were cloned into the MCS of pBUS-HC vector, allowing the expression of *dfrE* under the control of its native promoter. Designed primers for inserts and vectors amplification are listed in S1 Table at [https://doi.org/10.1101/2020.09.30.320143].

All purified plasmid DNA fragments were amplified by PCR using Phusion DNA polymerase (Thermo Scientific) and purified on silica columns (Wizard SV Gel and PCR Clean-Up System, Promega). After removal of plasmid template by *DpnI* digestion (20 Units *DpnI*, 30 min at 37°C), recombinant plasmids were assembled with the Gibson method (NEBuilder HiFi DNA Assembly Cloning Kit, New England Biolabs) for 1 h at 50 °C. For every assembly reaction, 0.1 pmol of purified vector DNA and 0.3 pmol of insert DNA (1:3 ratio) was used per sample in a 20-µL reaction. Two microliters of chilled assembly product were used as input DNA for transformation experiments on chemically competent *E. coli* DH5α, following provider recommendations. Transformants were selected on TSB agar supplemented with 15 µg/ml of tetracycline (Sigma-Aldrich), and clones were confirmed to contain inserted fragments by PCR and sequencing.

Transfer experiments and analysis of plasmid integrity

Electrocompetent cells of restriction-deficient *S. aureus* RN4220 were used for electroporation of 1 µg (6 µl maximum volume) purified plasmid DNA from C2865, the 3 recombinant plasmids, and both empty vectors (pBUS-HC, pBUS-Pcap-HC). Electroporation pulse was set at 1.8 kV, 1’000 Ω and 30 µF, aiming a time constant of 2.7. Transformants were selected on TSB agar supplemented with 16 µg/ml of TMP (Sigma-Aldrich) or 15 µg/ml of tetracycline (for pBUS1 transformants), and clones were confirmed to contain plasmid of interest by plasmid DNA extraction, PCR and sequencing, and susceptibility testing.

Purified DNA of two independent transformants containing the original *dfrE* gene (RN4220/pUR2865-34) were sent for Illumina WGS (HiSeq, 10 Mill reads, 2 x 150 bp) for confirmation of integrity of transformed plasmid. In parallel, one microgram of *dfrE*-containing plasmid DNA from selected transformants (RN4220/pUR2865-34) and from original strains (C2865, C2853, C2854, C2855) were digested with 1 Unit of S1 enzyme (1 min at 37°C) (Thermo Fisher Scientific) for plasmid linearization, and gel electrophoresis was run for 18 h at 3V/cm for analysis of plasmid length and integrity.

Antimicrobial susceptibility testing

MIC of TMP was determined by the agar dilution method on Mueller-Hinton plates (MH, Becton Dickinson) (78). Assays were performed in duplicate in three independent experiments. For this, the original strains C2865, C2853, C2854, C2855, the recipient strains *S. aureus* RN4220 and *E. coli* DH5α, and all *S. aureus* RN4220 and *E. coli* DH5α transformants were tested. In addition, *S. aureus* DSM 2569 was used as reference for all assays. TMP concentration ranges were 0.5 to 4'096 µg/ml. Plates were incubated for 20 h at 37°C under aerobic conditions and analysed according to Clinical and Laboratory Standards Institute (CLSI) guidelines. The agar disk-diffusion method was also used to test for AMR profile of transformants (78). Antimicrobials tested were as follows (µg/disk): erythromycin (15), clindamycin (2), gentamicin (10), kanamycin (30), streptomycin (10 U), tobramycin (10), tetracycline (30), thimethoprim (5), sulfonamide (300) and TMP–sulfamethoxazole (1.25 + 23.75).

Phenotypic characterization of biofilm formation

Biofilm formation ability of the four *S. sciuri* strains as well as *S. aureus* RN4220/pUR2865-34 transformants was tested by Congo Red Agar (CRA) assay and by microtitre plate assay (static

biofilm assay with crystal violet). *S. aureus* SA113 (DSM 4910), an *S. aureus* strain derived from the laboratory strain NCTC 8325, and *S. aureus* ATCC 25923 (DSM 1104) were used as positive controls for their strong biofilm-forming potential, while *S. aureus* RN4220 (DSM 26309) was selected as negative control for biofilm production. Colony morphology was determined using a modified CRA (CRAmod) that consists of Brain Hearth Infusion (BHI) agar with sucrose (5%), Congo red (0.08%), NaCl (1.5%) and glucose (2%). A 4-µL aliquot of a bacterial suspension with 10^8 CFU/mL (OD_{600}) was inoculated in a spot and incubated at 37 °C under aerobic conditions for 24h (79). Each strain was tested in duplicates and the assay was repeated twice.

For the microtitre plate test, an *ica*-negative non-biofilm producing methicillin-resistant *Staphylococcus lentus* strain (C3030) (80) was additionally included. Staphylococcal strains were grown overnight in TSB at 37°C. Fresh overnight cultures were diluted 1:200 with TSB medium containing 0.25% glucose, and 200 µL per well were seeded in sterile 96-well polystyrene culture plates and further incubated for 24 h at 30 °C. Cultures were carefully removed and the attached bacteria were fixed with 200 µl of 99% methanol for 15 min at room temperature (RT). Wells containing the attached biofilms were dried for 10 min at RT. Samples were washed once in phosphate buffered saline (PBS) solution (pH 7.2), the adherent biofilms were stained with 200 µl of 0.4% crystal violet for 20 min at RT. Samples were then washed three times with PBS and dried at RT. The stain was dissolved in 33% acetic acid (1 hour at RT) and diluted 1/10 in water when needed. The absorbance of the biofilm was then measured at 590 nm (OD_{590}) in a microplate reader (FLUOstar Omega – BMG Labtech). Uninoculated wells containing TSB with glucose served as blanks. The blank corrected absorbance values were used for reporting biofilm production. Strains producing a mean absorbance value of > 0.3 were considered as weak biofilm producers. They

were considered higher biofilm producer if the value was higher than 1.0. Each strain was tested for biofilm production in duplicates and the assay was repeated thrice (81).

PCR detection of the TMP resistance gene *dfrE* and the *ica* locus variant

Primers were designed for the detection of the TMP resistance *dfrE* gene, the biofilm formation *ica* locus (*ica*ADBC) variant genes and the *ica* locus repressor (*icaR*) (see S1 Table at [https://doi.org/10.1101/2020.09.30.320143]). Primers for the *dfrE* gene were designed within the Dhfr superfamily domain (PF00186) (CD-Search), and all *dhfr* genes with ≥ 45% nucleotide similarity were included to search for specificity of *dfrE* gene primer set. Originaol strains (C2853, C2854, C2855) and RN4220/pUR2865-34 transformants were tested. DNA from C2865 and RN4220 were used as positive and negative controls, respectively. In addition, *Staphylococcus pseudintermedius* strain C2381, carrying the *dfrG* gene, and *S. pseudintermedius* strain C4670, carrying the *dfrK* gene, were included to test *dfrE* primer set specificity and sensitivity (82, 83). PCR amplification was performed using proof-reading Phusion DNA polymerase according to provider indications (#F530S, ThermoFisher Scientific). For the *dfrE* gene, an annealing temperature of 47°C and an extension time of 45 sec was used. For genes belonging to the i*ca*ADBC and for *icaR* an annealing temperature of 55°C and an extension time of 1 min was used. PCR products were run on 1% agarose gels for 45 min at 6 V/cm and Sanger sequenced.

References

1. Firth N, Jensen SO, Kwong SM, Skurray RA, Ramsay JP. 2018. Staphylococcal Plasmids, Transposable and Integrative Elements. Microbiol Spectr 6.
2. Fraunholz M, Bernhardt J, Schuldes J, Daniel R, Hecker M, Sinha B. 2013. Complete Genome Sequence of Staphylococcus aureus 6850, a Highly Cytotoxic and Clinically Virulent Methicillin-Sensitive Strain with Distant Relatedness to Prototype Strains. Genome Announc 1.

3. Harrison EM, Paterson GK, Holden MT, Ba X, Rolo J, Morgan FJ, Pichon B, Kearns A, Zadoks RN, Peacock SJ, Parkhill J, Holmes MA. 2014. A novel hybrid SCCmec-mecC region in Staphylococcus sciuri. J Antimicrob Chemother 69:911-8.
4. Koonin EV, Ilyina TV. 1993. Computer-assisted dissection of rolling circle DNA replication. Biosystems 30:241-68.
5. del Solar G, Giraldo R, Ruiz-Echevarria MJ, Espinosa M, Diaz-Orejas R. 1998. Replication and control of circular bacterial plasmids. Microbiol Mol Biol Rev 62:434-64.
6. Ruiz-Maso JA, Macho NC, Bordanaba-Ruiseco L, Espinosa M, Coll M, Del Solar G. 2015. Plasmid Rolling-Circle Replication. Microbiol Spectr 3:PLAS-0035-2014.
7. Kuroda M, Ohta T, Uchiyama I, Baba T, Yuzawa H, Kobayashi I, Cui L, Oguchi A, Aoki K, Nagai Y, Lian J, Ito T, Kanamori M, Matsumaru H, Maruyama A, Murakami H, Hosoyama A, Mizutani-Ui Y, Takahashi NK, Sawano T, Inoue R, Kaito C, Sekimizu K, Hirakawa H, Kuhara S, Goto S, Yabuzaki J, Kanehisa M, Yamashita A, Oshima K, Furuya K, Yoshino C, Shiba T, Hattori M, Ogasawara N, Hayashi H, Hiramatsu K. 2001. Whole genome sequencing of meticillin-resistant Staphylococcus aureus. Lancet 357:1225-40.
8. Fessler AT, Zhao Q, Schoenfelder S, Kadlec K, Brenner Michael G, Wang Y, Ziebuhr W, Shen J, Schwarz S. 2017. Complete sequence of a plasmid from a bovine methicillin-resistant Staphylococcus aureus harbouring a novel ica-like gene cluster in addition to antimicrobial and heavy metal resistance genes. Vet Microbiol 200:95-100.
9. Bukowski M, Piwowarczyk R, Madry A, Zagorski-Przybylo R, Hydzik M, Wladyka B. 2019. Prevalence of Antibiotic and Heavy Metal Resistance Determinants and Virulence-Related Genetic Elements in Plasmids of Staphylococcus aureus. Front Microbiol 10:805.
10. Gómez-Sanz E, Haro-Moreno JM, Jensen SO, Roda-Garcia JJ, Lopez-Perez M. 2020. Staphylococcus sciuri C2865 from a distinct subspecies cluster as reservoir of the novel transferable trimethoprim resistance gene, dfrE, and adaptation driving mobile elements. bioRxiv DOI: 101101/20200930320143 doi:10.1101/2020.09.30.320143.
11. Jansen WT, Beitsma MM, Koeman CJ, van Wamel WJ, Verhoef J, Fluit AC. 2006. Novel mobile variants of staphylococcal cassette chromosome mec in Staphylococcus aureus. Antimicrob Agents Chemother 50:2072-8.
12. Harrison EM, Paterson GK, Holden MT, Morgan FJ, Larsen AR, Petersen A, Leroy S, De Vliegher S, Perreten V, Fox LK, Lam TJ, Sampimon OC, Zadoks RN, Peacock SJ, Parkhill J, Holmes MA. 2013. A Staphylococcus xylosus isolate with a new mecC allotype. Antimicrob Agents Chemother 57:1524-8.
13. Yang TY, Hung WW, Lin L, Hung WC, Tseng SP. 2017. mecA-related structure in methicillin-resistant coagulase-negative staphylococci from street food in Taiwan. Sci Rep 7:42205.
14. Oliveira H, Sampaio M, Melo LDR, Dias O, Pope WH, Hatfull GF, Azeredo J. 2019. Staphylococci phages display vast genomic diversity and evolutionary relationships. BMC Genomics 20:357.
15. Mahony J, Alqarni M, Stockdale S, Spinelli S, Feyereisen M, Cambillau C, Sinderen DV. 2016. Functional and structural dissection of the tape measure protein of lactococcal phage TP901-1. Sci Rep 6:36667.
16. GutiÃ©rrez D, Adriaenssens EM, MartÃnez B, RodrÃguez A, Lavigne R, Kropinski AM, GarcÃa P. 2014. Three proposed new bacteriophage genera of staphylococcal phages: "3alikevirus", "77likevirus" and "Phietalikevirus". Arch Virol 159:389-98.
17. Quiles-Puchalt N, Carpena N, Alonso JC, Novick RP, Marina A, Penades JR. 2014. Staphylococcal pathogenicity island DNA packaging system involving cos-site packaging and phage-encoded HNH endonucleases. Proc Natl Acad Sci U S A 111:6016-21.

18. Novick RP, Christie GE, Penades JR. 2010. The phage-related chromosomal islands of Gram-positive bacteria. Nat Rev Microbiol 8:541-51.
19. Kloos WE, Ballard DN, Webster JA, Hubner RJ, Tomasz A, Couto I, Sloan GL, Dehart HP, Fiedler F, Schubert K, de Lencastre H, Sanches IS, Heath HE, Leblanc PA, Ljungh A. 1997. Ribotype delineation and description of Staphylococcus sciuri subspecies and their potential as reservoirs of methicillin resistance and staphylolytic enzyme genes. Int J Syst Bacteriol 47:313-23.
20. Svec P, Petras P, Pantucek R, Doskar J, Sedlacek I. 2016. High intraspecies heterogeneity within Staphylococcus sciuri and rejection of its classification into S. sciuri subsp. sciuri, S. sciuri subsp. carnaticus and S. sciuri subsp. rodentium. Int J Syst Evol Microbiol 66:5181-5186.
21. Richter M, Rossello-Mora R. 2009. Shifting the genomic gold standard for the prokaryotic species definition. Proc Natl Acad Sci U S A 106:19126-31.
22. Nemeghaire S, Argudin MA, Fessler AT, Hauschild T, Schwarz S, Butaye P. 2014. The ecological importance of the Staphylococcus sciuri species group as a reservoir for resistance and virulence genes. Vet Microbiol 171:342-56.
23. Xiang W, Wang G, Wang Y, Yao R, Zhang F, Wang R, Wang D, Zheng S. 2014. Paenibacillus selenii sp. nov., isolated from selenium mineral soil. Int J Syst Evol Microbiol 64:2662-2667.
24. Huang H, Feng F, Liu M, Zhang F, Sun Q, Qin S, Bao S. 2016. Paenibacillus segetis sp. nov., isolated from soil of a tropical rainforest. Int J Syst Evol Microbiol 66:3703-3707.
25. Chen Z, Ouyang W, Chen Y, Tian W, Sun L. 2019. Paenibacillus zeisoli sp. nov., isolated from maize-cultivated soil artificially contaminated with cadmium. Int J Syst Evol Microbiol 69:1149-1154.
26. Baba T, Kuwahara-Arai K, Uchiyama I, Takeuchi F, Ito T, Hiramatsu K. 2009. Complete genome sequence of Macrococcus caseolyticus strain JCSCS5402, [corrected] reflecting the ancestral genome of the human-pathogenic staphylococci. J Bacteriol 191:1180-90.
27. Yang J, Wang C, Wu J, Liu L, Zhang G, Feng J. 2014. Characterization of a multiresistant mosaic plasmid from a fish farm Sediment Exiguobacterium sp. isolate reveals aggregation of functional clinic-associated antibiotic resistance genes. Appl Environ Microbiol 80:1482-8.
28. Castro-Severyn J, Remonsellez F, Valenzuela SL, Salinas C, Fortt J, Aguilar P, Pardo-Este C, Dorador C, Quatrini R, Molina F, Aguayo D, Castro-Nallar E, Saavedra CP. 2017. Comparative Genomics Analysis of a New Exiguobacterium Strain from Salar de Huasco Reveals a Repertoire of Stress-Related Genes and Arsenic Resistance. Front Microbiol 8:456.
29. Lei J, Zheng M, Wang L, Yin G, Lou Y, Shi L. 2020. Complete genome sequence of Exiguobacterium mexicanum A-EM, isolated from seafloor hydrothermal vents in Atlantic Ocean. Mar Genomics doi:10.1016/j.margen.2020.100801:100801.
30. Noor Uddin GM, Larsen MH, Guardabassi L, Dalsgaard A. 2013. Bacterial flora and antimicrobial resistance in raw frozen cultured seafood imported to Denmark. J Food Prot 76:490-9.
31. Tahrani L, Soufi L, Mehri I, Najjari A, Hassan A, Van Loco J, Reyns T, Cherif A, Ben Mansour H. 2015. Isolation and characterization of antibiotic-resistant bacteria from pharmaceutical industrial wastewaters. Microb Pathog 89:54-61.
32. Perez-Roth E, Kwong SM, Alcoba-Florez J, Firth N, Mendez-Alvarez S. 2010. Complete nucleotide sequence and comparative analysis of pPR9, a 41.7-kilobase conjugative staphylococcal multiresistance plasmid conferring high-level mupirocin resistance. Antimicrob Agents Chemother 54:2252-7.
33. Ramsay JP, Firth N. 2017. Diverse mobilization strategies facilitate transfer of non-conjugative mobile genetic elements. Curr Opin Microbiol 38:1-9.
34. Roberts MC. 2005. Update on acquired tetracycline resistance genes. FEMS Microbiol Lett 245:195-203.

35. Moon DC, Tamang MD, Nam HM, Jeong JH, Jang GC, Jung SC, Park YH, Lim SK. 2015. Identification of livestock-associated methicillin-resistant Staphylococcus aureus isolates in Korea and molecular comparison between isolates from animal carcasses and slaughterhouse workers. Foodborne Pathog Dis 12:327-34.
36. Scharn CR, Tenover FC, Goering RV. 2013. Transduction of staphylococcal cassette chromosome mec elements between strains of Staphylococcus aureus. Antimicrob Agents Chemother 57:5233-8.
37. Maslanova I, Doskar J, Varga M, Kuntova L, Muzik J, Maluskova D, Ruzickova V, Pantucek R. 2013. Bacteriophages of Staphylococcus aureus efficiently package various bacterial genes and mobile genetic elements including SCCmec with different frequencies. Environ Microbiol Rep 5:66-73.
38. Chlebowicz MA, Maslanova I, Kuntova L, Grundmann H, Pantucek R, Doskar J, van Dijl JM, Buist G. 2014. The Staphylococcal Cassette Chromosome mec type V from Staphylococcus aureus ST398 is packaged into bacteriophage capsids. Int J Med Microbiol 304:764-74.
39. Fan R, Li D, Fessler AT, Wu C, Schwarz S, Wang Y. 2017. Distribution of optrA and cfr in florfenicol-resistant Staphylococcus sciuri of pig origin. Vet Microbiol 210:43-48.
40. Strauss C, Hu Y, Coates A, Perreten V. 2017. A Novel erm(44) Gene Variant from a Human Staphylococcus saprophyticus Isolate Confers Resistance to Macrolides and Lincosamides but Not Streptogramins. Antimicrob Agents Chemother 61.
41. Jurczak-Kurek A, Gasior T, Nejman-Falenczyk B, Bloch S, Dydecka A, Topka G, Necel A, Jakubowska-Deredas M, Narajczyk M, Richert M, Mieszkowska A, Wrobel B, Wegrzyn G, Wegrzyn A. 2016. Biodiversity of bacteriophages: morphological and biological properties of a large group of phages isolated from urban sewage. Sci Rep 6:34338.
42. Zeman M, Maslanova I, Indrakova A, Siborova M, Mikulasek K, Bendickova K, Plevka P, Vrbovska V, Zdrahal Z, Doskar J, Pantucek R. 2017. Staphylococcus sciuri bacteriophages double-convert for staphylokinase and phospholipase, mediate interspecies plasmid transduction, and package mecA gene. Sci Rep 7:46319.
43. Dy RL, Przybilski R, Semeijn K, Salmond GP, Fineran PC. 2014. A widespread bacteriophage abortive infection system functions through a Type IV toxin-antitoxin mechanism. Nucleic Acids Res 42:4590-605.
44. Bailly-Bechet M, Vergassola M, Rocha E. 2007. Causes for the intriguing presence of tRNAs in phages. Genome Res 17:1486-95.
45. Haaber J, Leisner JJ, Cohn MT, Catalan-Moreno A, Nielsen JB, Westh H, Penades JR, Ingmer H. 2016. Bacterial viruses enable their host to acquire antibiotic resistance genes from neighbouring cells. Nat Commun 7:13333.
46. Bolger AM, Lohse M, Usadel B. 2014. Trimmomatic: a flexible trimmer for Illumina sequence data. Bioinformatics 30:2114-20.
47. Bankevich A, Nurk S, Antipov D, Gurevich AA, Dvorkin M, Kulikov AS, Lesin VM, Nikolenko SI, Pham S, Prjibelski AD, Pyshkin AV, Sirotkin AV, Vyahhi N, Tesler G, Alekseyev MA, Pevzner PA. 2012. SPAdes: a new genome assembly algorithm and its applications to single-cell sequencing. J Comput Biol 19:455-77.
48. Koren S, Walenz BP, Berlin K, Miller JR, Bergman NH, Phillippy AM. 2017. Canu: scalable and accurate long-read assembly via adaptive k-mer weighting and repeat separation. Genome Res 27:722-736.
49. Hyatt D, Chen GL, Locascio PF, Land ML, Larimer FW, Hauser LJ. 2010. Prodigal: prokaryotic gene recognition and translation initiation site identification. BMC Bioinformatics 11:119.
50. Lowe TM, Eddy SR. 1997. tRNAscan-SE: a program for improved detection of transfer RNA genes in genomic sequence. Nucleic Acids Res 25:955-64.

51. Buchfink B, Xie C, Huson DH. 2015. Fast and sensitive protein alignment using DIAMOND. Nat Methods 12:59-60.
52. Tatusov RL, Natale DA, Garkavtsev IV, Tatusova TA, Shankavaram UT, Rao BS, Kiryutin B, Galperin MY, Fedorova ND, Koonin EV. 2001. The COG database: new developments in phylogenetic classification of proteins from complete genomes. Nucleic Acids Res 29:22-8.
53. Haft DH, Loftus BJ, Richardson DL, Yang F, Eisen JA, Paulsen IT, White O. 2001. TIGRFAMs: a protein family resource for the functional identification of proteins. Nucleic Acids Res 29:41-3.
54. Eddy SR. 2011. Accelerated Profile HMM Searches. PLoS Comput Biol 7:e1002195.
55. Cabanettes F, Klopp C. 2018. D-GENIES: dot plot large genomes in an interactive, efficient and simple way. PeerJ 6:e4958.
56. R Development Core Team. 2018. R: A language and environment for statistical computing, R Foundation for Statistical Computing.,
57. Stamatakis A. 2014. RAxML version 8: a tool for phylogenetic analysis and post-analysis of large phylogenies. Bioinformatics 30:1312-3.
58. Treangen TJ, Ondov BD, Koren S, Phillippy AM. 2014. The Harvest suite for rapid core-genome alignment and visualization of thousands of intraspecific microbial genomes. Genome Biol 15:524.
59. Alikhan NF, Petty NK, Ben Zakour NL, Beatson SA. 2011. BLAST Ring Image Generator (BRIG): simple prokaryote genome comparisons. BMC Genomics 12:402.
60. Page AJ, Cummins CA, Hunt M, Wong VK, Reuter S, Holden MT, Fookes M, Falush D, Keane JA, Parkhill J. 2015. Roary: rapid large-scale prokaryote pan genome analysis. Bioinformatics 31:3691-3.
61. Carattoli A, Zankari E, Garcia-Fernandez A, Voldby Larsen M, Lund O, Villa L, Moller Aarestrup F, Hasman H. 2014. In silico detection and typing of plasmids using PlasmidFinder and plasmid multilocus sequence typing. Antimicrob Agents Chemother 58:3895-903.
62. O'Brien FG, Yui Eto K, Murphy RJ, Fairhurst HM, Coombs GW, Grubb WB, Ramsay JP. 2015. Origin-of-transfer sequences facilitate mobilisation of non-conjugative antimicrobial-resistance plasmids in Staphylococcus aureus. Nucleic Acids Res 43:7971-83.
63. del Solar GH, Puyet A, Espinosa M. 1987. Initiation signals for the conversion of single stranded to double stranded DNA forms in the streptococcal plasmid pLS1. Nucleic Acids Res 15:5561-80.
64. Zuker M. 2003. Mfold web server for nucleic acid folding and hybridization prediction. Nucleic Acids Res 31:3406-15.
65. Arndt D, Grant JR, Marcu A, Sajed T, Pon A, Liang Y, Wishart DS. 2016. PHASTER: a better, faster version of the PHAST phage search tool. Nucleic Acids Res 44:W16-21.
66. Van Duyne GD, Rutherford K. 2013. Large serine recombinase domain structure and attachment site binding. Crit Rev Biochem Mol Biol 48:476-91.
67. Smith MC, Brown WR, McEwan AR, Rowley PA. 2010. Site-specific recombination by phiC31 integrase and other large serine recombinases. Biochem Soc Trans 38:388-94.
68. de Castro E, Sigrist CJ, Gattiker A, Bulliard V, Langendijk-Genevaux PS, Gasteiger E, Bairoch A, Hulo N. 2006. ScanProsite: detection of PROSITE signature matches and ProRule-associated functional and structural residues in proteins. Nucleic Acids Res 34:W362-5.
69. El-Gebali S, Mistry J, Bateman A, Eddy SR, Luciani A, Potter SC, Qureshi M, Richardson LJ, Salazar GA, Smart A, Sonnhammer ELL, Hirsh L, Paladin L, Piovesan D, Tosatto SCE, Finn RD. 2019. The Pfam protein families database in 2019. Nucleic Acids Res 47:D427-D432.
70. Marchler-Bauer A, Bo Y, Han L, He J, Lanczycki CJ, Lu S, Chitsaz F, Derbyshire MK, Geer RC, Gonzales NR, Gwadz M, Hurwitz DI, Lu F, Marchler GH, Song JS, Thanki N, Wang Z, Yamashita RA, Zhang D, Zheng C, Geer LY, Bryant SH. 2017. CDD/SPARCLE: functional classification of proteins via subfamily domain architectures. Nucleic Acids Res 45:D200-D203.

71. Perreten V, Chanchaithong P, Prapasarakul N, Rossano A, Blum SE, Elad D, Schwendener S. 2013. Novel pseudo-staphylococcal cassette chromosome mec element (psiSCCmec57395) in methicillin-resistant Staphylococcus pseudintermedius CC45. Antimicrob Agents Chemother 57:5509-15.
72. Varani AM, Siguier P, Gourbeyre E, Charneau V, Chandler M. 2011. ISsaga is an ensemble of web-based methods for high throughput identification and semi-automatic annotation of insertion sequences in prokaryotic genomes. Genome Biol 12:R30.
73. Huson DH, Bryant D. 2006. Application of phylogenetic networks in evolutionary studies. Mol Biol Evol 23:254-67.
74. Kumar S, Stecher G, Tamura K. 2016. MEGA7: Molecular Evolutionary Genetics Analysis Version 7.0 for Bigger Datasets. Mol Biol Evol 33:1870-4.
75. Gomez-Sanz E, Schwendener S, Thomann A, Gobeli Brawand S, Perreten V. 2015. First Staphylococcal Cassette Chromosome mec Containing a mecB-Carrying Gene Complex Independent of Transposon Tn6045 in a Macrococcus canis Isolate from a Canine Infection. Antimicrob Agents Chemother 59:4577-83.
76. Baig S, Johannesen TB, Overballe-Petersen S, Larsen J, Larsen AR, Stegger M. 2018. Novel SCCmec type XIII (9A) identified in an ST152 methicillin-resistant Staphylococcus aureus. Infect Genet Evol 61:74-76.
77. Schwendener S, Perreten V. 2015. New shuttle vector-based expression system to generate polyhistidine-tagged fusion proteins in Staphylococcus aureus and Escherichia coli. Appl Environ Microbiol 81:3243-54.
78. CLSI. 2018. Performance Standards for Antimicrobial Susceptibility Testing; Twenty-Eighth Informational Supplement (M100-S28). Clinical and Laboratory Standards Institute.
79. Kaiser TD, Pereira EM, Dos Santos KR, Maciel EL, Schuenck RP, Nunes AP. 2013. Modification of the Congo red agar method to detect biofilm production by Staphylococcus epidermidis. Diagn Microbiol Infect Dis 75:235-9.
80. Gomez-Sanz E, Ceballos S, Ruiz-Ripa L, Zarazaga M, Torres C. 2019. Clonally Diverse Methicillin and Multidrug Resistant Coagulase Negative Staphylococci Are Ubiquitous and Pose Transfer Ability Between Pets and Their Owners. Front Microbiol 10:485.
81. Schmelcher M, Shen Y, Nelson DC, Eugster MR, Eichenseher F, Hanke DC, Loessner MJ, Dong S, Pritchard DG, Lee JC, Becker SC, Foster-Frey J, Donovan DM. 2015. Evolutionarily distinct bacteriophage endolysins featuring conserved peptidoglycan cleavage sites protect mice from MRSA infection. J Antimicrob Chemother 70:1453-65.
82. Gomez-Sanz E, Torres C, Lozano C, Saenz Y, Zarazaga M. 2011. Detection and characterization of methicillin-resistant Staphylococcus pseudintermedius in healthy dogs in La Rioja, Spain. Comp Immunol Microbiol Infect Dis 34:447-53.
83. Gomez-Sanz E, Simon C, Ortega C, Gomez P, Lozano C, Zarazaga M, Torres C. 2014. First detection of methicillin-resistant Staphylococcus aureus ST398 and Staphylococcus pseudintermedius ST68 from hospitalized equines in Spain. Zoonoses Public Health 61:192-201.
