# Supplementary figures and images for: The Resistome and Mobilome of Multidrug-Resistant Staphylococcus sciuri C2865 Unveil a Transferable Trimethoprim Resistance Gene, Designated dfrE, Spread Unnoticed
Source: mSystems. 2021 Aug 10;6(4):e00511-21. doi: 10.1128/mSystems.00511-21 (PMC8407400; doi:10.1128/mSystems.00511-21)

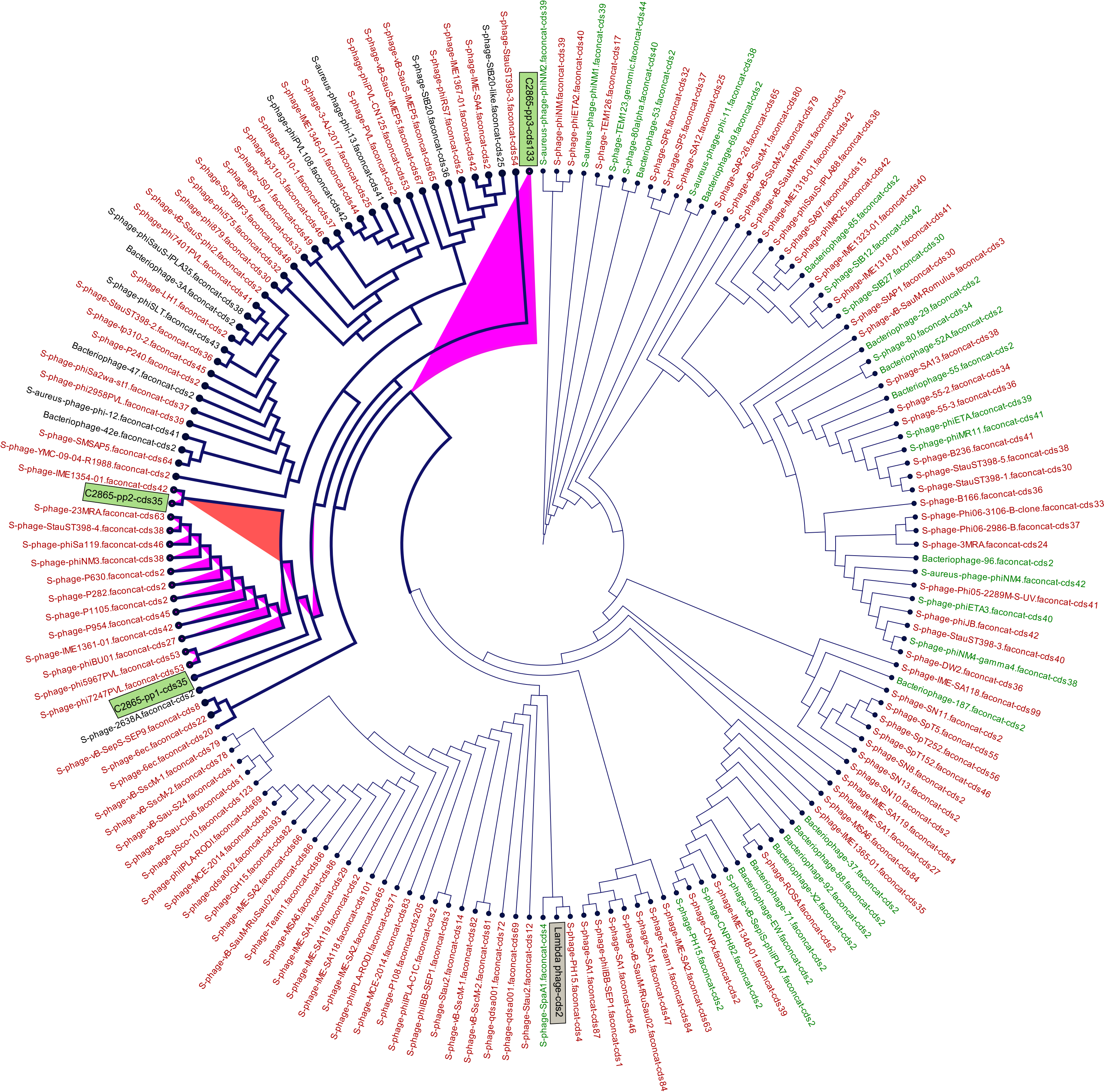

Supplement: FIG S8 [file msystems.00511-21-sf008.pdf]
