# Supplementary material for: The Resistome and Mobilome of Multidrug-Resistant Staphylococcus sciuri C2865 Unveil a Transferable Trimethoprim Resistance Gene, Designated dfrE, Spread Unnoticed
Source: mSystems. 2021 Aug 10;6(4):e00511-21. doi: 10.1128/mSystems.00511-21 (PMC8407400; doi:10.1128/mSystems.00511-21)
Supplement: FIG S7 [file msystems.00511-21-sf007.pdf]

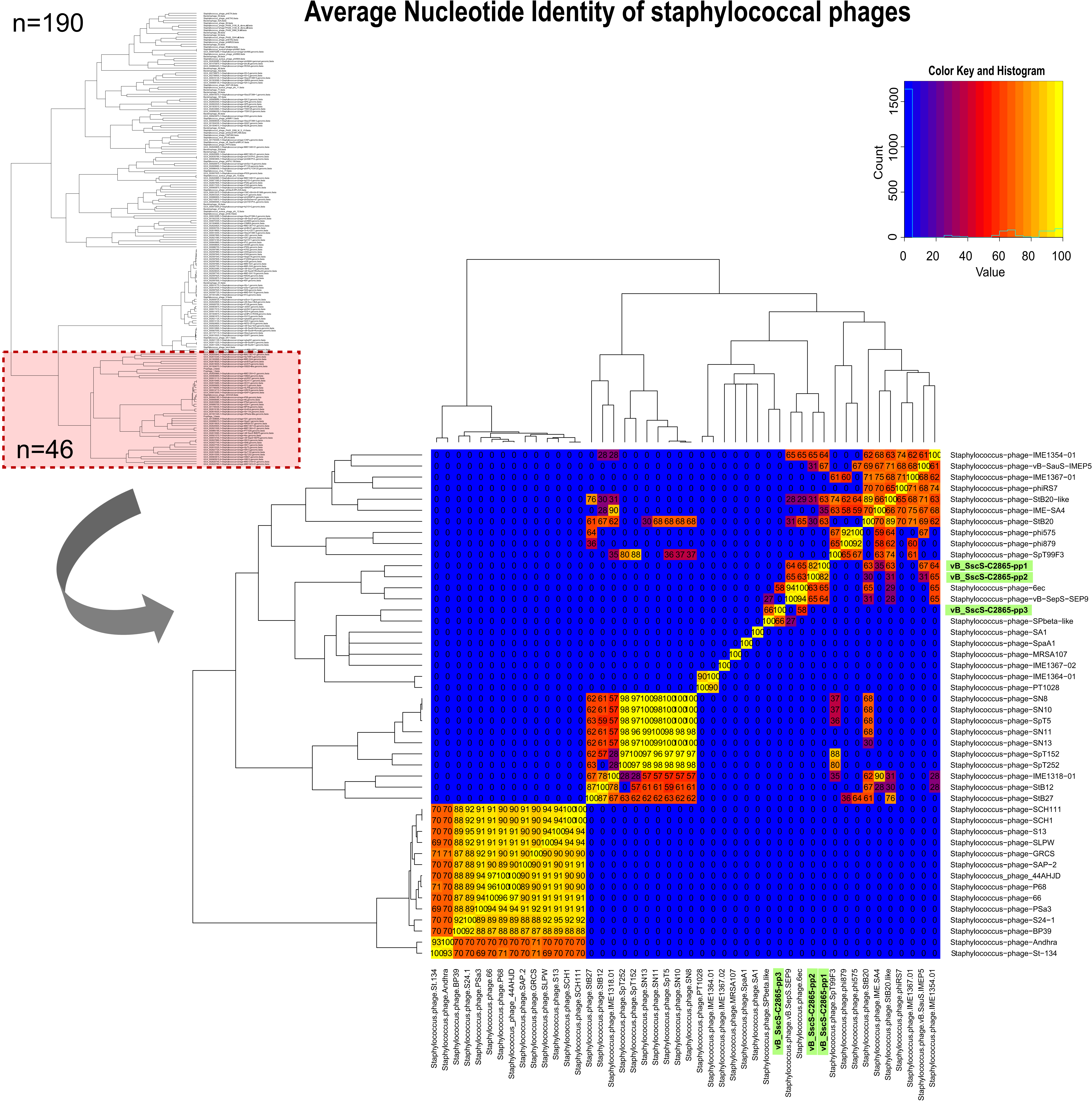
Average Nucleotide Identity of staphylococcal phages
n=190
n=46
Color Key and Histogram
Count
Value
0
20
40
60
80
100
0
500
1000
1500
Staphylococcus-phage-IME1354-01
Staphylococcus-phage-vB-SauS-IMEP5
Staphylococcus-phage-IME1367-01
Staphylococcus-phage-phiRS7
Staphylococcus-phage-StB20-like
Staphylococcus-phage-IME-SA4
Staphylococcus-phage-StB20
Staphylococcus-phage-phi575
Staphylococcus-phage-phi879
Staphylococcus-phage-SpT99F3
vB_SscS-C2865-pp1
vB_SscS-C2865-pp2
Staphylococcus-phage-6ec
Staphylococcus-phage-vB-SepS-SEP9
vB_SscS-C2865-pp3
Staphylococcus-phage-SPbeta-like
Staphylococcus-phage-SA1
Staphylococcus-phage-SpaA1
Staphylococcus-phage-MRSA107
Staphylococcus-phage-IME1367-02
Staphylococcus-phage-IME1364-01
Staphylococcus-phage-PT1028
Staphylococcus-phage-SN8
Staphylococcus-phage-SN10
Staphylococcus-phage-SpT5
Staphylococcus-phage-SN11
Staphylococcus-phage-SN13
Staphylococcus-phage-SpT152
Staphylococcus-phage-SpT252
Staphylococcus-phage-IME1318-01
Staphylococcus-phage-StB12
Staphylococcus-phage-StB27
Staphylococcus-phage-SCH111
Staphylococcus-phage-SCH1
Staphylococcus-phage-S13
Staphylococcus-phage-SLPW
Staphylococcus-phage-GRCS
Staphylococcus-phage-SAP-2
Staphylococcus_phage_44AHJD
Staphylococcus-phage-P68
Staphylococcus-phage-66
Staphylococcus-phage-PSa3
Staphylococcus-phage-S24-1
Staphylococcus-phage-BP39
Staphylococcus-phage-Andhra
Staphylococcus-phage-St-134
Staphylococcus.phage.St.134
Staphylococcus.phage.Andhra
Staphylococcus.phage.BP39
Staphylococcus.phage.S24.1
Staphylococcus.phage.PSa3
Staphylococcus.phage.66
Staphylococcus.phage.P68
Staphylococcus_phage_44AHJD
Staphylococcus.phage.SAP.2
Staphylococcus.phage.GRCS
Staphylococcus.phage.SLPW
Staphylococcus.phage.S13
Staphylococcus.phage.SCH1
Staphylococcus.phage.SCH111
Staphylococcus.phage.StB27
Staphylococcus.phage.StB12
Staphylococcus.phage.IME1318.01
Staphylococcus.phage.SpT252
Staphylococcus.phage.SpT152
Staphylococcus.phage.SN13
Staphylococcus.phage.SN11
Staphylococcus.phage.SpT5
Staphylococcus.phage.SN10
Staphylococcus.phage.SN8
Staphylococcus.phage.PT1028
Staphylococcus.phage.IME1364.01
Staphylococcus.phage.IME1367.02
Staphylococcus.phage.MRSA107
Staphylococcus.phage.SpaA1
Staphylococcus.phage.SA1
Staphylococcus.phage.SPbeta.like
vB_SscS-C2865-pp3
Staphylococcus.phage.vB.SepS.SEP9
Staphylococcus.phage.6ec
vB_SscS-C2865-pp2
vB_SscS-C2865-pp1
Staphylococcus.phage.SpT99F3
Staphylococcus.phage.phi879
Staphylococcus.phage.phi575
Staphylococcus.phage.StB20
Staphylococcus.phage.IME.SA4
Staphylococcus.phage.StB20.like
Staphylococcus.phage.phiRS7
Staphylococcus.phage.IME1367.01
Staphylococcus.phage.vB.SauS.IMEP5
Staphylococcus.phage.IME1354.01
